# Transcription factors AP-2α and AP-2β regulate distinct segments of the distal nephron in the mammalian kidney

**DOI:** 10.1038/s41467-022-29644-3

**Published:** 2022-04-25

**Authors:** Joseph O. Lamontagne, Hui Zhang, Alia M. Zeid, Karin Strittmatter, Alicia D. Rocha, Trevor Williams, Sheryl Zhang, Alexander G. Marneros

**Affiliations:** 1 Cutaneous Biology Research Center, Department of Dermatology, Massachusetts General Hospital and Harvard Medical School, Charlestown, MA 02129 USA; 2 Department of Craniofacial Biology, University of Colorado, Anschutz Medical Campus, Aurora, CO 80045 USA

**Keywords:** Differentiation, Ageing, Organogenesis

## Abstract

Transcription factors AP-2α and AP-2β have been suggested to regulate the differentiation of nephron precursor populations towards distal nephron segments. Here, we show that in the adult mammalian kidney AP-2α is found in medullary collecting ducts, whereas AP-2β is found in distal nephron segments except for medullary collecting ducts. Inactivation of AP-2α in nephron progenitor cells does not affect mammalian nephrogenesis, whereas its inactivation in collecting ducts leads to defects in medullary collecting ducts in the adult. Heterozygosity for AP-2β in nephron progenitor cells leads to progressive distal convoluted tubule abnormalities and β-catenin/mTOR hyperactivation that is associated with renal fibrosis and cysts. Complete loss of AP-2β in nephron progenitor cells caused an absence of distal convoluted tubules, renal cysts, and fibrosis with β-catenin/mTOR hyperactivation, and early postnatal death. Thus, AP-2α and AP-2β have non-redundant distinct spatiotemporal functions in separate segments of the distal nephron in the mammalian kidney.

## Abstract (web summary)

How the distal nephron is patterned during kidney development has been difficult to study. Here they show that AP-2β is required for the formation and postnatal function of distal convoluted tubules, whereas AP-2α has a role in maintaining the structure of medullary collecting ducts.

## Introduction

In the kidney, distal nephrons play key roles in regulating electrolyte homeostasis and urinary concentration. They consist of the thick ascending limbs of the loop of Henle (TALs), distal convoluted tubules (DCTs), connecting tubules (CTs), and collecting ducts (CDs). Which factors and molecular mechanisms regulate their development during nephrogenesis and their differentiation status in the adult is only partially understood. Based on single-cell RNA-Seq (scRNA-Seq) analyses of human embryonic kidneys, a model has been proposed in which Six2^+^ nephron progenitor cells (NPCs) differentiate into distinct nephrogenic lineages (podocyte and renal corpuscle precursors, proximal tubule [PT] precursors, loop of Henle [LOH] and distal tubule precursors) that further differentiate into specific nephron segments^1–3^. The scRNA-Seq data identified sets of genes that are found to be upregulated in specific nephron precursor populations and may, thus, play a role in directing the differentiation of those precursor populations into more differentiated specific nephron segments^1^. Previous studies in mouse mutants have identified some pathways and transcriptional regulators that are required for the differentiation of specific nephron segments. For example, specification of proximal-distal fates during nephron patterning involves Wnt, Bmp, Notch, and Fgf signaling pathways, as well as specific transcription factors (including Pou3f3, Lhx1, Irx2, Hnf1b, Foxc2, Mafb)^4–12^. However, the factors that orchestrate terminal differentiation of distal nephron segments remain largely unknown. It is likely that some of the factors identified in the scRNA-Seq data to be expressed in distal nephron precursor populations during nephrogenesis are critical regulators of distal nephron development. For example, these data show that the transcription factor AP-2α is expressed in distal nephron precursors in human embryonic kidneys, and its family member AP-2β is expressed in nephron segments giving rise to TALs and DCTs of the distal nephron^1^. Similarly, analysis of chromatin dynamics that occur during nephron progenitor differentiation in mouse embryonic kidneys suggests that AP-2α and AP-2β are regulating the transition from NPC-derived intermediate stage precursors into the distal nephron/LOH lineage^13^. These two transcription factors are also expressed in the distal nephron of the adult kidney^14^.

As AP-2 transcription factors play a role in epithelial differentiation processes in other tissues (e.g., epidermal differentiation)^15,16^, this raises the question of whether AP-2 transcription factors are also important for epithelial differentiation processes of the distal nephron. To test this, we inactivated AP-2β specifically in Six2^+^ NPCs (Six2Cre^+^Tfap2b^fl/fl^ mice), which leads to the inactivation of AP-2β activity in all nephron segments proximal to the CDs, and found that AP-2β is essential for the development of DCTs: Six2Cre^+^Tfap2b^fl/fl^ mice developed distal nephron precursors but failed to form DCTs during nephrogenesis^17^. We further demonstrated that AP-2β induces expression of KCTD1 in early-stage DCTs, which functions to promote terminal differentiation of early-stage DCTs into terminally differentiated DCTs. Inducible inactivation of AP-2β or KCTD1 in the adult shows that sustained activity of this AP-2β/KCTD1 axis in the adult kidney is required to maintain DCTs in a terminally differentiated state and that lack of either gene in the adult leads to partial dedifferentiation of DCTs that impairs their ability to concentrate urine^17,18^.

These findings raise the question of whether AP-2α plays a role in distal nephron development as well. Two recent studies with AP-2α and AP-2β zebrafish mutants suggested that AP-2α but not AP-2β is a critical regulator of distal nephron development in zebrafish^19,20^. These studies proposed that AP-2α acts upstream of AP-2β to regulate terminal differentiation of distal nephron segments that leads to the expression of solute transporters (such as *Slc12a1* [NKCC2, marker of TALs] and *Slc12a3* [NCC, marker of DCTs]). An AP-2α zebrafish mutant showed diminished AP-2β expression in the distal pronephros, whereas an AP-2β mutant did not affect AP-2α expression in the pronephros, indicating that AP-2α acts upstream of AP-2β in the zebrafish kidney^19^. Moreover, AP-2α loss-of-function in the zebrafish resulted in diminished expression of solute transporters (including NKCC2 and NCC), whereas AP-2β loss-of-function in zebrafish did not affect expression of these solute transporters^19^. Interestingly, combined loss-of-function of both AP-2α and AP-2β resulted in a greater reduction in *Slc12a3* expression than observed with only AP-2α loss, suggesting functional redundancy between these two AP-2 transcription factors in zebrafish. This functional redundancy may, in part, be explained by the observation that AP-2α and AP-2β can both homodimerize but also heterodimerize and have a high homology (AP-2α and AP-2β share an overall amino acid sequence identity of ~65% in zebrafish and of ~68% in mice)^21,22^. A subset of zebrafish lacking both AP-2α and AP-2β function also developed CD cysts, indicating that AP-2 transcription factors may be required for proper CD function as well^19^. Our observation that AP-2β deficiency in the mouse kidney leads to loss of DCTs and early postnatal lethality, whereas loss-of-function of AP-2β in the zebrafish does not result in distal nephron defects, demonstrates that AP-2 transcription factors have distinct functions for distal nephron development in mammalian versus zebrafish nephrogenesis. This raises the question of whether AP-2α is indeed a critical regulator of distal nephron development also in the mammalian kidney or whether this is only the case in the zebrafish kidney. Notably, AP-2α null mice die perinatally, associated with craniofacial abnormalities, anencephaly, and thoraco-abdominoschisis^23,24^. Kidney development has not been further investigated in these AP-2α null mice, and their perinatal lethality precludes evaluation of postnatal AP-2α function for the kidney. Thus, the role of AP-2α for mammalian nephrogenesis and renal function in the adult is unknown. In this work, we define the contributions of AP-2α versus AP-2β for mammalian kidney development and postnatal renal function by generating and characterizing mutant mice that are heterozygous or homozygous null for functional AP-2α or AP-2β specifically in NPCs (targeting the entire nephron proximal to the CDs using Six2Cre^+^ mice) or only in CTs and CDs (using Aqp2Cre^+^ mice).

## Results

### AP-2α and AP-2β are present in distinct distal nephron segments

**Fig. 1:**
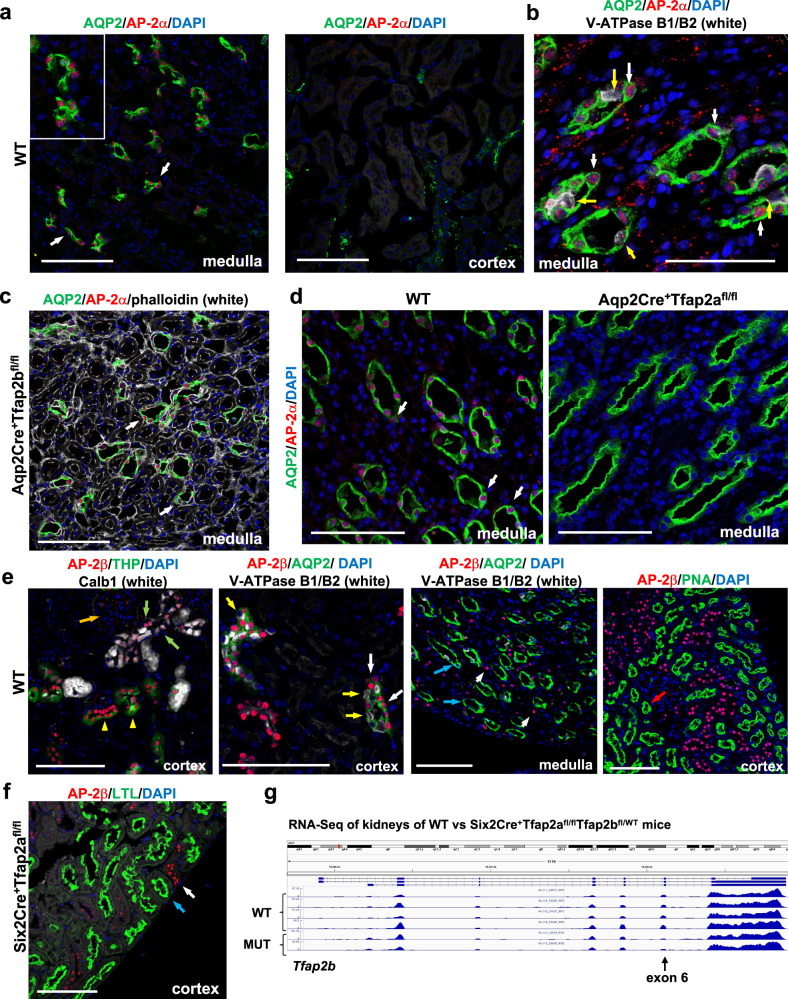
AP-2α and AP-2β proteins are present in distinct distal nephron segments of the adult mouse kidney, and their expression occurs independently of each other. **a** Immunolabeling for AP-2α shows nuclear localization in medullary Aqp2^+^ CDs (left [arrows]; inset shows magnified area) but not in cortical Aqp2^+^ CDs or CTs (right). Scale bars, 100 μm. **b** AP-2α protein localization to Aqp2^+^ principal cells of medullary CDs (white arrows), but no or low levels of AP-2α are detected in intercalated cells (V-ATPase B1/B2; yellow arrows) of medullary CDs. Scale bar, 50 μm. **c** Medullary CD localization of AP-2α is not affected by inactivation of AP-2β activity in Aqp2Cre^+^Tfap2b^fl/fl^ mice (white arrows). Scale bars, 100 μm. **d** Immunolocalization of AP-2α protein to Aqp2^+^ principal cells of medullary CDs, as seen in WT mice (white arrows), is lost in Aqp2Cre^+^Tfap2a^fl/fl^ mice. 5-months-old littermates. Scale bars, 100 μm. **e** Co-Immunolabelings for AP-2β with the anti-AP-2β antibody from Atlas in adult WT kidneys show that AP-2β is detected in TALs (THP^+^; yellow arrowheads), DCTs (Calb1^+^; green arrows), and cortical CTs/CDs (Aqp2^+^; yellow arrows) but not in glomeruli (orange arrow) or in PTs (PNA labeling; red arrow). In CTs/cortical CDs, AP-2β is detected in principal cells (Aqp2^+^; white arrows) but not in intercalated cells (V-ATPase B1/B2^+^; yellow arrows). In the inner medulla, medullary CDs (Aqp2^+^; blue arrows) show no immunolabeling for AP-2β that is only detected in adjacent non-CD nephron segments (short white arrows). Scale bars, 100 μm. **f** The distal nephron localization of AP-2β protein is not affected by the lack of AP-2α activity in these nephron segments (Six2Cre^+^Tfap2a^fl/fl^ mice; white arrow). PTs (LTL^+^) show no AP-2β immunolabeling (blue arrow). Immunolabeling with anti-AP-2β antibody from Cell Signaling Technology. Scale bars, 100 μm. **g** RNA-Seq data of whole kidney lysates from two 2-months-old Six2Cre^+^Tfap2a^fl/fl^Tfap2b^fl/WT^ mice and from four Cre-negative control littermates (WT) show that Cre activity effectively removes exon 6 of the *Tfap2b* gene, which is critical for DNA binding and activity of AP-2β. Transcription of the *Tfap2b* gene occurs despite the inactivation of AP-2α function in the same cells.

Analysis of three independent scRNA-Seq datasets derived from adult mouse kidneys shows that AP-2α is expressed at high levels predominantly in principal cells of medullary CDs in the adult, and only low-level expression is detected in intercalated cells of CDs (Ransick et al., 2019 [Supplementary Fig. S1; https://cello.shinyapps.io/kidneycellexplorer/]^14^; Humphreys laboratory [http://humphreyslab.com/SingleCell/search.php]; Park et al., 2018^25^). In contrast, AP-2β is expressed in the adult mouse kidney at high levels predominantly in thin ascending limbs of the loop of Henle, TALs, DCTs, as well as CTs and cortical CDs, but not in principal cells or intercalated cells of medullary CDs (Supplementary Fig. S1). Thus, AP-2α and AP-2β show a mostly non-overlapping expression pattern in the distal nephron of the adult mouse kidney. Similarly, our immunolabeling for AP-2α shows a strict nuclear localization in medullary CD cells that co-label for the principal cell marker Aqp2, but no AP-2α is detected in CTs, cortical CDs, or other nephron segments (Fig. 1a; Supplementary Fig. S2a, b). Moreover, compared to the strong immunolabeling for AP-2α in principal cells of medullary CDs, intercalated cells of CDs (V-ATPase B1/B2^+^) show either no or only low-level immunolabeling for AP-2α (Fig. 1b). Mice lacking functional AP-2β in CTs/CDs (Aqp2Cre^+^Tfap2b^fl/fl^ mice) show the same immunolocalization of AP-2α in medullary CDs as in WT mice, demonstrating that AP-2β is not required for AP-2α expression in medullary CDs (Fig. 1c; Supplementary Fig. S3a–c). Immunolabeling for AP-2α in kidneys of Aqp2Cre^+^Tfap2a^fl/fl^ mice shows complete loss of AP-2α protein in inner medullary CDs, validating the specificity of the antibody and the efficient inactivation of AP-2α in principal cells of medullary CDs in Aqp2Cre^+^Tfap2a^fl/fl^ mice (Fig. 1d; Supplementary Fig. S2a).

Consistent with the scRNA-Seq data, we find AP-2β protein to be localized in distal nephron epithelial cells, including TALs, DCTs, CTs, and cortical CDs, but not in medullary CDs, PTs, or glomeruli (Fig. 1e; Supplementary Fig. S4). Immunolabeling for AP-2β in mice lacking functional AP-2α in the nephron proximal to CDs (Six2Cre^+^Tfap2a^fl/fl^ mice) shows the same localization of AP-2β in distal nephron segments as in WT mice, demonstrating that AP-2α is not required for AP-2β expression in the distal nephron (Fig. 1f). We also confirmed this by RNA-Seq of whole kidneys of Six2Cre^+^Tfap2a^fl/fl^Tfap2b^fl/WT^ mice, which shows the presence of AP-2β transcripts in these kidneys despite inactivation of AP-2α (Fig. 1g). These RNA-Seq data also show a reduction of exon 6 due to conditional removal of the heterozygous floxed exon 6 that is required for transcriptional activity of AP-2β, confirming efficient Cre activity provided by the Six2Cre allele in nephron segments in which AP-2β is expressed in these mice (Fig. 1g). Thus, in contrast to the findings in zebrafish^19^, in the mouse kidney, AP-2α is not upstream of AP-2β and is not required for AP-2β expression. Collectively, these data show that AP-2α and AP-2β proteins are present in distinct distal nephron segments of the adult mouse kidney, consistent with the findings from the scRNA-Seq datasets, and that their expression occurs independently of each other. This suggests non-redundant and non-overlapping functions of these two transcription factors in the adult mammalian kidney.

### AP-2α regulates medullary CD structure in the adult

**Fig. 2:**
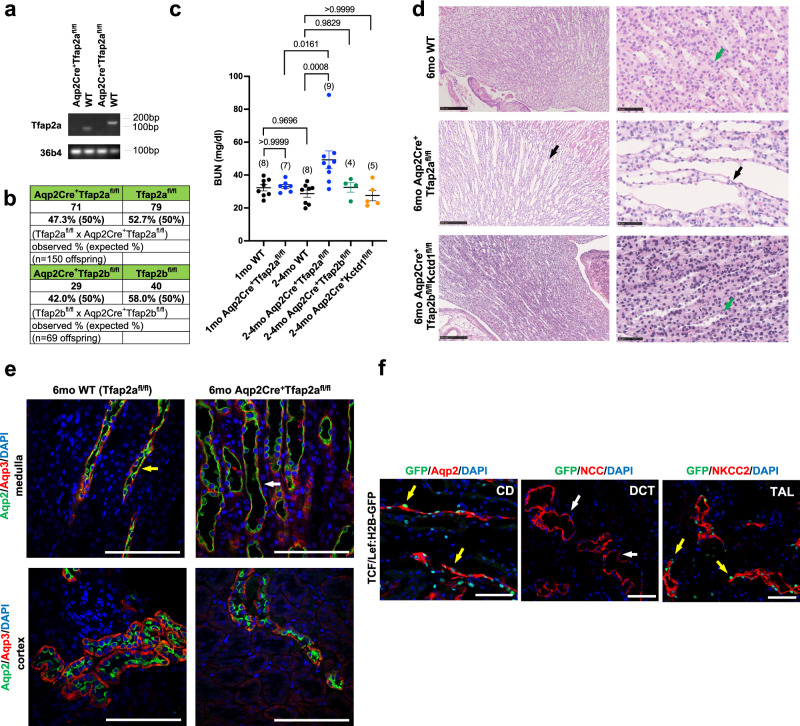
AP-2α regulates medullary CD structure in the adult but is not required for CD development. **a** RT-PCR of whole kidney lysates of Aqp2Cre^+^Tfap2a^fl/fl^ mice and Cre-negative littermates (WT) with two different PCR primer pairs that span exons 5/6 of *Tfap2a*. Kidneys of Aqp2Cre^+^Tfap2a^fl/fl^ mice show efficient inactivation of *Tfap2a*. 36b4 housekeeping gene. DNA ladder band size is indicated. **b** Aqp2Cre^+^Tfap2a^fl/fl^ mice and Aqp2Cre^+^Tfap2b^fl/fl^ mice show a normal Mendelian ratio at 4-weeks of age. **c** Serum BUN levels are normal in 1-month-old Aqp2Cre^+^Tfap2a^fl/fl^ mice but they are increased at 2–4 months of age, whereas at that age BUN levels remain normal in Aqp2Cre^+^Tfap2b^fl/fl^ mice or Aqp2Cre^+^KCTD1^fl/fl^ mice. Adjusted *p* values are shown (one-way ANOVA with Tukey’s multiple comparisons test). Graphs represent data as mean ± SEM. Number of mice per group is indicated in brackets. Source data are provided as a Source Data File. **d** Dilated medullary CDs with flattened epithelial cells (black arrows) in 6-months-old Aqp2Cre^+^Tfap2a^fl/fl^ mice, but not in WT littermates (Tfap2a^fl/fl^ mice) or Aqp2Cre^+^Tfap2b^fl/fl^Kctd1^fl/fl^ mice (green arrows). Scale bars, 250 μm left, and 50 μm right. **e** Immunolabeling for Aqp2 and Aqp3 show dilated medullary CDs in 6-months-old Aqp2Cre^+^Tfap2a^fl/fl^ mice (white arrow) compared to WT littermates (yellow arrow), whereas cortical CDs/CTs appear normal. Scale bars, 100 μm. **f** TCF/Lef:H2B-GFP mice serve as reporter mice for cellular β-catenin activity (GFP^+^ cells): GFP^+^ cells (yellow arrows) are detected in CDs (Aqp2^+^) in the medulla as well as in TALs (NKCC2^+^), whereas DCTs (NCC^+^) show no GFP^+^ cells (white arrows). 23-months-old TCF/Lef:H2B-GFP mouse kidney. Scale bars, 50 μm.

To assess the roles of AP-2α for medullary CDs during development and in the adult kidney, we inactivated AP-2α selectively in principal cells (Aqp2^+^) of CTs/CDs using Aqp2Cre mice. We confirmed that Aqp2Cre^+^ mice show Cre-mediated excision of floxed alleles only in CTs/CDs (Supplementary Fig. S5a)^17^. We generated Aqp2Cre^+^Tfap2a^fl/fl^ mice by crossing female Aqp2Cre^+^Tfap2a^fl/WT^ mice with male Tfap2a^fl/fl^ mice, in which loxP sites flank exons 5 and 6 that are required for dimerization and DNA binding activities of AP-2α^26^. Thus, Cre-mediated removal of floxed exons 5 and 6 of *Tfap2a* leads to loss of AP-2α transcription factor activity. Indeed, whole kidney lysates of adult Aqp2Cre^+^Tfap2a^fl/fl^ mice showed loss of *Tfap2a* transcripts that contain exons 5/6, confirming efficient Cre-mediated removal of these exons and, therefore, AP-2α transcription factor activity, as well as confirming that cells targeted by Aqp2Cre are the main source of *Tfap2a* transcripts in the adult mouse kidney (Fig. 2a). This is consistent with our immunolabeling data showing that AP-2α protein localization in inner medullary Aqp2^+^ CD cells is lost in kidneys of Aqp2Cre^+^Tfap2a^fl/fl^ mice (Fig. 1d).

Aqp2Cre^+^Tfap2a^fl/fl^ mice developed normally, had a normal Mendelian ratio and normal BUN levels at 4 weeks of age, and showed no histomorphological renal abnormalities at that age, including normal-appearing CTs/CDs (Fig. 2b, c; Supplementary Fig. S6a). However, at 2–4 months of age Aqp2Cre^+^Tfap2a^fl/fl^ mice showed a significant increase in BUN (Fig. 2c). Starting at that age, a dilatation of medullary CDs was observed as well (Fig. 2d, e; Supplementary Fig. S6b), whereas cortical CDs or CTs appeared normal (Fig. 2e; Supplementary Fig. S6c), consistent with the immunolocalization of AP-2α only in medullary but not in cortical CDs. The dilated medullary CDs of these Aqp2Cre^+^Tfap2a^fl/fl^ mice showed flattening of epithelium, which maintained its polarization with proper immunolocalization of Aqp2 towards the luminal site and of Aqp3 towards the basal site of CDs (Fig. 2d, e; Supplementary Fig. S6b). Body weight, 24-hour urine production, and urine osmolality, as well as urine and serum electrolyte concentrations, were within normal limits in adult Aqp2Cre^+^Tfap2a^fl/fl^ mice (Supplementary Fig. S7a; Tables S1 and S2). Moreover, Aqp2Cre^+^Tfap2a^fl/fl^ mice maintained the ability to concentrate urine to the same extent as their control littermates with 24-hour water deprivation and showed a similar body weight reduction with this water deprivation (Supplementary Fig. S7a). Aqp2Cre^+^Tfap2a^fl/fl^Tfap2b^fl/fl^ mice that lack activity of both transcription factors in CTs/CDs did also form normal-appearing CTs/CDs (Supplementary Fig. S6a). Lack of the AP-2β/KCTD1 axis in CTs/CDs did not affect the viability of the mice, the morphology of CTs/CDs, BUN levels, or 24-hour urine production and water intake (Fig. 2b–d; Supplementary Figs. S6a and S7b–d). These findings demonstrate that AP-2α is not required for the proper formation of CTs/CDs in the mouse kidney during development but plays a role in maintaining medullary CD structure in the adult. In contrast, the AP-2β/KCTD1 axis does not have critical functions for CTs/CDs during nephrogenesis and in the adult, and there is no major redundancy between AP-2α and AP-2β in CTs/CDs.

We reported that AP-2β induces KCTD1 expression in DCTs to maintain the terminal differentiation state of DCTs and suppress β-catenin signaling activity in this nephron segment in the adult^17^. Loss of KCTD1 in Six2Cre^+^KCTD1^fl/fl^ mice or KCTD1^–/–^ mice impairs terminal differentiation of DCTs and leads to β-catenin hyperactivation that promotes mTOR signaling and renal fibrosis^17^. Consistent with these data, our analysis of adult TCF/Lef:H2B-GFP mice, which serve as reporter mice for cellular β-catenin signaling activity, shows that DCTs do not have high-level β-catenin signaling activity (no GFP^+^ cells observed in DCTs) (Fig. 2f), whereas KCTD1 deficiency leads to GFP^+^ cells in DCTs^17^. Further analysis of TCF/Lef:H2B-GFP mice shows that Aqp2^+^ medullary CDs and NKCC2^+^ TALs of the adult mouse kidney have GFP^+^ cells (Fig. 2f). These findings raise the question of whether AP-2α affects β-catenin signaling activity in medullary CDs. Western blots of whole kidney lysates of Aqp2Cre^+^Tfap2a^fl/fl^ mice showed that protein levels of renal total β-catenin, active β-catenin (non-phospho Ser33/37/Thr41 β-catenin), and Aqp2 were not significantly different compared to age-matched littermates (Supplementary Fig. S8). Consistent with β-catenin hyperactivation being profibrotic and leading to renal fibrosis in mice lacking KCTD1 activity, kidneys of aged Aqp2Cre^+^Tfap2a^fl/fl^ mice that had no significant increase in active β-catenin levels did also not develop renal fibrosis (Supplementary Fig. 6c).

### Inactivation of AP-2α in Six2^+^ NPCs

**Fig. 3:**
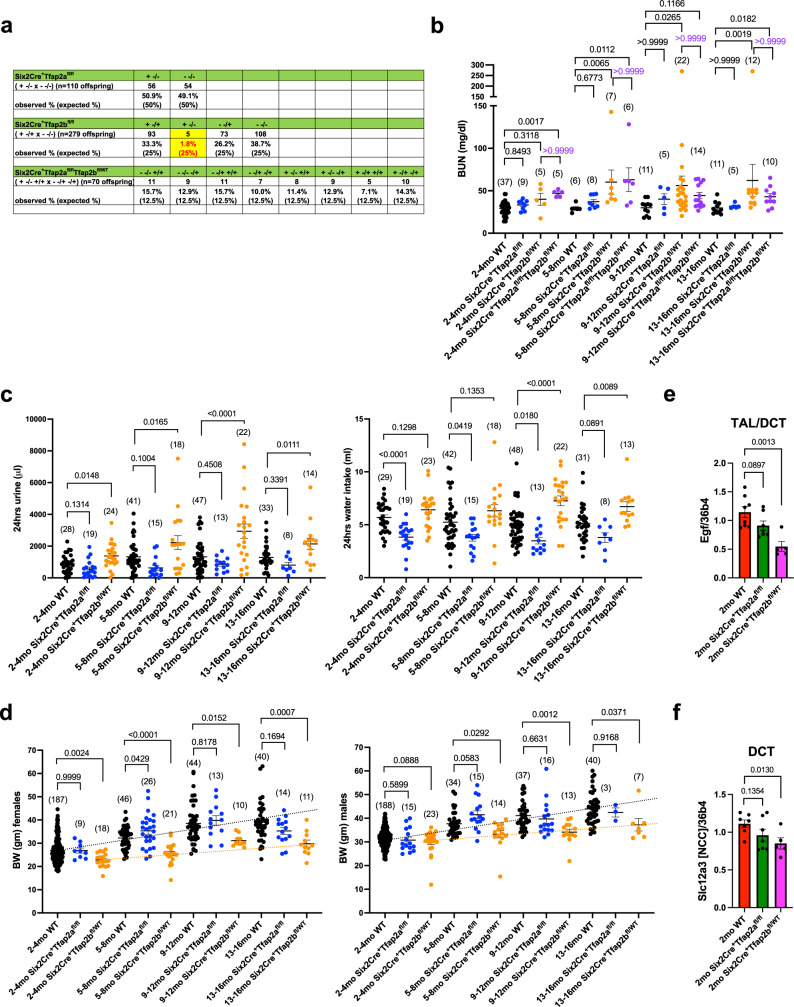
Progressive deterioration of kidney function in Six2Cre^+^Tfap2b^fl/WT^ mice but not in Six2Cre^+^Tfap2a^fl/fl^ mice. **a** Mendelian ratios in 4-weeks-old offspring from indicated crosses. Absolute numbers and percentiles are shown. Parentheses show expected Mendelian ratios. Deficiency of AP-2α function in the distal nephron proximal to the CDs in Six2Cre^+^Tfap2a^fl/fl^ mice does not affect the viability of the mice. In contrast, deficiency of AP-2β function leads to early postnatal lethality, and only a few mice survive to ~4–8 weeks of age (1.8% instead of the expected 25%). Heterozygosity for normal *Tfap2b* in Six2Cre^+^Tfap2b^fl/WT^ mice does not affect viability, even in the absence of normal *Tfap2a* in Six2Cre^+^Tfap2a^fl/fl^Tfap2b^fl/WT^ mice. Groups with the Cre allele are indicated as “+”, groups without the Cre allele as “−”. Heterozygosity or homozygosity for floxed alleles is indicated as “−/+” or “−/−” respectively (WT alleles “+/+”). **b** Measurements of BUN in Six2Cre^+^Tfap2a^fl/fl^ mice, Six2Cre^+^Tfap2b^fl/WT^ mice, Six2Cre^+^Tfap2a^fl/fl^Tfap2b^fl/WT^ mice, and Cre-negative WT littermates in different age groups up to 13-16 months of age. BUN is normal in Six2Cre^+^Tfap2a^fl/fl^ mice even with advanced age. In contrast, Six2Cre^+^Tfap2b^fl/WT^ mice and Six2Cre^+^Tfap2a^fl/fl^Tfap2b^fl/WT^ mice show a progressive deterioration of renal function with a steady increase in BUN with age progression. This increase is due to heterozygosity of AP-2β function, as the additional inactivation of AP-2α in these mice does not further exacerbate renal function. *P* values were determined by a Kruskal-Wallis test followed by Dunn’s multiple comparisons test. BUN in mg/dl. All graphs represent data as mean ± SEM. Each dot represents a different mouse. Number of mice per group is indicated in brackets. Source data are provided as a Source Data File. **c** The progressive age-dependent deterioration of renal function in Six2Cre^+^Tfap2b^fl/WT^ mice is accompanied by an increase in 24-h urine production (polyuria) and water intake (polydipsia), whereas Six2Cre^+^Tfap2a^fl/fl^ mice have no polyuria or polydipsia. Adjusted *p* values are shown (one-way ANOVA with Dunnett’s multiple comparisons test; comparisons to age-matched WT control groups). All graphs represent data as mean ± SEM. Each dot represents a different mouse. Number of mice per group is indicated in brackets. Source data are provided as a Source Data File. **d** An age-dependent progressive growth retardation is observed in both male and female Six2Cre^+^Tfap2b^fl/WT^ mice (yellow dotted line) but not in Six2Cre^+^Tfap2a^fl/fl^ mice when compared to WT littermates (black dotted line). Body weight (BW) shown in gm. Adjusted *p* values are shown (one-way ANOVA with Dunnett’s multiple comparisons test; comparisons to age-matched WT control groups). All graphs represent data as mean ± SEM. Each dot represents a different mouse. Number of mice per group is indicated in brackets. Source data are provided as a Source Data File. **e**, **f** Semiquantitative RT-PCR with whole kidney lysates of 2-months-old Six2Cre^+^Tfap2a^fl/fl^ mice (*n* = 7), Six2Cre^+^Tfap2b^fl/WT^ mice (*n* = 5), and WT littermates (*n* = 8 mice). Six2Cre^+^Tfap2a^fl/fl^ mice show normal renal expression of the terminal differentiation marker of TALs/DCTs EGF (**e**) and of the DCT marker Slc12a3 (NCC) (**f**). In contrast, heterozygosity for AP-2β function in kidneys of Six2Cre^+^Tfap2b^fl/WT^ mice leads to significantly reduced renal transcripts of *Egf* and *Slc12a3*. *P* values were determined by a two-tailed *t* test. Graphs represent data as mean ± SEM. Each dot represents the average value from triplicate experiments performed for each sample. Source data are provided as a Source Data File.

While AP-2α protein is not detected in the distal nephron other than the medullary CDs in the adult mouse kidney, scRNA-Seq data of embryonic kidneys show AP-2α expression in distal nephron precursors, suggesting a possible role of AP-2α for the development of distal nephron segments during nephrogenesis^1,13^. To assess whether AP-2α has a role in the development and function of nephron segments proximal to the CDs in the mammalian kidney, we generated mice in which AP-2α function is inactivated in Six2^+^ NPCs (Six2Cre^+^Tfap2a^fl/fl^ mice). We have previously independently confirmed published data showing that Six2Cre^+^ mice target floxed alleles efficiently in NPCs, resulting in gene inactivation in the entire nephron except for the CDs but including CTs^17,27–29^. Six2Cre^+^Tfap2a^fl/fl^ offspring were viable, were observed at the expected Mendelian ratios when assessed at 4-weeks of age (Fig. 3a), and had normal BUN levels (Fig. 3b) and normal renal histology even at an advanced age (Supplementary Fig. S9a). Moreover, Six2Cre^+^Tfap2a^fl/fl^ mice showed no increase in urine production or water consumption even at an advanced age (Fig. 3c) and maintained a normal body weight (Fig. 3d). EGF expression serves as a marker of TAL/DCT terminal differentiation^17^, and 2-months-old Six2Cre^+^Tfap2a^fl/fl^ mice showed normal renal expression of EGF or of the DCT marker NCC (Slc12a3) (Fig. 3e, f), and we observed normal co-localization of EGF and Pvalb in DCTs even in aged Six2Cre^+^Tfap2a^fl/fl^ mice (Supplementary Fig. S9b). Thus, in contrast to zebrafish, AP-2α does not have critical roles in mammalian nephrogenesis or for the function of nephron segments proximal to the CDs in the adult mouse kidney.

### Consequences of lack of AP-2β in NPCs

**Fig. 4:**
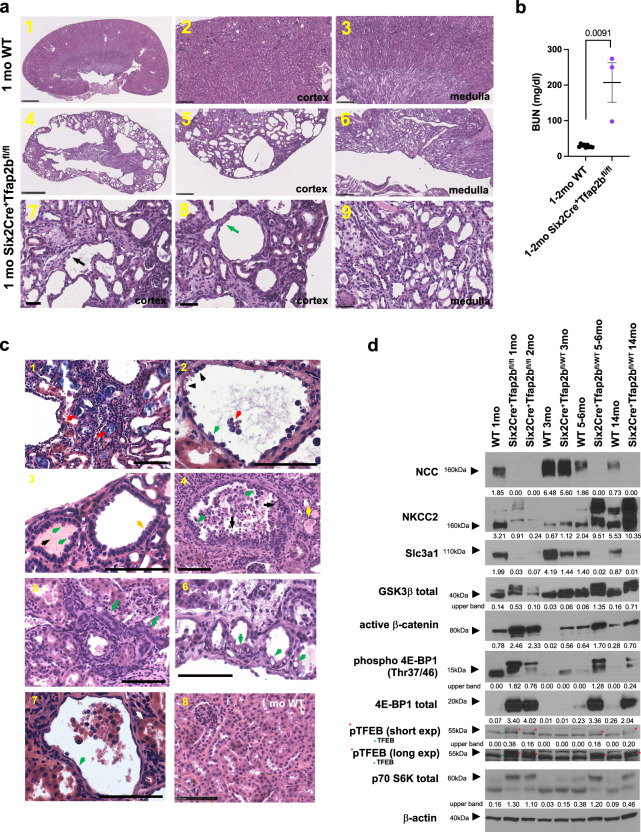
Deficiency of AP-2β in Six2^+^ NPCs results in severe cystic renal degeneration, kidney fibrosis, and renal failure. **a**, **b** Whereas most Six2Cre^+^Tfap2b^fl/fl^ mice died perinatally due to lack of DCTs, a few mice survived to 1–2 months of age, when they succumbed to renal failure with a very high BUN (**b**). These mice showed cystic degeneration of their renal cortex and tubulointerstitial fibrosis. Renal cysts were lined by either flattened epithelium (green arrow) or epithelial cells with a hobnail-like morphology (black arrow). H&E sections. Top: 1 month-old WT; middle and bottom panels: 1 month-old Six2Cre^+^Tfap2b^fl/fl^ mouse. Scale bars: 1,4: 1 mm; 2,3,5,6: 250 μm; 7-9: 50 μm. *P* value determined by a two-tailed Mann–Whitney test (**b**). BUN in mg/dl. Graph represents data as mean ± SEM. Source data are provided as a Source Data File. **b** *N* = 9 WT, n = 3 Six2Cre^+^Tfap2b^fl/fl^ mice. **c** H&E images show renal pathologies in 1–2 months-old Six2Cre^+^Tfap2b^fl/fl^ mice (1–7) compared to an age-matched littermate WT mouse (8). 1: Bluish calcified intratubular deposits are observed (red arrows). 2: Hobnail-like epithelial cells (green arrow) lining renal cysts with extrusion of cells into the lumen (red arrow). Some cells show clear cell-like appearance of their cytoplasm (black arrows). 3: Subset of dilated cortical distal nephron tubules show cuboidal epithelium (yellow arrow) with apical blebbing (green arrows). Protein casts are seen in some dilated tubules (black arrow). 4: In some dilated tubules, a multilayered epithelium (black arrows) and formation of micropapillae (green arrows) were observed. Inflammatory cells and extruded epithelial cells are seen in the lumen. Some tubules are filled with protein casts (yellow arrow). 5: In addition, atrophic tubules with diminished tubular lumen were observed (green arrows). Glomeruli appeared normal (white arrow). 6: A subset of epithelial cells of dilated distal tubules had a clear-cell-like appearance with cytoplasmic vacuoles (green arrows). 7: Some distal tubule cysts are lined by flattened epithelium (green arrow), whereas proximal tubules do not show epithelial flattening (white arrow). 8: WT littermates showed none of these pathologies. Scale bars, 100 μm. **d** Western blotting with whole kidney lysates of 1- and 2-months-old Six2Cre^+^Tfap2b^fl/fl^ mice, Six2Cre^+^Tfap2b^fl/WT^ mice (3, 5–6, and 14-months old), and WT littermate controls. Lack of AP-2β function results in absence of differentiated DCTs (the DCT marker NCC is not detected), whereas heterozygosity of AP-2β function leads to loss of NCC with age progression. This loss of NCC is accompanied by an increase in GSK-3β phosphorylation, in active non-phospho Ser33/37/Thr41 β-catenin, and in an increase in mTOR signaling (increased Thr37/46 phosphorylation of 4E-BP1 and total 4E-BP1; increased phosphorylation of p70 S6 kinase; increased TFEB phosphorylation [low and high exposure shown to demonstrate upper phospho-TFEB bands (red stars); lower TFEB bands marked with green stars]). Kidneys of Six2Cre^+^Tfap2b^fl/fl^ mice have diminished NKCC2, whereas aged Six2Cre^+^Tfap2b^fl/WT^ mice have increased NKCC2. Both have decreased protein levels of the PT protein Slc3a1. β-actin as a loading control. Densitometric values for Western blot bands normalized to β-actin are shown. Size markers are indicated by arrowheads.

We reported that AP-2β is essential for DCT formation during nephrogenesis, as lack of AP-2β in NPCs (Six2Cre^+^Tfap2b^fl/fl^ mice) results in lack of DCT formation and early postnatal lethality^17^. To assess whether any Six2Cre^+^Tfap2b^fl/fl^ mice that lack AP-2β activity in NPCs survived into early adulthood, we expanded our breedings with heterozygous Six2Cre^+^Tfap2b^fl/WT^ mice. We confirmed that even after a large number of offspring were assessed (279), only very few Six2Cre^+^Tfap2b^fl/fl^ mice survived to 1–2 months of age (1.8% of offspring survived to 4 weeks of age when the expected Mendelian ratio would be 25%) (Fig. 3a). These 1–2 months-old Six2Cre^+^Tfap2b^fl/fl^ mice showed severe histological kidney abnormalities with extensive distal nephron defects and cortical cyst formation (Fig. 4a) and succumbed to renal failure (highly increased BUN) within the first few weeks after birth (Fig. 4b). Distal nephron epithelia in the renal cortex of these mice showed abnormal epithelial morphology with a hobnail-like pattern and detachment of some epithelial cells (Fig. 4a, c). Other cysts or dilated distal nephron tubules showed flattened epithelium or epithelium with a cuboidal appearance or with apical epithelial blebbing, or a clear cell-type appearance of epithelial cells (Fig. 4a, c). Some atrophic tubules were observed as well (Fig. 4c). A subset of dilated distal nephron tubules showed a multilayered epithelium with areas of micropapillae formation (Fig. 4c). Moreover, intratubular calcium deposits and protein casts were observed in some dilated tubules (Fig. 4c). Glomeruli morphology appeared normal in these mice.

**Fig. 5:**
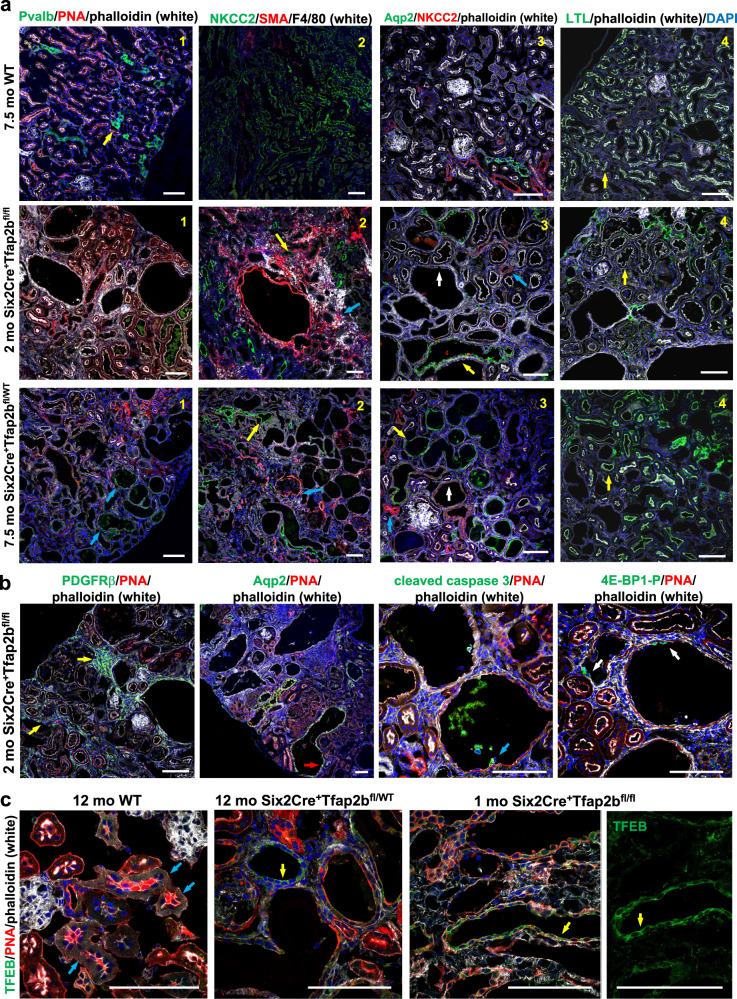
Effects of homozygous versus heterozygous loss of AP-2β activity in Six2^+^ NPCs. Immunolabeling of kidneys from a 2-months-old Six2Cre^+^Tfap2b^fl/fl^ mouse, a 7.5-months-old Six2Cre^+^Tfap2b^fl/WT^ mouse and a 7.5-months-old Cre-negative control mouse. Representative images are shown. **a** 1: Six2Cre^+^Tfap2b^fl/fl^ kidneys show an absence of DCTs (no Pvalb^+^ segments, which are seen in WT mice [yellow arrow]), whereas dilated Pvalb^+^ DCTs (blue arrows) are seen in kidneys of Six2Cre^+^Tfap2b^fl/WT^ mice. 2: NKCC2^+^ TALs (yellow arrows) are moderately dilated in kidneys of both Six2Cre^+^Tfap2b^fl/fl^ and Six2Cre^+^Tfap2b^fl/WT^ mice. Severe tubulointerstitial fibrosis with SMA^+^ myofibroblasts and a macrophage-rich (F4/80^+^) inflammatory infiltrate (blue arrows) is observed in kidneys of Six2Cre^+^Tfap2b^fl/fl^ mice and to a lesser extent in kidneys of Six2Cre^+^Tfap2b^fl/WT^ mice. 3: In Six2Cre^+^Tfap2b^fl/fl^ mice and Six2Cre^+^Tfap2b^fl/WT^ mice dilated CTs/cortical CDs (Aqp2^+^) are seen (yellow arrows), whereas NKCC2^+^ TALs show less or no dilatation (blue arrows). In addition, dilated tubules and cysts are observed that show no immunolabeling for Aqp2 or NKCC2 and do not show a brush border membrane that is typical of PTs (white arrows). 4: PTs (yellow arrows) show thinned and irregular brush border membranes (LTL^+^, strong signal with phalloidin labeling) in Six2Cre^+^Tfap2b^fl/fl^ mice and Six2Cre^+^Tfap2b^fl/WT^ mice. Scale bars, 100 μm. **b** Immunolabeling of a kidney from a 2-months-old Six2Cre^+^Tfap2b^fl/fl^ mouse (as in **a**) shows that renal fibrosis is accompanied by an abundance of PDGFRβ^+^ myofibroblasts (yellow arrows). Dilated cortical CDs/CTs (Aqp2^+^) are observed as well (red arrow). Dilated tubules show extrusion of apoptotic cells into the lumen (cleaved caspase-3^+^; blue arrow) and some cells with strong staining for phosphorylated 4E-BP1 (Thr37/46) (white arrows). Scale bars, 100 μm. **c** Immunolabeling for TFEB shows increased cytoplasmic staining in dilated distal nephron tubules in kidneys of Six2Cre^+^Tfap2b^fl/fl^ mice and Six2Cre^+^Tfap2b^fl/WT^ mice (yellow arrows), compared to the low staining intensity in distal nephron segments of WT mice (blue arrows). Scale bars, 100 μm.

Western blotting of whole kidney lysates confirmed the absence of DCTs in the kidneys of these 1-2 months-old Six2Cre^+^Tfap2b^fl/fl^ mice, with a complete lack of protein of the DCT differentiation marker NCC (Fig. 4d; Supplementary Fig. S10). Similarly, immunolabeling showed absence of DCTs in kidneys of Six2Cre^+^Tfap2b^fl/fl^ mice (no Pvalb^+^ nephron segments were detected), whereas TALs (NKCC2^+^), CTs/CDs (Aqp2^+^) and PTs (LTL^+^) were present (Fig. 5a). A subset of extensively dilated distal nephron tubules in the renal cortex of Six2Cre^+^Tfap2b^fl/fl^ mice and Six2Cre^+^Tfap2b^fl/WT^ mice were CTs (Calb1^+^Aqp2^+^) and cortical CDs (Aqp2^+^), whereas dilatation of TALs and PTs occurred to a lesser degree (Fig. 5a, b; Supplementary Fig. S11). Some cysts and extensively dilated tubules in these mice did not show immunolabeling for Pvalb (DCT marker), NKCC2 (TAL marker), Aqp2 (CT/CD marker), or CD133 (identifies PTs), consistent with defective distal nephron differentiation and/or progressive dedifferentiation as a consequence of reduced or absent AP-2β (Fig. 5a; Supplementary Fig. S12a).

Dilated tubules showed some Ki67^+^ cells (Supplementary Fig. S12a). Extensive tubulointerstitial fibrosis and a leukocytic (CD45^+^), macrophage (F4/80^+^)-rich inflammatory infiltrate were observed in the kidneys of these mice, with abundant immunolabeling for the myofibroblast/pericyte markers smooth muscle actin (SMA) and PDGFRβ (Fig. 5a, b; Supplementary Fig. S12a). Western blotting of whole kidney lysates of 1- and 2-months-old Six2Cre^+^Tfap2b^fl/fl^ mice showed that the severe fibrotic and cystic degeneration of the renal cortex also resulted in reduced markers of TALs (NKCC2) and PTs (Slc3a1), likely as a secondary consequence of the lack of DCTs or due to fibrosis (Fig. 4d; Supplementary Fig. S10).

Our previous work showed that AP-2β induces expression of KCTD1 that functions as a repressor of β-catenin signaling and that mice lacking KCTD1 develop progressive DCT defects and renal fibrosis accompanied by β-catenin hyperactivation and downstream profibrogenic mTOR activation^17^. Thus, we hypothesized that renal fibrosis in kidneys of Six2Cre^+^Tfap2b^fl/fl^ mice would be associated with increased β-catenin/mTOR activity. Indeed, fibrotic kidneys of 1-2 months-old Six2Cre^+^Tfap2b^fl/fl^ mice showed that lack of NCC was associated with increased activation of β-catenin and of mTOR downstream targets (p70 S6 kinase phosphorylation; 4E-BP1 [Thr37/46] phosphorylation, which was also detected by immunolabeling in epithelial cells lining dilated distal nephron segments) (Figs. 4d and 5b; Supplementary Fig. S10). Notably, increased levels of phosphorylated TFEB were observed in these kidneys (Fig. 4d; Supplementary Fig. S10), suggesting that mTOR hyperactivation leads to increased 4E-BP1 phosphorylation but not to translocation of TFEB to the nucleus (phosphorylation of TFEB sequesters it to the cytoplasm)^30^. Indeed, immunolabeling for TFEB shows increased cytoplasmic labeling in dilated distal nephron segments in kidneys of Six2Cre^+^Tfap2b^fl/fl^ mice (Fig. 5c). These findings confirm that AP-2β is absolutely critical for DCT formation during nephrogenesis and that lack of AP-2β leads to cortical renal fibrosis and cyst formation in the setting of β-catenin/mTOR hyperactivation, eventually resulting in renal failure.

### Inactivation of AP-2β selectively in DCT1s

**Fig. 6:**
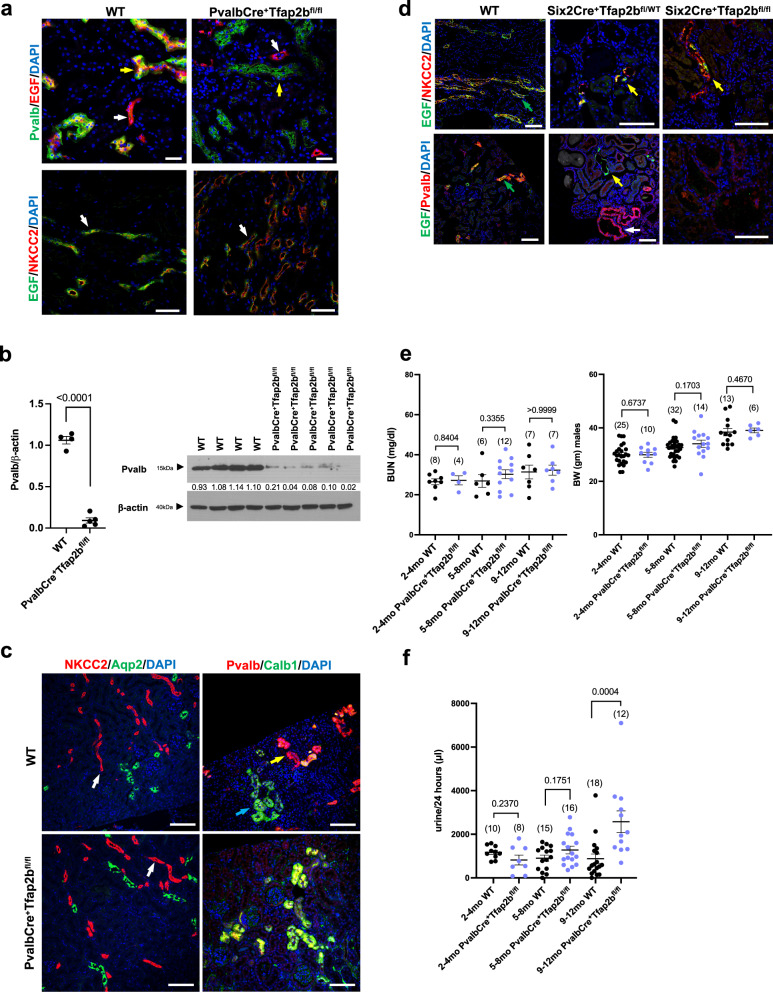
Inactivation of AP-2β in DCT1s impairs their terminal differentiation and function in the adult. **a** Lack of AP-2β function in DCT1s (PvalbCre^+^Tfap2b^fl/fl^ mice) leads to loss of EGF protein in DCT1s (Pvalb^+^; yellow arrows), whereas EGF immunoreactivity remains in TALs (NKCC2^+^; white arrows) that are not targeted by PvalbCre. 16-months-old mice. Scale bars, 50 μm. **b** Reduced Pvalb protein levels in whole kidney lysates of 12-months-old PvalbCre^+^Tfap2b^fl/fl^ mice compared to WT littermates. Values show densitometric values for Western blot bands normalized to β-actin. Size markers are indicated. *P* value was determined by a two-tailed *t* test. Graph represents data as mean ± SEM. *N* = 4 WT mice; *n* = 5 PvalbCre^+^Tfap2b^fl/fl^ mice. Source data are provided as a Source Data File. **c** Immunolabeling shows that PvalbCre^+^Tfap2b^fl/fl^ mice have normal-appearing TALs (NKCC2) (white arrows). Co-immunolabeling for Pvalb (DCT1 marker) and Calb1 (stronger expressed in CTs than in DCTs) identifies in WT mice CTs (stronger Calb1 staining compared to Pvalb) (blue arrows) and DCT1s (stronger Pvalb staining compared to Calb1) (yellow arrows). In PvalbCre^+^Tfap2b^fl/fl^ mice immunolabeling for Pvalb is reduced in DCT1s, leading to similar staining intensity for Calb1 and Pvalb in both nephron segments. 16-months-old mice. Scale bars, 100 μm. **d** Left: EGF immunolabeling is detected in a cytoplasmic localization in TALs (NKCC2^+^) and DCTs (Pvalb^+^) in WT mouse kidneys (green arrows). Middle: No or severely diminished EGF immunolabeling is detected in DCTs (Pvalb^+^ [red]; white arrow) of Six2Cre^+^Tfap2b^fl/WT^ mice (heterozygous for functional AP-2β in the kidney), whereas their TALs show EGF immunolabeling (NKCC2^+^; yellow arrows). Right: Six2Cre^+^Tfap2b^fl/fl^ mice (null for functional AP-2β in the kidney) lack DCTs, but their TALs show immunolabeling for EGF (yellow arrow). Scale bars, 100 μm. **e** Inactivation of *Tfap2b* in DCT1s (PvalbCre^+^Tfap2b^fl/fl^ mice) does not lead to an increase in BUN and does not affect body weight. *P* values were determined by a two-tailed Mann–Whitney test. BUN in mg/dl. Graphs represent data as mean ± SEM. Each dot represents a different mouse. Number of mice per group is indicated in brackets. Source data are provided as a Source Data File. **f** PvalbCre^+^Tfap2b^fl/fl^ mice show with progressive age (9-12 months) an increase in 24-hour urine production. *P* values were determined by a two-tailed Mann–Whitney test. Graphs represent data as mean ± SEM. Each dot represents a different mouse. Number of mice per group is indicated in brackets. Source data are provided as a Source Data File.

The DCT consists of two functional parts, the DCT1 (Pvalb^+^NCC^+^) and the DCT2 (Pvalb^negative^NCC^+^). To assess the role of AP-2β in DCT1s versus other distal nephron segments in which AP-2β is expressed, we inactivated AP-2β specifically in DCT1s by generating PvalbCre^+^Tfap2b^fl/fl^ mice. PvalbCre mice target floxed alleles only in DCT1s in the kidney and only after early-stage DCTs have already formed, which we confirmed in PvalbCre reporter mice (Supplementary Fig. S5b)^17,31^. In contrast, Six2Cre targets all nephron segments proximal to CDs and already at an earlier stage during nephrogenesis^27^. Whereas lack of AP-2β in early-stage DCTs (in Six2Cre^+^Tfap2b^fl/fl^ mice) leads to the absence of DCTs (Fig. 5a), inactivation of AP-2β at a later stage in DCT development after early-stage DCTs have already formed (PvalbCre^+^Tfap2b^fl/fl^ mice) and only in DCT1s (but not DCT2s) does not impair DCT formation (Fig. 6a; Supplementary Fig. S12b), consistent with a critical role of AP-2β for the transition of distal nephron precursors into early-stage DCTs^17^. However, we found that AP-2β is important to maintain the terminal differentiation state of DCT1s in the adult kidney, and lack of AP-2β selectively in DCT1s leads to reduced protein levels of the DCT marker NCC and the DCT1 marker Pvalb (Fig. 6b, c; Supplementary Fig. S12c). Immunolabeling also showed severely diminished protein levels of the terminal TAL/DCT differentiation marker EGF in DCT1s of PvalbCre^+^Tfap2b^fl/fl^ mice (Fig. 6a)^18^. Thus, AP-2β expression in DCT1s is required for EGF expression in this nephron segment. Notably, even heterozygosity for AP-2β is not sufficient for proper EGF expression in DCTs, as Six2Cre^+^Tfap2b^fl/WT^ mice showed diminished EGF immunolabeling in their dilated atypical DCTs (Fig. 5d). These Six2Cre^+^Tfap2b^fl/WT^ mice, as well as Six2Cre^+^Tfap2b^fl/fl^ mice, maintain at least some EGF immunolabeling in their TALs (Fig. 6d). Thus, EGF expression depends on AP-2β function in DCTs but can occur in TALs independently of AP-2β.

**Fig. 7:**
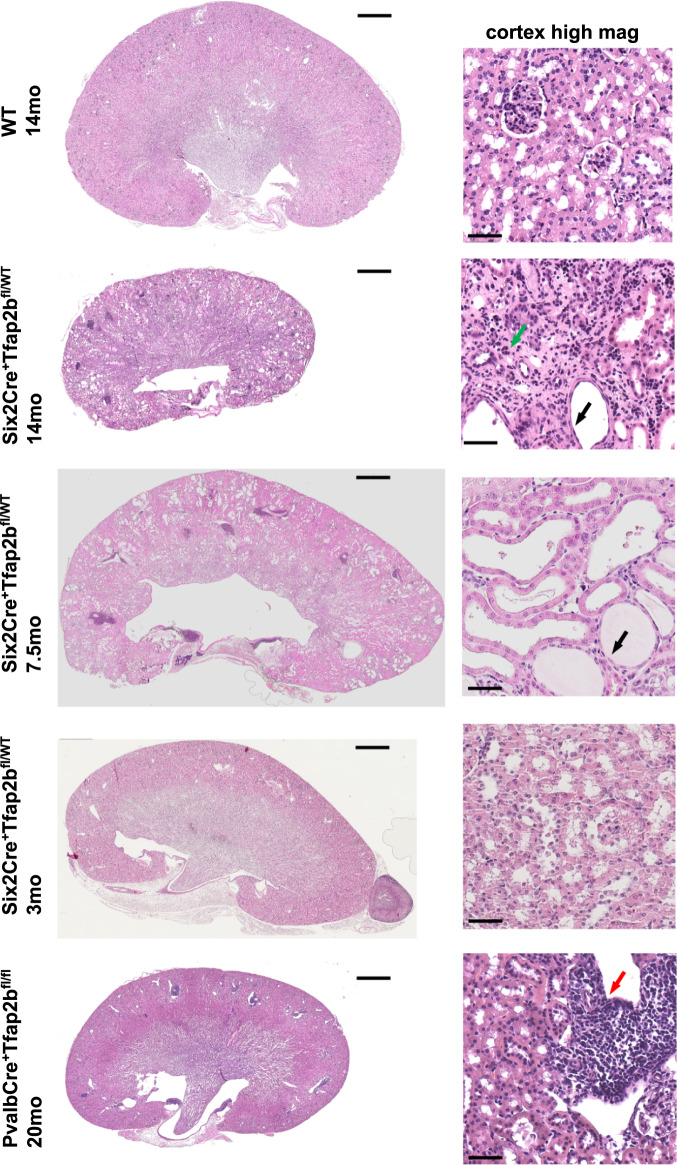
Progressive histomorphologic renal abnormalities in mice with heterozygous inactivation of AP-2β in NPC-derived nephron segments. H&E sections (left: whole kidney; right: high magnification image of the same kidney) show that Six2Cre^+^Tfap2b^fl/WT^ mice have a normal-appearing kidney at 3-months of age but develop severe distal nephron abnormalities with distal tubule dilatation (black arrows) and a tubulointerstitial inflammatory infiltrate (green arrow) at 7.5 and 14 months of age. In contrast, 20-monhts-old PvalbCre^+^Tfap2b^fl/fl^ mice show less severe abnormalities and inflammatory infiltrates (red arrow). Scale bars, 1 mm left and 50 μm right.

These findings raise the question of whether PvalbCre^+^Tfap2b^fl/fl^ mice develop progressive impairment of renal function and a reduced ability to concentrate urine with age progression. We find that aged PvalbCre^+^Tfap2b^fl/fl^ mice did not show progressive azotemia, and they maintained a normal body weight (Fig. 6e). However, with age progression, these mice had an increase in urine production (Fig. 6f), which is associated with reduced NCC levels (Fig. 6b) and hypomagnesemia in these mice (Tables S1 and S2)^18^, despite an absence of major histological abnormalities (Fig. 7). Thus, while inactivation of AP-2β only in DCT1s at a later stage of nephrogenesis leads to much less severe distal nephron defects with age progression than those observed in Six2Cre^+^Tfap2b^fl/fl^ mice, the findings in PvalbCre^+^Tfap2b^fl/fl^ mice show that AP-2β expression in DCT1s is important to maintain the fully functional terminal differentiation state of this nephron segment in the adult mouse kidney.

### AP-2β heterozygosity in NPCs results in progressive DCT defects

To test a gene-dosage effect of AP-2β for distal nephron function in the mammalian kidney, we generated mice that are heterozygous for AP-2β in the nephron proximal to CDs (Six2Cre^+^Tfap2b^fl/WT^ mice). Six2Cre^+^Tfap2b^fl/WT^ mice were viable (Fig. 3a) and showed the formation of all nephron segments (including Pvalb^+^ DCTs) without obvious histological kidney abnormalities in young adults (Fig. 7). Moreover, renal function was normal in young adult Six2Cre^+^Tfap2b^fl/WT^ mice (normal BUN, water intake, and urine production) (Fig. 3b, c). Thus, a single functional AP-2β allele is sufficient for the formation of DCTs, despite diminished EGF in the DCTs of these mice (Fig. 6d).

Similarly, Six2Cre^+^Tfap2a^fl/fl^Tfap2b^fl/WT^ mice that lack AP-2α activity and are also heterozygous for AP-2β in the nephron proximal to the CDs did not show renal abnormalities when assessed at P6 or in young adults, and formed all nephron segments without obvious defects and properly expressed markers of PTs, TAL, DCTs, or CTs/CDs (Fig. 3b; Supplementary Figs. S13a-e). In contrast, Six2Cre^+^Tfap2a^fl/fl^Tfap2b^fl/fl^ littermate mice lacking both AP-2α and AP-2β activity in the nephron proximal to the CDs had early postnatal lethality, postnatal growth retardation, absent DCTs (no immunolabeling detected for the DCT markers Pvalb and NCC), and dilated TALs (NKCC2^+^), as seen in Six2Cre^+^Tfap2b^fl/fl^ mice (Supplementary Fig. S13a-e)^17^. These findings are consistent with our observation that AP-2α deficiency does not affect the expression of AP-2β in the distal nephron and that Six2Cre^+^Tfap2a^fl/fl^ mice have normal kidneys. Thus, the lack of AP-2α in NPC-derived nephron segments does not induce or exacerbate renal abnormalities in mice heterozygous for AP-2β.

Notably, Six2Cre^+^Tfap2b^fl/WT^ mice showed a progressive impairment of renal function with advancing age, leading to an age-dependent increase in BUN and a deteriorating ability of their kidneys to concentrate urine, resulting in progressive polyuria and polydipsia (Fig. 3b, c). These functional renal abnormalities were associated with progressive postnatal growth retardation (Fig. 3d) and morphological TAL/DCT/CT defects in Six2Cre^+^Tfap2b^fl/WT^ mice (Figs. 5a, 6d, and 7; Supplementary Figs. S11 and S12a). While ~3-months-old Six2Cre^+^Tfap2b^fl/WT^ mice showed no major morphological kidney abnormalities, we observed extensive renal fibrosis, a tubulointerstitial macrophage-rich inflammatory infiltrate, and dilatation of TALs/DCTs/CTs in older Six2Cre^+^Tfap2b^fl/WT^ mice (e.g., 7.5 or 14 months-old mice) (Figs. 5d, 6d, and 7; Supplementary Figs. S10 and S11a). These changes were similar as seen in 1-2-months-old Six2Cre^+^Tfap2b^fl/fl^ mice, albeit to a lesser degree. Thus, functional loss of even a single *Tfap2b* allele is sufficient to cause progressive DCT defects in the adult, demonstrating a critical role of proper AP-2β levels for maintaining DCT function in the adult mammalian kidney.

**Fig. 8:**
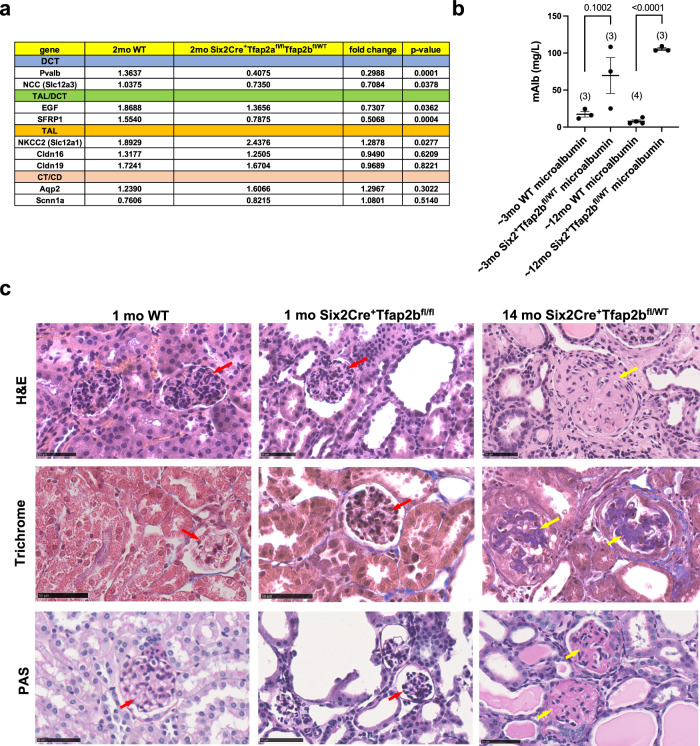
Severe glomerulosclerosis in aged Six2Cre^+^Tfap2b^fl/WT^ mice. **a** Semiquantitative RT-PCR with whole kidney lysates of 2-months-old Six2Cre^+^Tfap2a^fl/fl^Tfap2b^fl/WT^ mice and WT littermates. Expression of DCT markers *Pvalb* and *Slc12a3* is strongly reduced in kidneys of Six2Cre^+^Tfap2a^fl/fl^Tfap2b^fl/WT^ mice, as is the expression of genes that are expressed in both TALs and DCTs (*Egf*, *Sfrp1*). The TAL marker *Slc12a1* is upregulated in these kidneys, whereas the expression of CT/CD markers (*Aqp2*, *Scnn1a*) is unchanged. *N* = 9 kidneys per group. *P* values are shown (two-tailed, unpaired *t* test). Source data are provided as a Source Data File. **b** An increase in urinary microalbumin is observed in aged Six2Cre^+^Tfap2b^fl/WT^ mice. *P* values are shown (two-tailed *t* test). Graph represents data as mean ± SEM. Each dot represents a different mouse. Number of mice per group is indicated in brackets. Source data are provided as a Source Data File. **c** H&E, Trichrome, and PAS staining of kidneys form a 1-months-old WT mouse, a 1-months-old Six2Cre^+^Tfap2b^fl/fl^ mouse, and a 14-months-old Six2Cre^+^Tfap2b^fl/WT^ mouse. Whereas glomeruli show no glomerulosclerosis in the 1-months-old Six2Cre^+^Tfap2b^fl/fl^ mouse kidney (red arrows), eosinophilic, PAS^+^, and Trichrome^+^ staining is observed in glomeruli with strong expansion of the glomerular matrix in the 14-months-old Six2Cre^+^Tfap2b^fl/WT^ mouse kidney (yellow arrows), consistent with severe glomerulosclerosis. Scale bars, 50 μm.

Expression of the TAL/DCT terminal differentiation marker EGF and the DCT differentiation marker NCC (Slc12a3) were already significantly reduced in kidneys of 2-months-old Six2Cre^+^Tfap2b^fl/WT^ mice (Fig. 3e, f), consistent with the diminished immunolabeling for EGF in DCTs of Six2Cre^+^Tfap2b^fl/WT^ mice (Fig. 6d). Diminished EGF expression is, however, not a major cause for the progressive renal defects in these mice, as EGF deficiency in aged EGF^-/-^ mice did not phenocopy the renal abnormalities seen in Six2Cre^+^Tfap2b^fl/WT^ mice (Supplementary Fig. S13f). RNA-Seq of kidneys from adult Six2Cre^+^Tfap2a^fl/fl^Tfap2b^fl/WT^ mice showed that among the most downregulated genes were the DCT marker parvalbumin (Pvalb) and the TAL/DCT marker SFRP1 (Supplementary Fig. S13g). These findings were confirmed by semiquantitative RT-PCR of whole kidney lysates: kidneys of 2-months-old Six2Cre^+^Tfap2a^fl/fl^Tfap2b^fl/WT^ mice showed a significant decrease in the expression levels of DCT markers Pvalb and NCC (*Slc12a3*), as well as of genes expressed in both TALs and DCTs (*Egf* and *Sfrp1*), whereas expression of the TAL gene for NKCC2 (*Slc12a1*) was increased (Fig. 8a). Expression of other TAL genes (*Cldn16* and *Cldn19*) was normal, as was the expression of CT/CD marker genes (*Aqp2* and *Scnn1a*) (Fig. 8a).

Western blotting of whole kidney lysates of Six2Cre^+^Tfap2b^fl/WT^ mice at 3-, 5–6-, and 13-15-months of age shows that the observed age-dependent DCT defects and the azotemia in these mice are associated with progressive loss of NCC protein levels in these kidneys (Fig. 4d; Supplementary Fig. S14a). The worsening renal fibrosis is associated with a reduction of the PT marker Slc3a1, whereas NKCC2 is increased, likely to compensate for the diminished NCC function (Fig. 4d; Supplementary Fig. S14a). The progression in renal fibrosis and the reduction in renal NCC and Slc3a1 levels with advanced age in Six2Cre^+^Tfap2b^fl/WT^ mice was associated with an increase in β-catenin/mTOR hyperactivation (increased active β-catenin and increased phosphorylation of GSK-3β, p70 S6 kinase, 4E-BP1, and TFEB), similarly as observed in 1-2 months old Six2Cre^+^Tfap2b^fl/fl^ mice (Fig. 4d; Supplementary Figs. S14a–d). Overall, our findings demonstrate that heterozygosity for AP-2β leads to impaired terminal differentiation of DCTs, which is responsible for the progressive DCT abnormalities in these mice. Thus, DCTs of the mouse kidney are sensitive to loss of even one allele of the *Tfap2b* gene, and homozygosity for *Tfap2b* is required for the maintenance of proper DCT function in the adult.

Six2Cre^+^Tfap2b^fl/WT^ mice also had significant microalbuminuria that was associated with progressive glomerulosclerosis (Fig. 8b, c). Glomeruli in aged Six2Cre^+^Tfap2b^fl/WT^ mice showed eosinophilic, PAS^+^ and Trichrome^+^ staining of the glomerular matrix, which was not seen in young Six2Cre^+^Tfap2b^fl/WT^ mice, 1-2 months-old Six2Cre^+^Tfap2b^fl/fl^ mice, or control littermates (Figs. 7 and 8c). The glomerulosclerosis diminished the capillary space in these glomeruli, whereas glomerular capillaries appeared normal in 1-2 months-old Six2Cre^+^Tfap2b^fl/fl^ mice (Supplementary Fig. S15). In contrast, areas of tubulointerstitial fibrosis in 1-2 months-old Six2Cre^+^Tfap2b^fl/fl^ mice showed irregularly dilated CD31^+^ blood vessels (Supplementary Fig. S15). The occurrence of glomerulosclerosis in aged Six2Cre^+^Tfap2b^fl/WT^ mice but not in 1-2 months-old Six2Cre^+^Tfap2b^fl/fl^ mice, and the observation that glomeruli do not express AP-2β, suggests that the progressive glomerulosclerosis in Six2Cre^+^Tfap2b^fl/WT^ mice occurs as a secondary consequence of the chronic tubular and tubulointerstitial abnormalities in the kidneys of these mice. Thus, heterozygosity for functional AP-2β in the kidney leads also to distinct additional renal pathologies that are not observed in mice homozygous null for functional AP-2β in the kidney.

## Discussion

**Fig. 9:**
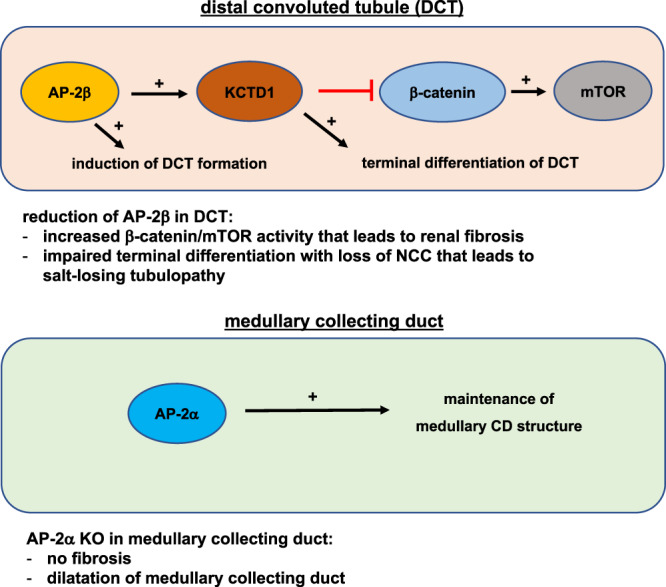
Proposed model for the distinct non-redundant roles of AP-2α and AP-2β for nephrogenesis and postnatal renal function in the mammalian kidney. AP-2β is critical for the formation of DCTs during nephrogenesis. Continued activity of the AP-2β/KCTD1 axis after nephrogenesis is necessary for maintaining terminal differentiation and function of DCTs, in part by repressing β-catenin/mTOR activity. Loss of even a single AP-2β allele leads to progressive DCT defects, β-catenin/mTOR hyperactivation, and renal fibrosis. In contrast, AP-2α is not required for distal nephron development but plays a role in maintaining the proper morphology of medullary CDs in the adult.

Our findings reveal a role of AP-2α in the maintenance of medullary CD structure in the adult mammalian kidney, whereas AP-2α is not required for the proper formation of nephron segments during nephrogenesis. Moreover, AP-2α was not required for AP-2β expression in the mouse kidney. These findings contrast the observations in zebrafish, where AP-2α has been suggested to be essential for proper distal nephron formation and to act upstream of AP-2β^19,20^. Moreover, while in zebrafish AP-2β loss did not result in distal nephron defects, we find that in mice AP-2β is critical for DCT formation and even loss of a single AP-2β allele leads to progressive DCT defects and impairment of renal function with aging as well as to glomerulosclerosis (Fig. 9). These observations show that caution must be exercised when drawing conclusions from renal development studies in zebrafish for mammalian nephrogenesis.

Heterozygous missense mutations in *TFAP2A* have been reported in patients with Branchio-Oculo-Facial syndrome (BOFS)^32^. About a third of these patients develop kidney abnormalities, which have been described clinically mainly as multicystic kidneys and vesicoureteral reflux but which have not been further characterized^32,33^. As AP-2α plays a role in nephric duct morphogenesis and urinary tract development^34^, these clinical observations in BOFS patients may be a consequence of defects in urinary tract development rather than nephrogenesis, consistent with the lack of renal abnormalities in Six2Cre^+^Tfap2a^fl/fl^ mice in which AP-2α is not inactivated in the urinary tract. Furthermore, renal abnormalities in BOFS patients may be in part also due to dilatation of CDs as a consequence of reduced AP-2α activity, as observed here in Aqp2Cre^+^Tfap2a^fl/fl^ mice.

Hyperactivation of the β-catenin pathway leads to increased TGF-β1 expression that can promote downstream activation of mTOR signaling. This increase in β-catenin and mTOR signaling, as well as TGF-β1 overexpression, has been linked to renal cyst formation and tissue fibrosis^35–43^. We have shown that AP-2β induces expression of the nuclear protein KCTD1, which induces terminal differentiation of DCTs^17^. Lack of KCTD1 resulted in β-catenin and mTOR hyperactivation, associated with increased expression of TGF-β1, and led to progressive renal fibrosis and cyst formation. Reducing β-catenin hyperactivation in KCTD1 mutants through heterozygosity for the β-catenin gene *Ctnnb1* attenuated mTOR hyperactivation and partially rescued the renal fibrosis and cyst formation phenotype in these mice^17^. Our findings in Six2Cre^+^Tfap2b^fl/fl^ mice that survived to 1-2 months of age further show that lack of AP-2β activity results in β-catenin/mTOR hyperactivation and severe renal fibrosis and cyst formation that leads to renal failure already at an early age. Moreover, loss of even a single AP-2β allele causes β-catenin/mTOR hyperactivation that is linked to renal fibrosis and cyst formation with age progression.

Activation of Rag GTPases by amino acids induces phosphorylation of S6 kinase, 4E-BP1, and the transcription factor TFEB by mTORC1, leading to activation of mTOR signaling^30^. TFEB phosphorylation sequesters this transcription factor in the cytoplasm^30^. In contrast, lack of functional folliculin, as observed in Birt-Hogg-Dubé syndrome, leads to hyperactivation of mTORC1-mediated phosphorylation of S6 kinase and 4E-BP1, but not of TFEB, which translocates to the nucleus when not phosphorylated and induces transcription of Rag C/D GTPases, further stimulating mTOR signaling activity^30^. The increased transcriptional activity of TFEB promotes not only renal cyst formation and renal fibrosis but also clear cell renal cancer at about 5 months of age, associated with β-catenin hyperactivation^44^. Notably, we find that lack of AP-2β leads to β-catenin hyperactivation and increased 4E-BP1 and p70 S6 kinase phosphorylation in the distal nephron that is associated with renal cyst formation, renal fibrosis, and histopathologic features that partly overlap with those observed in mice that overexpress TFEB in the renal tubular epithelium (multilayered epithelial cells, clear cells, micropapillae)^44^. Mice lacking AP-2β in the kidney did not show nuclear translocation of TFEB in distal nephron epithelial cells but instead showed increased phosphorylation of TFEB that leads to sequestration of TFEB in the cytoplasm. Whereas mice that overexpress TFEB in the kidney develop renal papillary carcinomas by about 5 months of age, we did not observe renal neoplasia in the examined 1-2-months-old Six2Cre^+^Tfap2b^fl/fl^ mice. This may be explained by either their early death due to renal failure that occurs before renal neoplasia can develop or by differences in the degree of mTOR hyperactivation between TFEB overexpressing mice and Six2Cre^+^Tfap2b^fl/fl^ mice.

Collectively, our findings reveal that AP-2β is not only critical for DCT formation during nephrogenesis but that DCT function in the adult mammalian kidney requires normal levels of AP-2β activity and that loss of even a single allele of AP-2β leads to progressive DCT differentiation defects that drive renal fibrosis and cyst formation. In contrast to zebrafish, AP-2β is not downstream of AP-2α, and its loss cannot be compensated for by AP-2α during mammalian nephrogenesis. Instead, AP-2α and AP-2β show a non-overlapping expression pattern in the adult mouse kidney, and AP-2α regulates medullary CD structure but is not essential for distal nephron or CD development (Fig. 9). In contrast, the AP-2β/KCTD1 axis does not have a critical function in CDs. These observations serve as an example that the closely related transcription factors AP-2α and AP-2β can have very different effects on differentiation processes and functions of distinct epithelial cell populations in the same organ.

## Methods

### Animals

Tfap2a^fl/fl^ mice have previously been reported: Cre activity results in the removal of exons 5 and 6 that are required for DNA binding activity^26^. Tfap2b^fl/fl^ mice have previously been described as well: Cre-mediated recombination results in the removal of exon 6, which is critical for DNA binding, resulting in AP-2β without transcription factor activity^45^. We showed by RNA-Seq of kidney lysates from TAM-treated β-actinCreERT2^+^Tfap2b^fl/fl^ mice efficient Cre-mediated removal of exon 6 of *Tfap2b*^17^. Similarly, we confirmed this by RNA-Seq of whole kidneys of Six2Cre^+^Tfap2a^fl/fl^Tfap2b^fl/WT^ mice that shows the presence of AP-2β transcripts in these kidneys with a reduction of exon 6 due to conditional removal of the heterozygous floxed exon 6 despite inactivation of AP-2α (Fig. 1g). The following Cre lines were used: Six2Cre^+^ mice [IMSR Cat# JAX:009606, RRID:IMSR_JAX:009606^27^], PvalbCre^+^ mice (IMSR Cat# JAX:017320, RRID:IMSR_JAX:017320)^46^, and Aqp2Cre^+^ mice [IMSR Cat# JAX:006881, RRID:IMSR_JAX:006881; using only female Aqp2Cre^+^ mice for matings]. Cre activity and specificity were confirmed by crossing these strains with B6.Cg-Gt(ROSA)26Sor^tm3(CAG-EYFP)Hze^/J (Ai3) reporter mice (IMSR Cat# JAX:007903, RRID:IMSR_JAX:007903)^17^. Co-immunolabeling with antibodies against GFP and Aqp2 showed strict co-localization in Aqp2Cre^+^Ai3 reporter mice, confirming that Cre activity in these mice is limited to CTs/CDs (Supplementary Fig. S5a)^17^. Co-immunolabeling with antibodies against GFP and Pvalb showed strict co-localization in PvalbCre^+^Ai3 reporter mice, confirming that Cre activity in these mice is limited to DCT1s (Supplementary Fig. S5b). We and others also validated that Six2Cre mice target the nephron proximal to the CDs, including the CTs^17,27,29^. EGF^-/-^ mice have previously been reported^47^. TCF/Lef:H2B-GFP reporter mice (IMSR Cat# JAX:013752, RRID:IMSR_JAX:013752) were used to assess β-catenin signaling activity in vivo^48^. We also inactivated AP-2α selectively in the epidermis with Keratin14Cre mice (IMSR Cat# JAX:018964, RRID:IMSR_JAX:018964), in order to validate the anti-AP-2α antibody in a tissue with well-established expression AP-2α (Supplementary Fig. S16)^15,16^. For all animal studies, institutional approval (MGH IACUC committee) was granted, and international guidelines for the care and use of laboratory research animals were followed. ARRIVE guidelines for reporting of animal studies were followed.

### Metabolic cages, serum, and urine chemistries

Single mice were kept for 24 hours in metabolic cages (3600M021, Techniplast), and urine and stool production, as well as water and food intake, were measured. All experimental mouse groups were exposed to metabolic cages in the same manner and under the same conditions. Mouse serum chemistries were determined with a Dri-Chem7000 chemistry analyzer (Heska) after blood was obtained via cardiac puncture after CO_2_ asphyxiation. Of note, this method of euthanasia leads to an artificial increase in serum potassium levels^49^. Urine chemistries were determined with a Roche ModP analyzer and urine osmolality with an Advanced Instruments Micro Osmometer Model 3300. Urine microalbumin was measured with a Siemens DCA Vantage analyzer. Values shown are from distinct mice.

### Immunolabeling of kidney sections and morphological kidney analyses

For all histological and immunolabeling experiments, at least 3 mice per experimental group (and at least 3 sections per mouse) were assessed in a blinded manner. For morphological analysis, mouse kidneys were bisected and fixed in 4% paraformaldehyde, and then processed and embedded in paraffin. H&E, PAS, and Trichrome stainings were performed according to standard protocols. The other kidney half was embedded in 30% sucrose and subsequently in OCT for immunolabeling experiments. For immunolabeling, either 7μm OCT kidney sections were used or 4 μm paraffin sections (after citrate buffer heat antigen retrieval) were used. Sections were permeabilized in 0.25% Triton X-100 and subsequently blocked with 5% serum from the species in which the secondary antibodies were raised. The following primary antibodies were used at a 1:100 dilution except where indicated: rabbit anti-AP-2α (Abcam Cat# ab108311, RRID:AB_10861200; 1:200 dilution; validated by absence of nuclear staining in inner medullary CDs of Aqp2Cre^+^Tfap2a^fl/fl^ mice or absence of nuclear staining in keratinocytes of Keratin14Cre^+^Tfap2a^fl/fl^ mice [Supplementary Fig. S16]), rabbit anti-AP-2β (Cell Signaling Technology Cat# 2509, RRID:AB_2058198; 1:200 dilution), rabbit anti-AP-2β (Atlas Antibodies Cat# HPA034683, RRID:AB_10670966), rat anti-F4/80 (conjugated with Alexa647, BioLegend Cat# 123121, RRID:AB_893492), rabbit anti-Slc3a1 (Proteintech Cat# 16343-1-AP, RRID:AB_2239419), mouse anti-Calb1 (Sigma Aldrich Cat# C9848, RRID:AB_476894; 1:300 dilution), goat anti-Aqp2 (Santa Cruz Biotechnology Cat# sc-9882, RRID:AB_2289903; 1:500 dilution), rabbit anti-NCC (Millipore Cat# AB3553, RRID:AB_571116), rabbit anti-NKCC2 (Cell Signaling Technologies Cat# 38436, RRID:AB_2799134), rabbit anti-parvalbumin (Abcam Cat# ab11427, RRID:AB_298032; 1:200 dilution), rat anti-PDGFRβ (Thermo Fisher Scientific Cat# 14-1402-82, RRID:AB_467493), rat anti-CD45 (BD Pharmingen (550539)), rat anti-CD133 (14-1331-80, ebioscience; 1:200 dilution), rabbit anti-TFEB (Bethyl Cat# A303-672A, RRID:AB_11204598; 1:200 dilution), rabbit anti-Aqp3 (Sigma-Aldrich Cat# A0303, RRID:AB_257878; 1:200 dilution), goat anti-EGF (R&D Systems Cat# AF2028, RRID:AB_355111), rabbit anti-phospho 4E-BP1 (Thr37/46) (Cell Signaling Technology Cat# 2855, RRID:AB_560835; 1:200 dilution), rabbit anti-cleaved caspase-3 (Asp175) (Cell Signaling Technology Cat# 9664, RRID:AB_2070042), chicken anti-GFP (Rockland Cat# 600-901-215, RRID:AB_1537402; 1:200 dilution), rabbit anti-GFP (Thermo Fisher Scientific Cat# A-11122, RRID:AB_221569), rabbit anti-Ki67 (Abcam Cat# ab16667, RRID:AB_302459), Cy3-conjugated mouse monoclonal SMA (clone 1A4) (Sigma-Aldrich Cat# A2547, RRID:AB_476701: 1:400 dilution), rat anti-CD31 (BD Biosciences Cat# 550274, RRID:AB_393571), and Alexa647-conjugated mouse anti-V-ATPase B1/B2 antibodies (Santa Cruz Biotechnology Cat# sc-55544 AF647, RRID:AB_831844). Phalloidin conjugated with Alexa488 (Molecular Probes Cat# A-12379, RRID:AB_2315147) or Alexa647 (Thermo Fisher Scientific Cat# A22287, RRID:AB_2620155) were used for cytoskeletal staining at a dilution of 1:100. DAPI was used to stain nuclei (Thermo Fisher Scientific Cat# D3571, RRID:AB_2307445). Secondary Alexa-488/555/647 antibodies were used at a dilution of 1:200 (Thermo Fisher). Controls included stainings with no primary antibody or with IgG control primary antibodies. Lectins at a dilution of 1:200 were from Vector Laboratories: rhodamine-conjugated peanut agglutinin [PNA] (Vector Laboratories Cat# RL-1072, RRID:AB_2336642) [epithelial staining of distal nephron epithelial cells; contiguous staining of PTs], fluorescein-conjugated Lotus Tetragonolobus Lectin [LTL] (Vector Laboratories Cat# FL-1321, RRID:AB_2336559) [labels PTs], rhodamine-conjugated wheat germ agglutinin [WGA] (Vector Laboratories Cat# RL-1022, RRID:AB_2336871). Immunolabeling for the different nephron segment markers has been consistent irrespective of age in kidneys of adult WT mice.

### Western blotting

Kidneys were lysed in NP40 lysis buffer (Life Technologies) with 1 mM PMSF and protease inhibitor cocktail (Complete, Roche) using the Qiagen TissueLyser-II. After centrifugation, the protein concentrations in the supernatant were determined with a Bradford assay. Equal amounts of protein were loaded onto NuPage 4-12% Bis-Tris gels (Life Technologies) and blotted to nitrocellulose membranes. Equal protein loading was assessed using a rabbit polyclonal anti-β-actin antibody (Cell Signaling Technology Cat# 4970, RRID:AB_2223172). The following primary antibodies were used at a 1:1000 dilution except where indicated: goat anti-Aqp2 (Santa Cruz Biotechnology Cat# sc-9882, RRID:AB_2289903; 1:200 dilution), rabbit anti-NKCC2 (38436, Cell Signaling Technology), rabbit anti-NCC (Millipore Cat# AB3553, RRID:AB_571116; 1:2000), rabbit anti-active β-catenin (non-phospho Ser33/37/Thr41) (Cell Signaling Technology Cat# 8814 S, RRID:AB_11127203), rabbit anti-total GSK-3β (Cell Signaling Technology Cat# 9315, RRID:AB_490890), rabbit anti-total GSK-3β (Cell Signaling Technology Cat# 12456, RRID:AB_2636978), rabbit anti-total 4E-BP1 (Cell Signaling Technology Cat# 9644, RRID:AB_2097841), rabbit anti-phospho 4E-BP1 (Thr37/46) (Cell Signaling Technology Cat# 2855, RRID:AB_560835), rabbit anti-p70 S6 kinase (49D7) (Cell Signaling Technology Cat# 2708, RRID:AB_390722), rabbit anti-TFEB (Bethyl Cat# A303-672A, RRID:AB_11204598), rabbit anti-Pvalb (Abcam Cat# ab11427, RRID:AB_298032; 1:2000 dilution), and rabbit anti-Slc3a1 antibodies (Proteintech Cat# 16343-1-AP, RRID:AB_2239419; 1:800 dilution). HRP-conjugated secondary antibodies were used at a 1:2000 dilution (HRP-linked anti-rabbit IgG from Cell Signaling Technology Cat#7074 S, RRID:AB_2099233; HRP-linked anti-goat IgG from Santa Cruz Biotechnology Cat#sc-2020, RRID_AB_631728) and chemiluminescence signal was determined with the SuperSignal WestPico chemiluminescent substrate (Pierce). Quantitation of Western blot bands was performed with Image J.

### RNA-Seq and semiquantitative RT-PCR

RNA was isolated from kidneys of 2-months-old Six2Cre^+^Tfap2a^fl/fl^Tfap2b^fl/WT^ mice and Cre-negative littermate controls with Trizol reagent (Life technologies). For RNA-Seq experiments, RNA was further purified with the Directzol RNA MiniPrep kit from Zymo (R2050) (*n* = 3/group). For library construction, 200 ng of total RNA using the NEBNext Ultra Directional RNA Library Prep Kit for Ilumina (E7420L) was used. 15 cycles of PCR were done. Quality control of libraries was done via Bioanalyzer High Sensitivity DNA and Tape Station High Sensitivity D5000 Screen Tape. Quantification of the samples was done via qPCR using the BioRad CFX96. The reagent kit for qPCR was the Kapa Library Quantification Kit for Illumina (KK4824). Sequencing was performed on an Illumina HiSeq 2500 instrument, resulting in approximately 30 million of 50 bp reads per sample. For gene expression studies, cDNA was obtained from kidneys of experimental mouse groups (*n* = 6 mice/group) using the Transcriptor First Strand synthesis kit utilizing hexamer primers (Roche). Semiquantitative RT-PCR was performed using a LightCycler 480 system with the LightCycler 480 SYBR Green I master mix according to standard procedures (45 amplification cycles) (Roche Applied Science). Primers for 36b4 were used as a normalization control. We previously reported the primer pair sequences used for semiquantitative RT-PCRs^17,18^. Concentrations were determined using a standard dilution curve. Experiments for all samples were performed in triplicate. RT-PCR was performed with RNA isolated from kidney lysates of Aqp2Cre^+^Tfap2a^fl/fl^ mice and Cre-negative littermates (WT). PCR amplification of cDNA was performed with two different PCR primer pairs that span exons 5/6 of *Tfap2a*, and for 36b4 as a housekeeping gene. The following primer pairs spanning exons 5/6 of *Tfap2a* were used: AGCAGGGAGACGTAAAGCTG and CAAAGTCCCTGGCTAGGTGG (primer pair #1) and GGGAGACGTAAAGCTGCCAA and TGCCACTTGCTCATTGGGAT (primer pair #2).

### Quantification and statistical analysis

#### RNA-Seq data analysis

Sequencing reads were mapped in a splice-aware fashion to the Ensembl annotation of the mouse GRCm37/mm9 transcriptome using STAR^50^. Read counts over transcripts were calculated using HTSeq^51^, followed by the differential expression analysis using EdgeR^52^. Genes were claddified as differentially expressed (DEGs) based on the cutoffs of 1.5-fold change in expression value and false discovery rates (FDR) below 0.05. RNA-Seq data have been deposited to the GEO database: GSE126326 and GSE130864.

#### Statistics and Reproducibility

For comparisons between two groups with non-normal data distribution, an unpaired two-tailed Mann–Whitney test was used for calculation of *p* values. For comparisons between two groups with normally distributed datasets, an unpaired two-tailed Student’s *t* test was used for statistical analyses. For multiple comparisons between normally distributed datasets, a one-way ANOVA with Tukey’s multiple comparisons test was performed. For multiple comparisons of normally distributed datasets to a control group, a one-way ANOVA with Dunnett’s multiple comparisons test was performed. For multiple comparisons of non-normally distributed datasets, a Kruskal-Wallis test followed by Dunn’s multiple comparisons test was performed. *P* values < 0.05 were considered to be statistically significant. *P* values are indicated. All graphs show mean ± SEM. Statistical analyses were performed with Prism 9.3.1 (Graphpad).

For all histological and immunolabeling experiments, at least 3 mice per experimental group (and at least 3 sections per mouse) were assessed in a blinded manner. Each value in mouse data graphs is derived from a distinct mouse.

### Reporting summary

Further information on research design is available in the Nature Research Reporting Summary linked to this article.

## Supplementary information

Supplementary Information

Reporting Summary

## Data Availability

RNA-Seq data have been deposited to the GEO database: GSE126326 and GSE130864. Source data are provided with this paper. For RNA-Seq data analysis, sequencing reads were mapped in a splice-aware fashion to the Ensembl annotation of the mouse GRCm37/mm9 transcriptome.

## Source data

Source Data

## Materials availability

Mouse lines generated in this study are available upon request. Source data are provided with this paper.
