# Supplementary material for: Transcription factors AP-2α and AP-2β regulate distinct segments of the distal nephron in the mammalian kidney
Source: Nat Commun. 2022 Apr 25;13:2226. doi: 10.1038/s41467-022-29644-3 (PMC9038906; doi:10.1038/s41467-022-29644-3)
Supplement: Supplementary file 1 — Supplementary Information [file 41467_2022_29644_MOESM1_ESM.pdf]

Research Article

Transcription factors AP-2α and AP-2β regulate distinct segments of the distal nephron in the mammalian kidney

Authors:
Joseph O. Lamontagne, Hui Zhang, Alia M Zeid, Karin Strittmatter, Alicia D. Rocha, Trevor Williams, Sheryl Zhang, Alexander G. Marneros

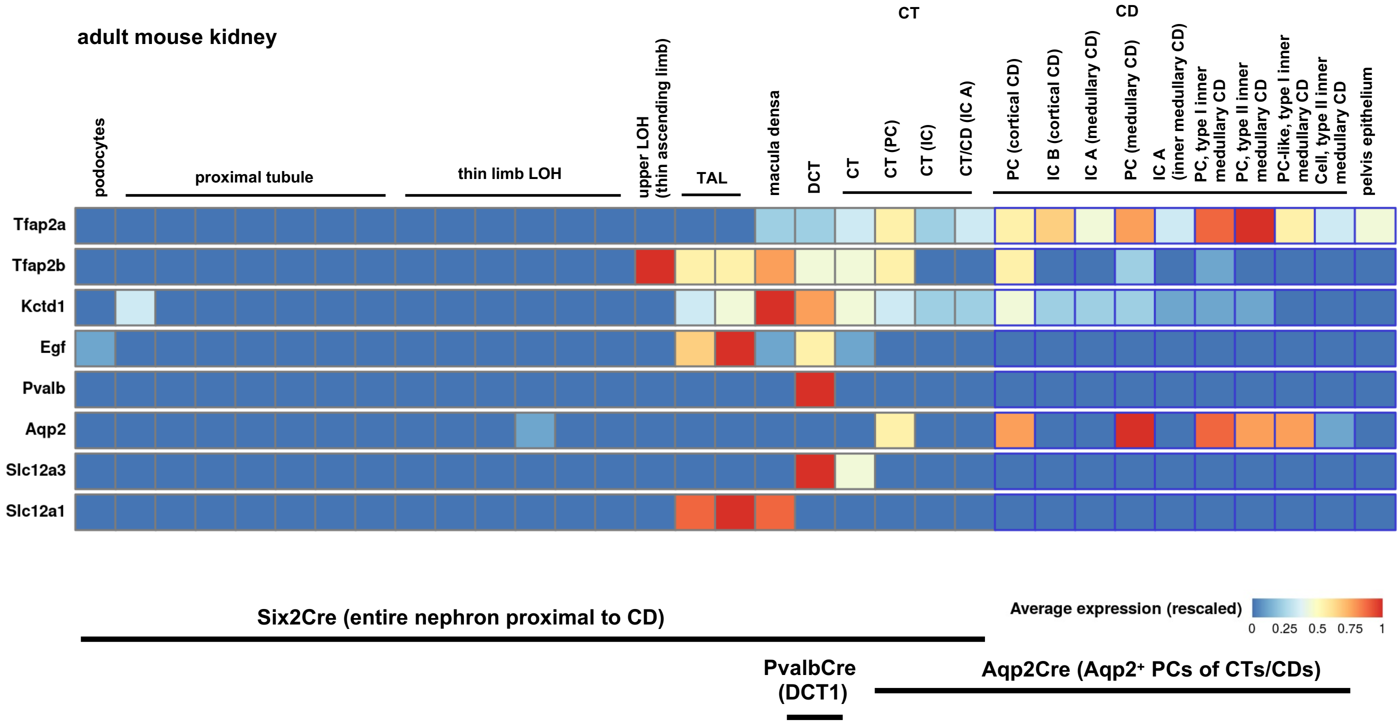

Figure S1: Adult mouse kidney scRNA-Seq data.
Nephron segment-specific expression pattern for AP-2α, AP-2β, KCTD1, EGF, Pvalb, Aqp2, Slc12a3 and Slc12a1 in the adult mouse kidney based on scRNA-Seq data (https://cello.shinyapps.io/kidneycellexplorer/)[1]. Nephron segments targeted by Six2Cre, PvalbCre, and Aqp2Cre mice are shown.

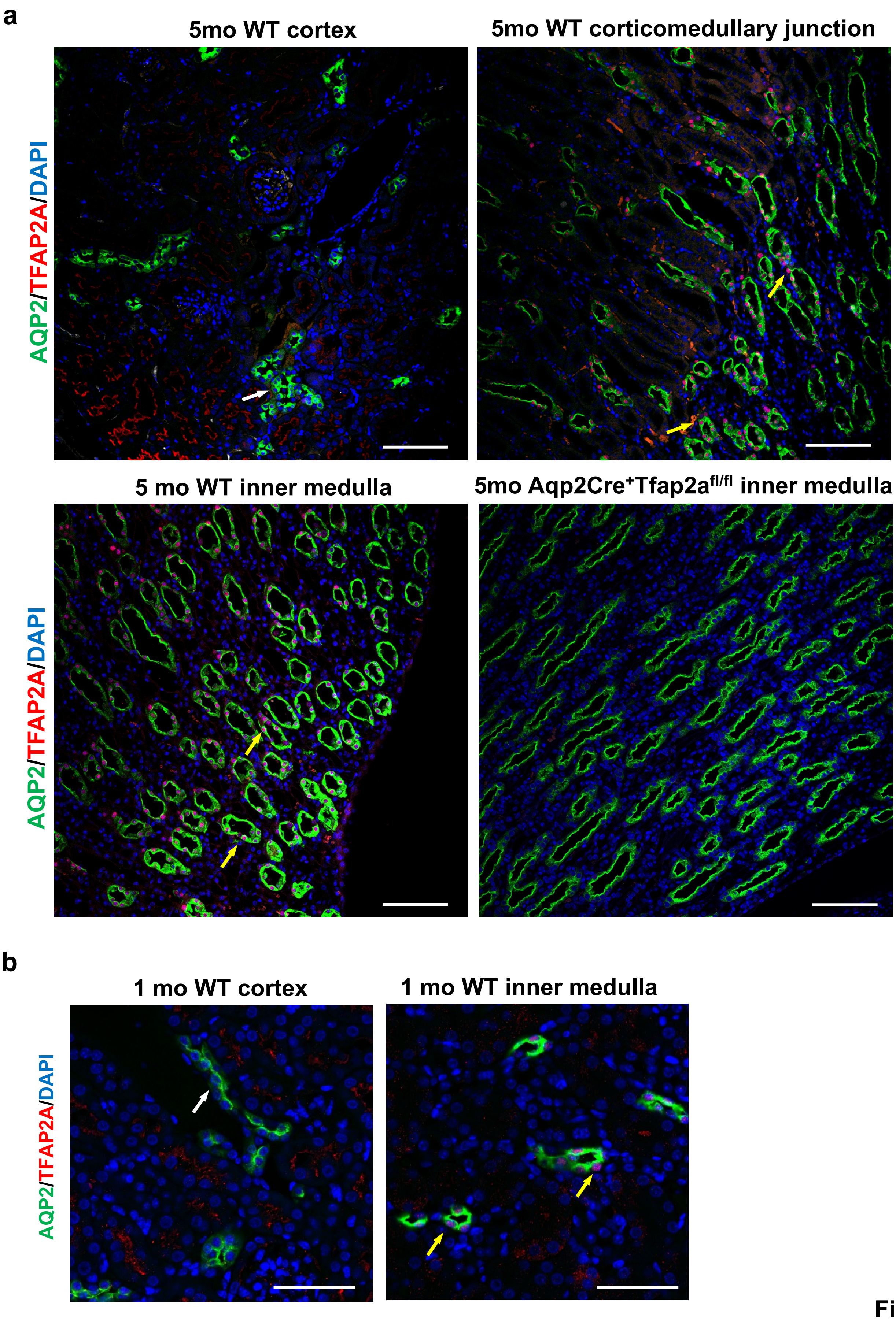
a
5mo WT cortex
5mo WT corticomedullary junction
AQP2/TFAP2A/DAPI
5 mo WT inner medulla
5mo Aqp2Cre+Tfap2afl/fl inner medulla
AQP2/TFAP2A/DAPI
b
1 mo WT cortex
1 mo WT inner medulla
AQP2/TFAP2A/DAPI

Figure S2

Figure S2: AP-2α protein shows nuclear localization in medullary CDs.
Immunolabeling for AP-2α shows nuclear localization in medullary $Aqp2^+$ CDs (yellow arrows) but not in cortical $Aqp2^+$ CDs or CTs (white arrows) in 5-months-old (A) or 1-months-old mice (B). $Aqp2Cre^+Tfap2a^{fl/fl}$ mice show no AP-2α immunolabeling in medullary CDs. Scale bars, 100μm.

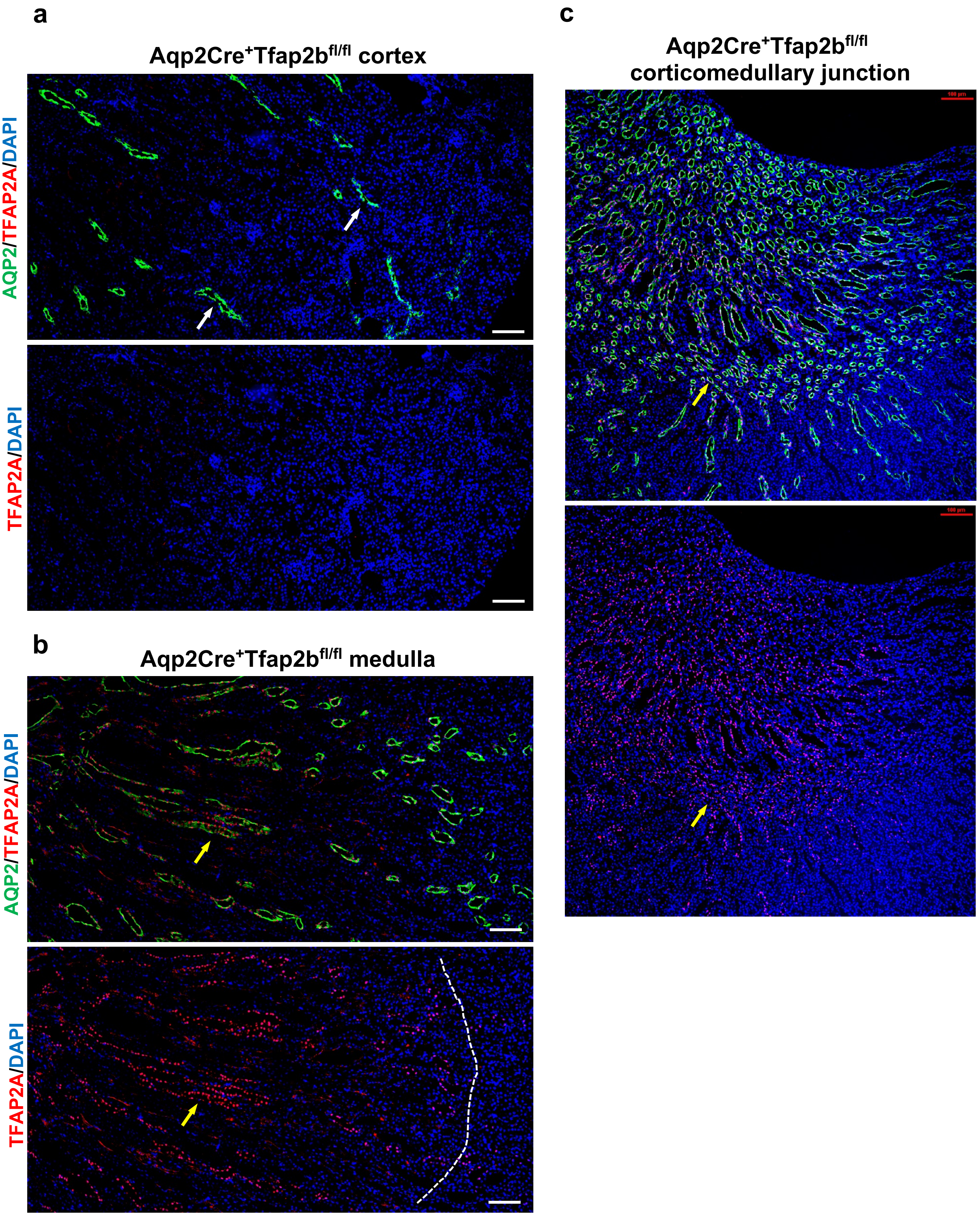
a
Aqp2Cre+Tfap2bfl/fl cortex
AQP2/TFAP2A/DAPI
TFAP2A/DAPI
b
Aqp2Cre+Tfap2bfl/fl medulla
AQP2/TFAP2A/DAPI
TFAP2A/DAPI
c
Aqp2Cre+Tfap2bfl/fl
corticomedullary junction

Figure S3

Figure S3: AP-2α protein shows nuclear localization in medullary CDs independently of AP-2β expression.

Immunolabeling for AP-2α in kidneys of Aqp2Cre^{+}Tfap2b$^{fl/fl}$ mice. No immunolabeling for AP-2α in the cortex (white arrows) is observed (A), whereas medullary CDs (yellow arrows) show strong nuclear labeling for AP-2α (B and C). Bottom images are the same images shown on top without the green channel (Aqp2^{+}). Dotted line in (B) indicates corticomedullary junction. 13-months-old mouse. Scale bars, 100μm.

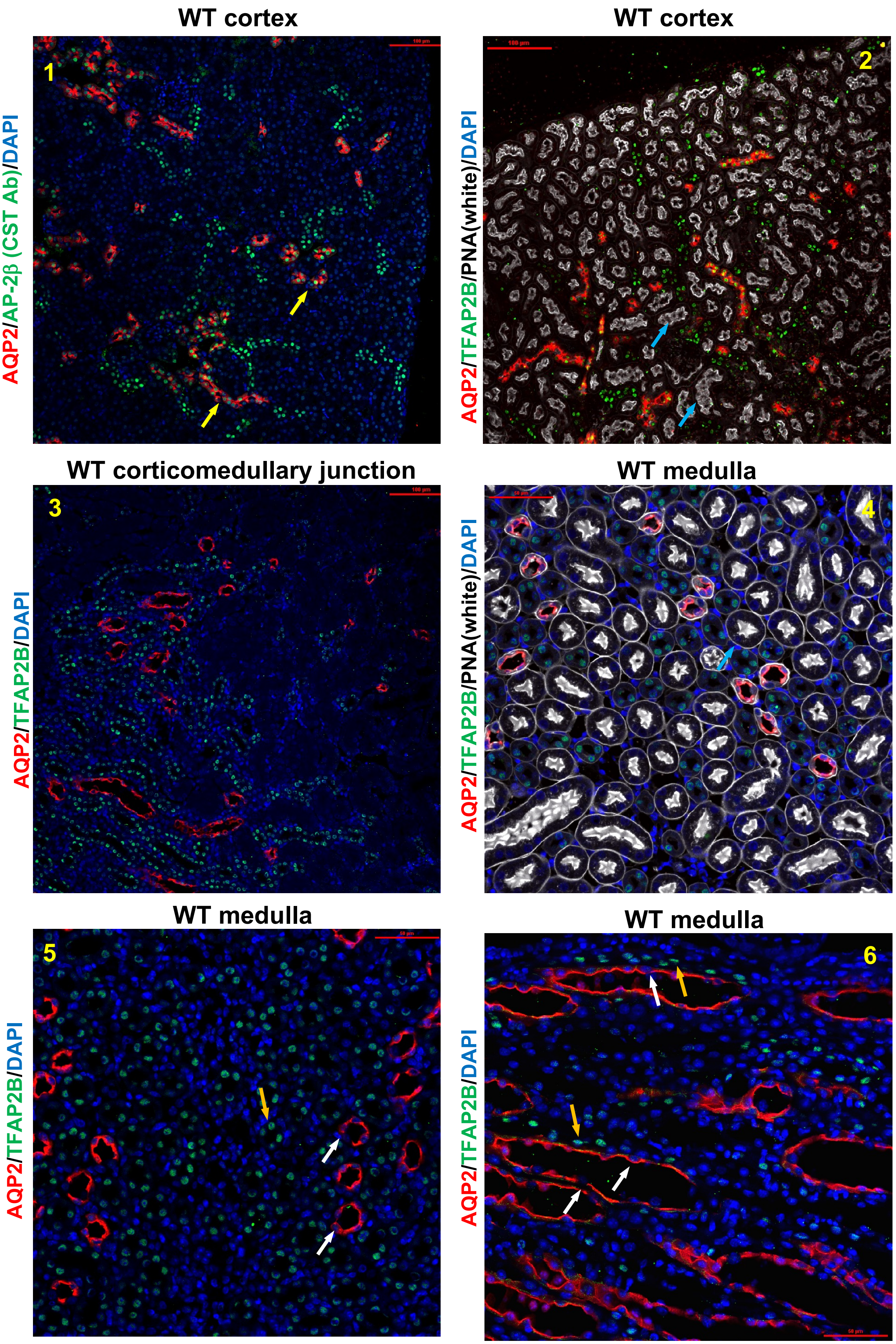
WT cortex
1
AQP2/AP-2β (CST Ab)/DAPI
WT cortex
2
AQP2/TFAP2B/PNA(white)/DAPI
WT corticomedullary junction
3
AQP2/TFAP2B/DAPI
WT medulla
4
AQP2/TFAP2B/PNA(white)/DAPI
WT medulla
5
AQP2/TFAP2B/DAPI
WT medulla
6
AQP2/TFAP2B/DAPI

Figure S4

Figure S4: AP-2β protein immunolocalization in the adult mouse kidney.

Confocal microscopy images from the cortex, corticomedullary junction, and medulla of kidneys from adult WT mice immunolabeled for AP-2β using the anti-AP-2β antibody from Cell Signaling Technology. AP-2β is found in cortical CDs/CTs (Aqp2$^+$) (yellow arrows) and other cortical distal nephron segments, while no AP-2β is detected in PTs (highlighted by PNA; blue arrows). In the medulla, CDs show no AP-2β immunolabeling (white arrows), but adjacent non-CD distal nephron segments do (orange arrows). 1-month-old WT mouse. Scale bars, 100µm (1,2,3) and 50µm (4,5,6).

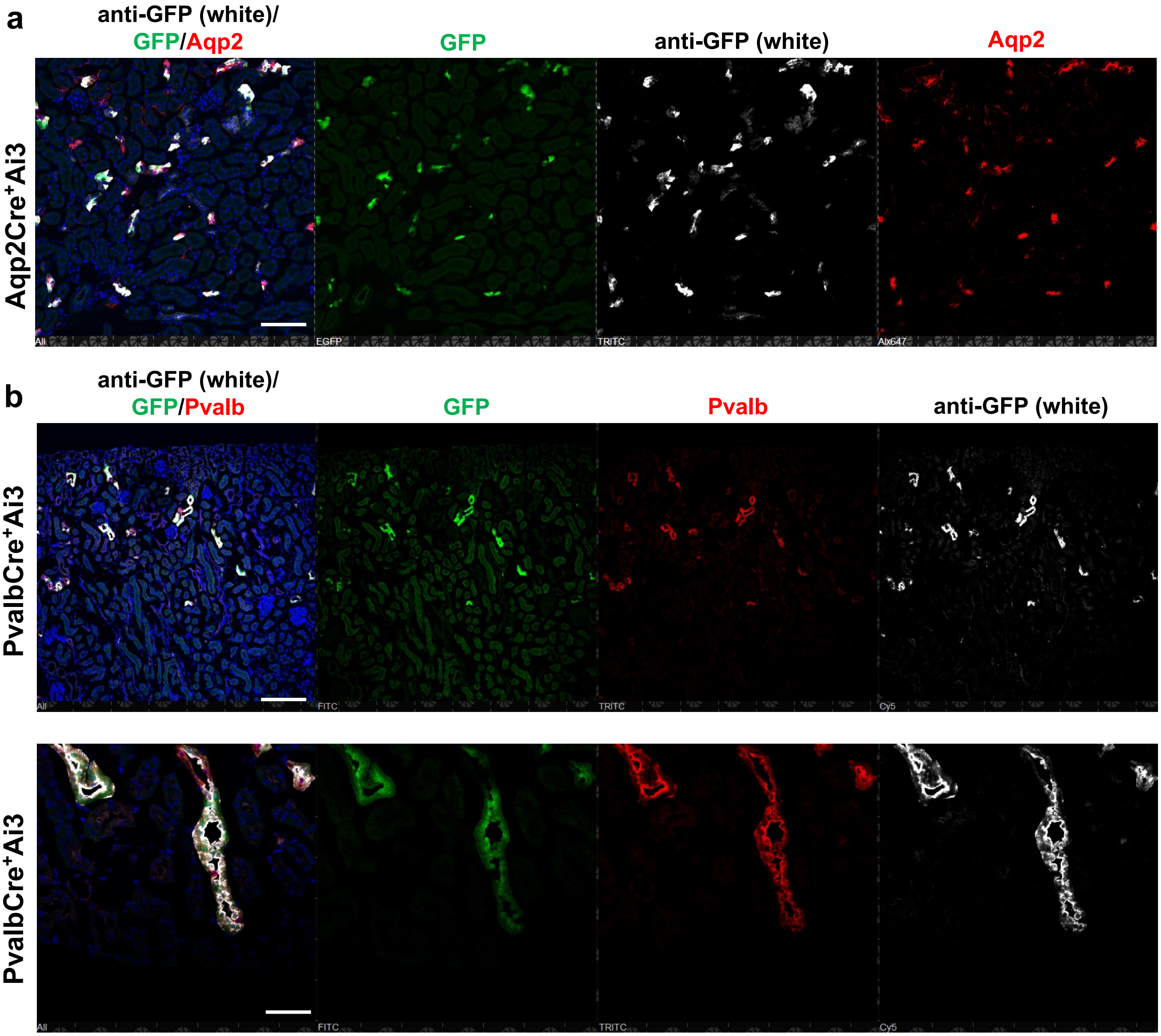

Figure S5: Aqp2Cre mice induce Cre-mediated removal of floxed alleles in Aqp2⁺ nephron segments (CTs/CDs), whereas PvalbCre mice induce Cre-mediated removal of floxed alleles in Pvalb⁺ nephron segments (DCT1s).

A. Aqp2Cre⁺ mice crossed with B6.Cg-Gt(ROSA)26Sor$^{tm3(CAG-EYFP)Hze}$/J (Ai3) reporter mice show that Cre-mediated removal of floxed alleles occurs in Aqp2⁺ nephron segments (CTs/CDs). Co-immunolabeling for Aqp2 (red) and GFP (anti-GFP antibody labeling; white). Intrinsic GFP signal is shown in green. Scale bar, 100µm.

B. PvalbCre⁺ mice crossed with B6.Cg-Gt(ROSA)26Sor$^{tm3(CAG-EYFP)Hze}$/J (Ai3) reporter mice show that Cre-mediated removal of floxed alleles occurs in Pvalb⁺ nephron segments. Co-immunolabeling for Pvalb (red) and GFP (anti-GFP antibody labeling; white). Intrinsic GFP signal is shown in green. Scale bars, 100µm (top), 50µm (bottom).

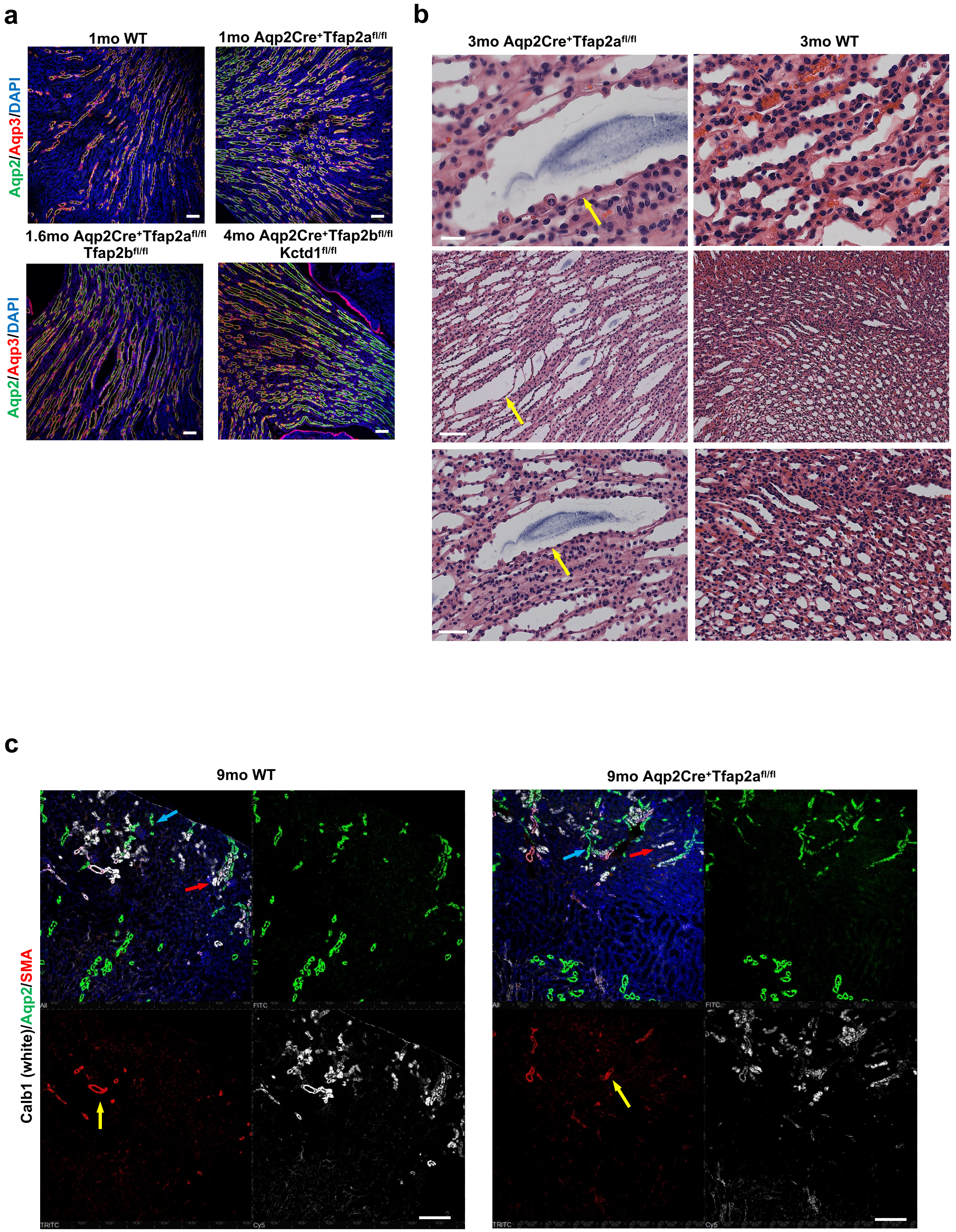

a
1mo WT
1mo Aqp2Cre+Tfap2afl/fl
Aqp2/Aqp3/DAPI
1.6mo Aqp2Cre+Tfap2afl/fl Tfap2bfl/fl
4mo Aqp2Cre+Tfap2bfl/fl Kctd1fl/fl
Aqp2/Aqp3/DAPI
b
3mo Aqp2Cre+Tfap2afl/fl
3mo WT
c
9mo WT
9mo Aqp2Cre+Tfap2afl/fl
Calb1 (white)/Aqp2/SMA
All
FITC
TRITC
Cy5
All
FITC
TRITC
Cy5

Figure S6

Figure S6: Dilatation of medullary CDs in Aqp2Cre^{+}Tfap2a$^{fl/fl}$ mice.

A. Immunolabeling for CD markers Aqp2 and Aqp3 shows normal appearance of medullary CDs in 1-months-old Aqp2Cre^{+}Tfap2a$^{fl/fl}$ mice, 1.6 months-old Aqp2Cre^{+}Tfap2a$^{fl/fl}$Tfap2b$^{fl/fl}$ mice, or 4-months-old Aqp2Cre^{+}Tfap2b$^{fl/fl}$KCTD1$^{fl/fl}$ mice. Scale bars, 100µm.

B. 3-months-old Aqp2Cre^{+}Tfap2a$^{fl/fl}$ mice show dilatation of medullary CDs with flattened epithelium (yellow arrows) compared to WT littermates. H&E images. Scale bars: 20µm (top), 100µm (middle), 50µm (bottom).

C. Cortical CDs (Aqp2^{+}; blue arrows) and CTs (Calb1^{+}Aqp2^{+}; red arrows) appear normal in 9-months-old Aqp2Cre^{+}Tfap2a$^{fl/fl}$ mice, as seen in WT littermates. SMA labeling (yellow arrows) identifies pericytes of vessels in the kidney but no tubulointerstitial fibrosis is observed (mouse IgG deposits in vessel walls cause signal for the secondary anti-mouse antibody used to detect Calb1). Scale bars, 200µm.

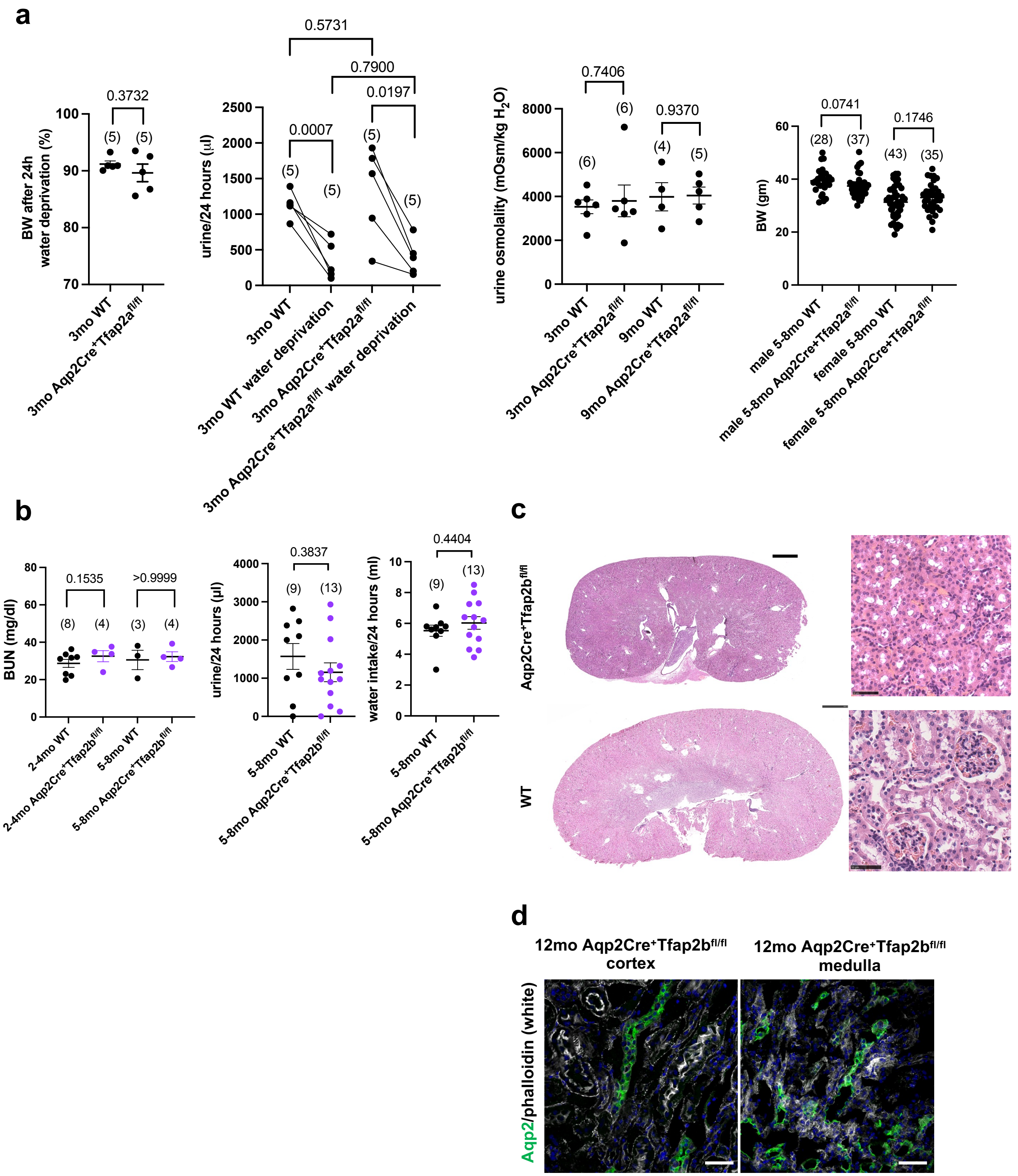

a
0.3732
BW after 24h water deprivation (%)
3mo WT
3mo Aqp2Cre+Tfap2a fl/fl
0.5731
0.7900
0.0197
0.0007
urine/24 hours (µl)
3mo WT water deprivation
3mo Aqp2Cre+Tfap2a fl/fl water deprivation
0.7406
0.9370
urine osmolality (mOsm/kg H2O)
9mo WT
9mo Aqp2Cre+Tfap2a fl/fl
0.0741
0.1746
BW (gm)
male 5-8mo WT
male 5-8mo Aqp2Cre+Tfap2a fl/fl
female 5-8mo WT
female 5-8mo Aqp2Cre+Tfap2a fl/fl
b
0.1535
>0.9999
BUN (mg/dl)
2-4mo WT
2-4mo Aqp2Cre+Tfap2b fl/fl
5-8mo WT
5-8mo Aqp2Cre+Tfap2b fl/fl
0.3837
urine/24 hours (µl)
0.4404
water intake/24 hours (ml)
c
Aqp2Cre+Tfap2b fl/fl
WT
d
12mo Aqp2Cre+Tfap2b fl/fl cortex
12mo Aqp2Cre+Tfap2b fl/fl medulla
Aqp2/phalloidin (white)

Figure S7

Figure S7: Characterization of Aqp2Cre^{+}Tfap2a$^{fl/fl}$ mice and Aqp2Cre^{+}Tfap2b$^{fl/fl}$ mice.

A. Left: Reduction in body weight (BW) after 24-hour water deprivation is not significantly different between 3 months-old Aqp2Cre^{+}Tfap2a$^{fl/fl}$ mice compared to WT littermates. Left middle: No significant difference is observed in 24-hour urine production in the presence or absence of water (water deprivation for 24 hours). Right middle: No difference in urine osmolality is observed between 3-months-old or 9-months-old Aqp2Cre^{+}Tfap2a$^{fl/fl}$ mice and WT littermates. Right: No difference in BW in 5-8-months-old Aqp2Cre^{+}Tfap2a$^{fl/fl}$ mice compared to WT littermates irrespective of gender. P-values are shown (two-tailed t-test). Graphs represent data as mean ± SEM. Number of mice per group is indicated in brackets. Source data are provided as a Source Data File.

B. No significant differences in BUN levels or 24-hour urine production or water intake in 5-8-months-old Aqp2Cre^{+}Tfap2b$^{fl/fl}$ mice. P-values are shown (two-tailed Mann Whitney test). Graphs represent data as mean ± SEM. Number of mice per group is indicated in brackets. Source data are provided as a Source Data File.

C. Normal histology of 10-12-months-old Aqp2Cre^{+}Tfap2b$^{fl/fl}$ mice compared to WT littermate mice. H&E sections (left: whole kidney; right: high magnification images of the same kidney) Scale bars, 1mm left and 50µm right.

D. Immunolabeling for Aqp2 shows normal morphology of CTs/CDs in 12-months-old Aqp2Cre^{+}Tfap2b$^{fl/fl}$ mice. Scale bars, 50µm.

9mo: WT and Aqp2Cre$^+$Tfap2a$^{fl/fl}$

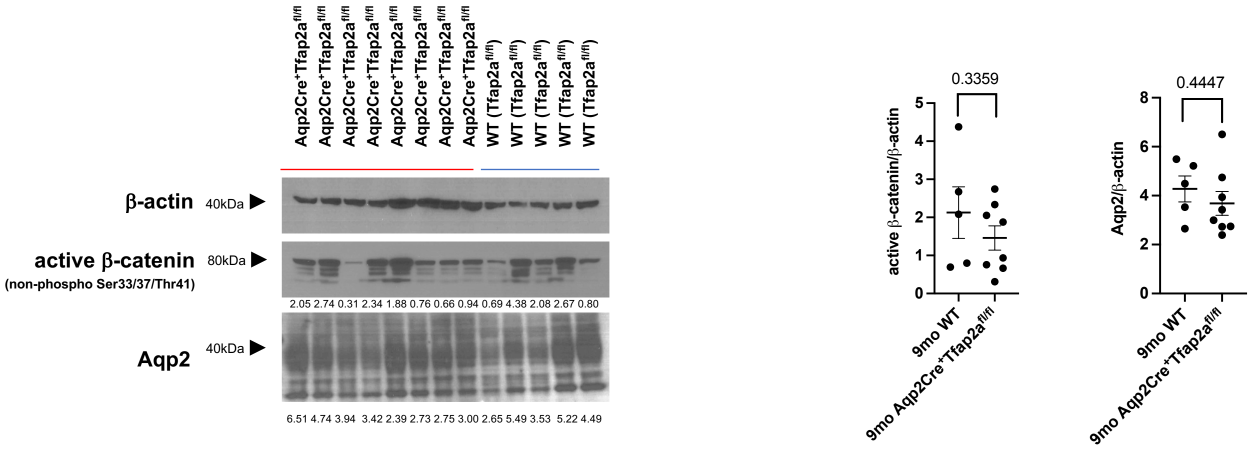

6mo: WT and Aqp2Cre$^+$Tfap2a$^{fl/fl}$

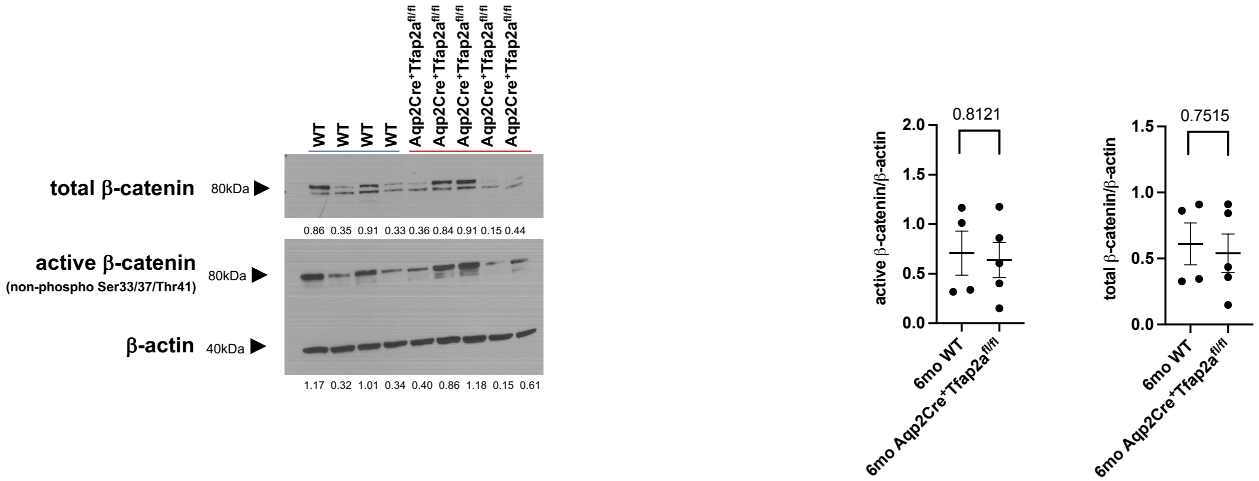

1-2mo: WT and Aqp2Cre$^+$Tfap2a$^{fl/fl}$

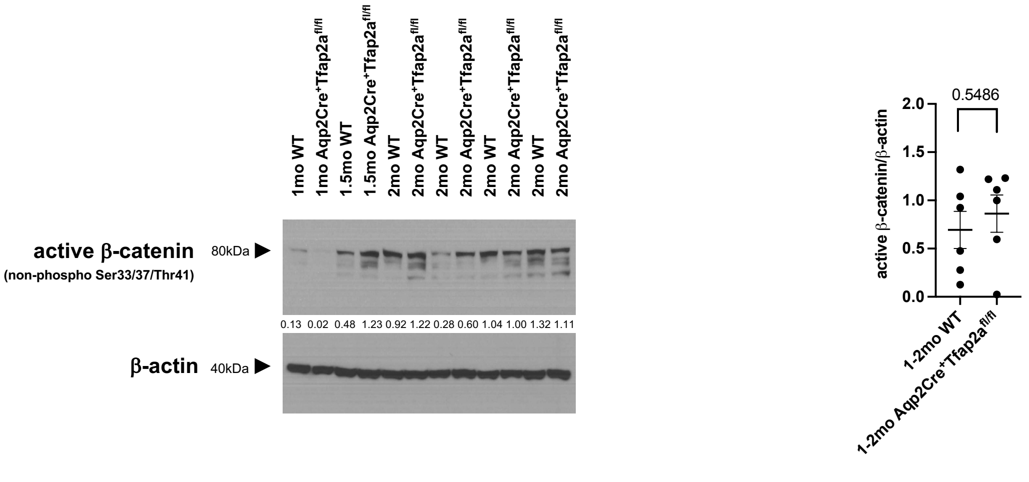

Figure S8

Figure S8: Western blotting of whole kidney lysates of age-matched WT and Aqp2Cre^{+}Tfap2a$^{fl/fl}$ mice in different age groups.

Whole kidney lysates of 1-2-months-old (n=6/group), 6-months-old (n=4 WT and 5 Aqp2Cre^{+}Tfap2a$^{fl/fl}$ mice), and 9-months-old (n=5 WT and 8 Aqp2Cre^{+}Tfap2a$^{fl/fl}$ mice) Aqp2Cre^{+}Tfap2a$^{fl/fl}$ mice and WT littermate mice were used for Western blotting experiments. Variability in total β-catenin and active β-catenin (non-phospho Ser33/37/Thr41 β-catenin) was observed without a statistically significant consistent difference between mutant and control groups. Renal Aqp2 protein levels are not diminished in Aqp2Cre^{+}Tfap2a$^{fl/fl}$ mice. β-actin loading control. Size markers and densitometric values for Western blot bands normalized to β-actin are shown. P-values were determined by a two-tailed unpaired t-test. Graphs represent data as mean ± SEM. Source data are provided as a Source Data File.

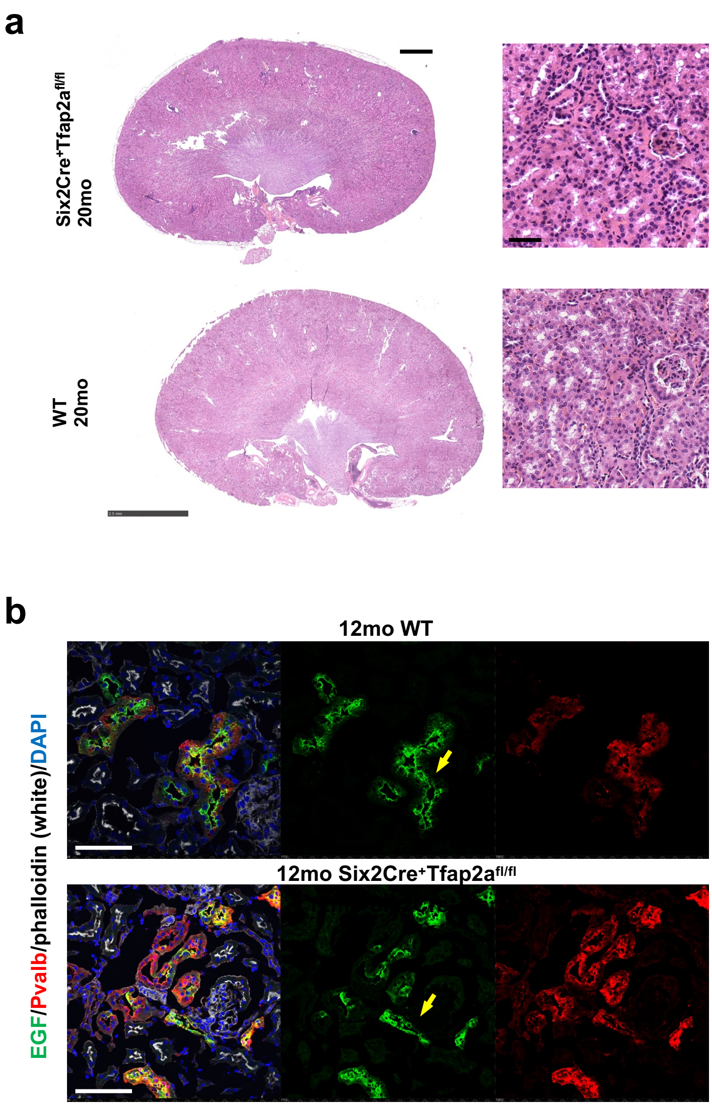

Figure S9: Normal renal morphology observed in kidneys from Six2Cre$^+$Tfap2a$^{fl/fl}$ mice.

A. H&E sections (left: whole kidney; right: high magnification images of the same kidney) show that Six2Cre$^+$Tfap2a$^{fl/fl}$ mice have normal kidney histology even at an advanced age. 20-months-old mice. Scale bars, 1mm left and 50µm right.

B. Even aged (12-months-old) Six2Cre$^+$Tfap2a$^{fl/fl}$ mice show normal DCT1 morphology (Pvalb$^+$) and proper EGF expression in DCTs (yellow arrows). Scale bars, 100µm.

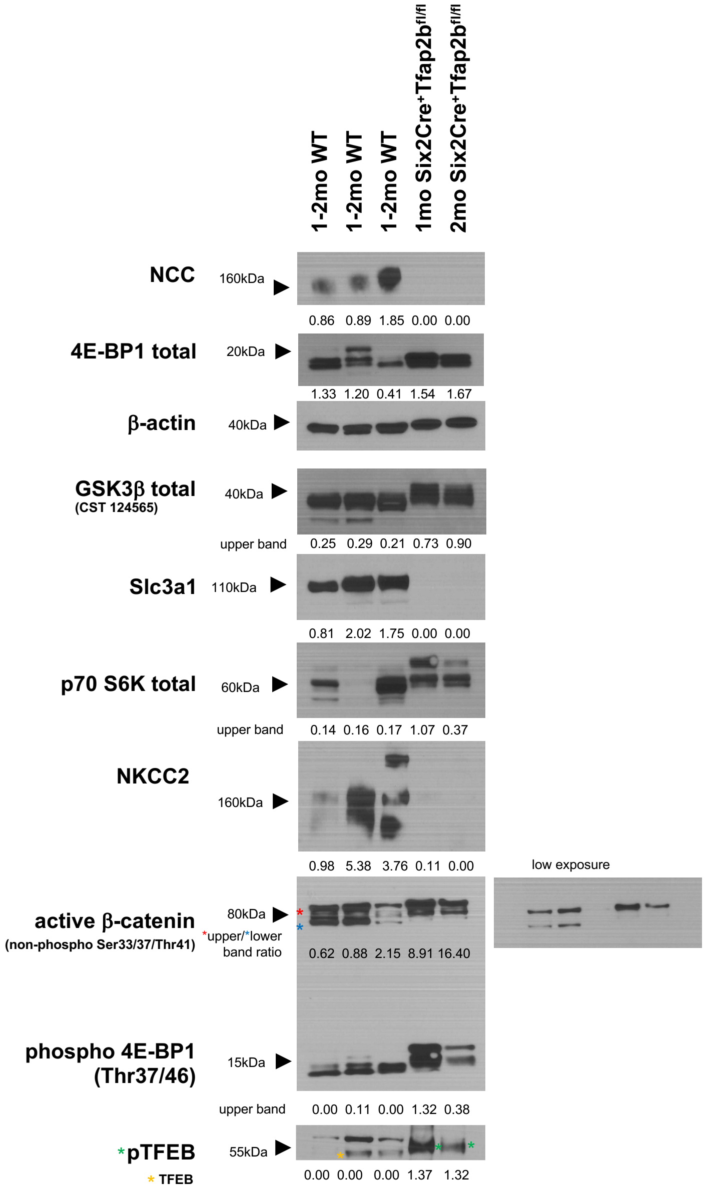

Figure S10: Western blotting experiments with kidneys of Six2Cre⁺Tfap2b$^{fl/fl}$ mice.
Whole kidney lysates of 1-2 months-old Six2Cre⁺Tfap2b$^{fl/fl}$ mice (n=2; as seen in Figure 4d) and WT controls (n=3) were used for Western blotting experiments. β-actin as a loading control. Densitometric values for Western blot bands normalized to β-actin are shown. Size markers are indicated by arrowheads. The anti-active β-catenin (non-phospho Ser33/37/Thr41) antibody detects a shift towards an upper band in the kidney samples of Six2Cre⁺Tfap2b$^{fl/fl}$ mice (*: upper band; *: lower band). pTFEB indicated by *, TFEB by *.

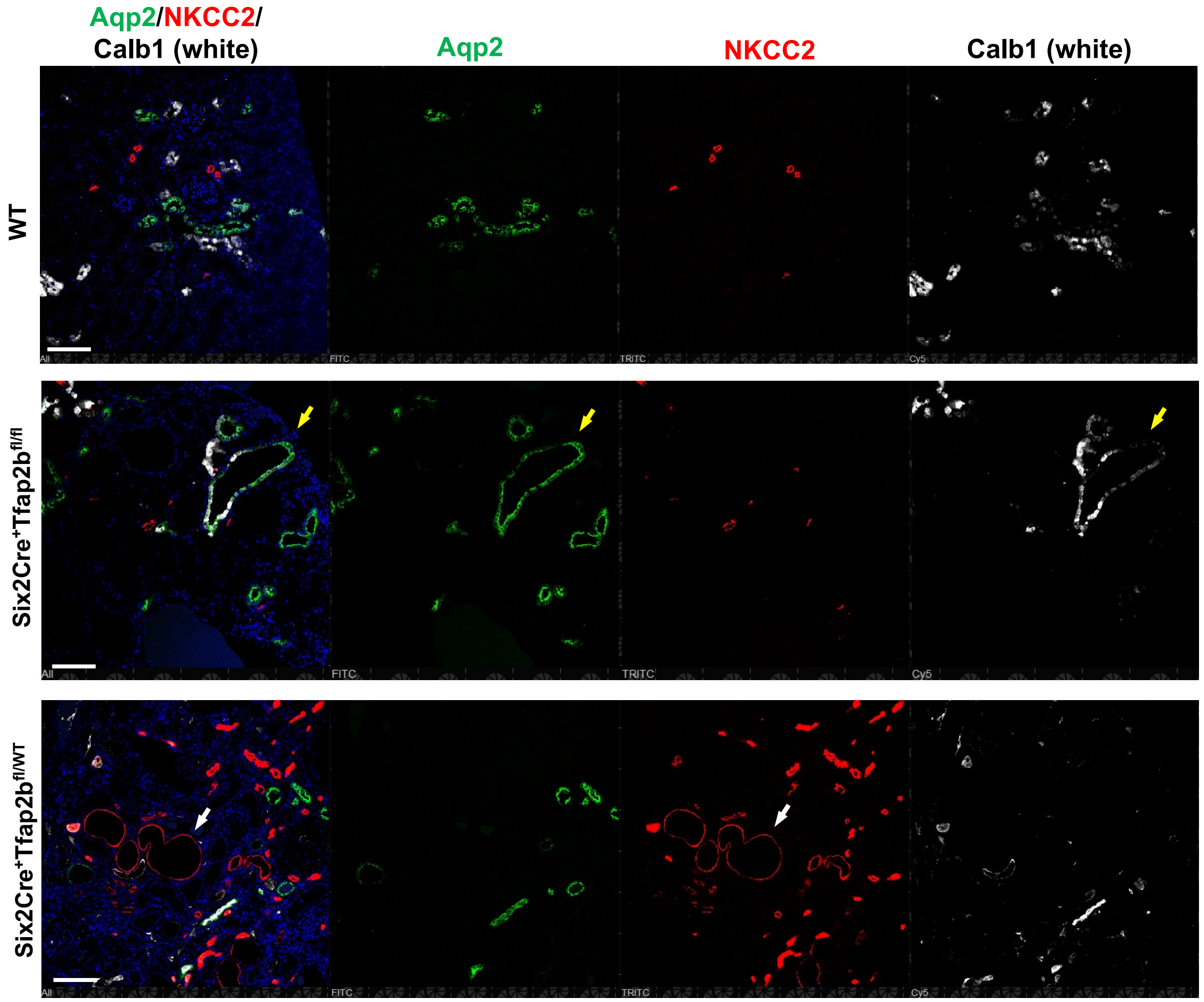

Figure S11: Immunolabeling for Aqp2, NKCC2, and Calb1 in kidneys from Six2Cre$^+$Tfap2b$^{fl/fl}$ mice, Six2Cre$^+$Tfap2b$^{fl/WT}$ mice, and WT controls.

Dilatation of CTs (Calb1$^+$Aqp2$^+$) is observed in 1-months old Six2Cre$^+$Tfap2b$^{fl/fl}$ mice (yellow arrows). Aged Six2Cre$^+$Tfap2b$^{fl/WT}$ mice (12-months-old) show TAL (NKCC2$^+$) dilatation (white arrows). Scale bars, 100µm.

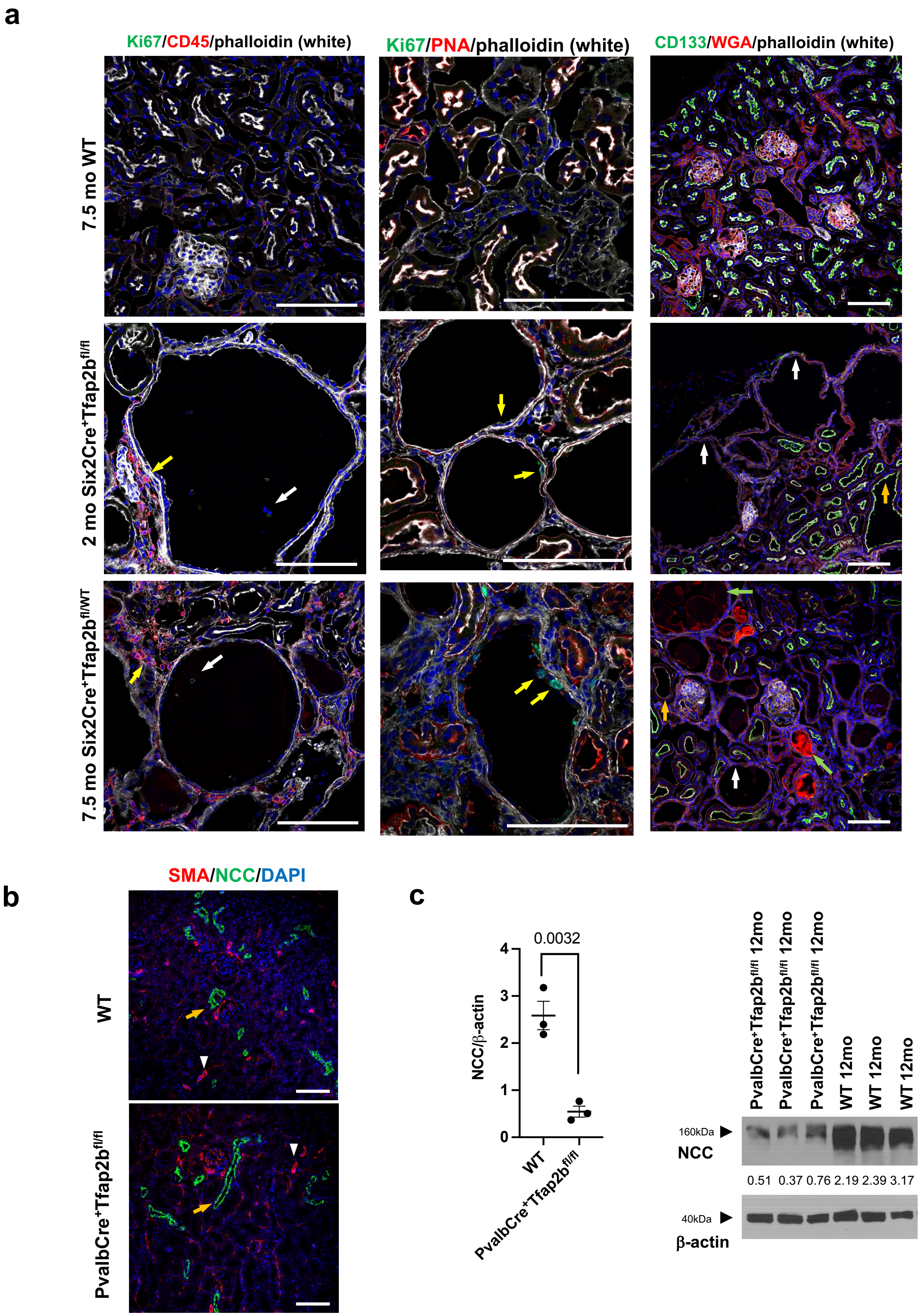
a
Ki67/CD45/phalloidin (white)
Ki67/PNA/phalloidin (white)
CD133/WGA/phalloidin (white)
7.5 mo WT
2 mo Six2Cre+Tfap2bfl/fl
7.5 mo Six2Cre+Tfap2bfl/WT
b
SMA/NCC/DAPI
WT
PvalbCre+Tfap2bfl/fl
c
0.0032
NCC/β-actin
4
3
2
1
0
WT
PvalbCre+Tfap2bfl/fl
PvalbCre+Tfap2bfl/fl 12mo
PvalbCre+Tfap2bfl/fl 12mo
PvalbCre+Tfap2bfl/fl 12mo
WT 12mo
WT 12mo
WT 12mo
160kDa
NCC
0.51 0.37 0.76 2.19 2.39 3.17
40kDa
β-actin

Figure S12

Figure S12: Immunolabeling for Ki67, CD45, and CD133 with kidneys from a 2-months-old Six2Cre^{+}Tfap2b$^{fl/fl}$ mouse, a 7.5-months-old Six2Cre^{+}Tfap2b$^{fl/WT}$ mouse, and a 7.5-months-old Cre-negative control mouse.

A. Left: Kidneys of Six2Cre^{+}Tfap2b$^{fl/fl}$ mice and Six2Cre^{+}Tfap2b$^{fl/WT}$ mice show an extensive leukocytic (CD45^{+}) interstitial inflammatory infiltrate (yellow arrows). These mice show cortical cysts with detached epithelial cells in the lumen (CD45^{-}) (white arrows). Middle: Immunolabeling for the proliferation marker Ki67 is observed in some epithelial cells of dilated tubules/cysts (yellow arrows) in kidneys of both Six2Cre^{+}Tfap2b$^{fl/fl}$ mice and Six2Cre^{+}Tfap2b$^{fl/WT}$ mice. Right: Labeling of these kidneys with the lectin WGA (demarcates distal nephron tubules and glomeruli) and phalloidin (identifies PTs), as well as for CD133 (marks Bowman's capsule of glomeruli and PTs). Cortical cysts in Six2Cre^{+}Tfap2b$^{fl/fl}$ mice and Six2Cre^{+}Tfap2b$^{fl/WT}$ mice are not PTs (CD133^{-}, no phalloidin^{+} brush membrane) (white arrows), albeit some PTs show extensive dilatation (yellow arrow). Protein casts are observed in dilated tubules (green arrows). Scale bars, 100µm.

B. Immunolabeling shows that DCTs in PvalbCre^{+}Tfap2b$^{fl/fl}$ mice still express NCC (orange arrows). SMA labeling is detected in pericytes of vessels (white arrowheads) but no interstitial fibrosis is observed. 16-months-old mice. Scale bars, 100µm.

C. Reduced NCC protein levels in whole kidney lysates of 12-months-old PvalbCre^{+}Tfap2b$^{fl/fl}$ mice compared to WT littermates (n=3 mice/group). Densitometric values for Western blot bands normalized to β-actin are shown. Size markers are indicated. P-value was determined by a two-tailed t-test. Graph represents data as mean $\pm$ SEM. Source data are provided as a Source Data File.

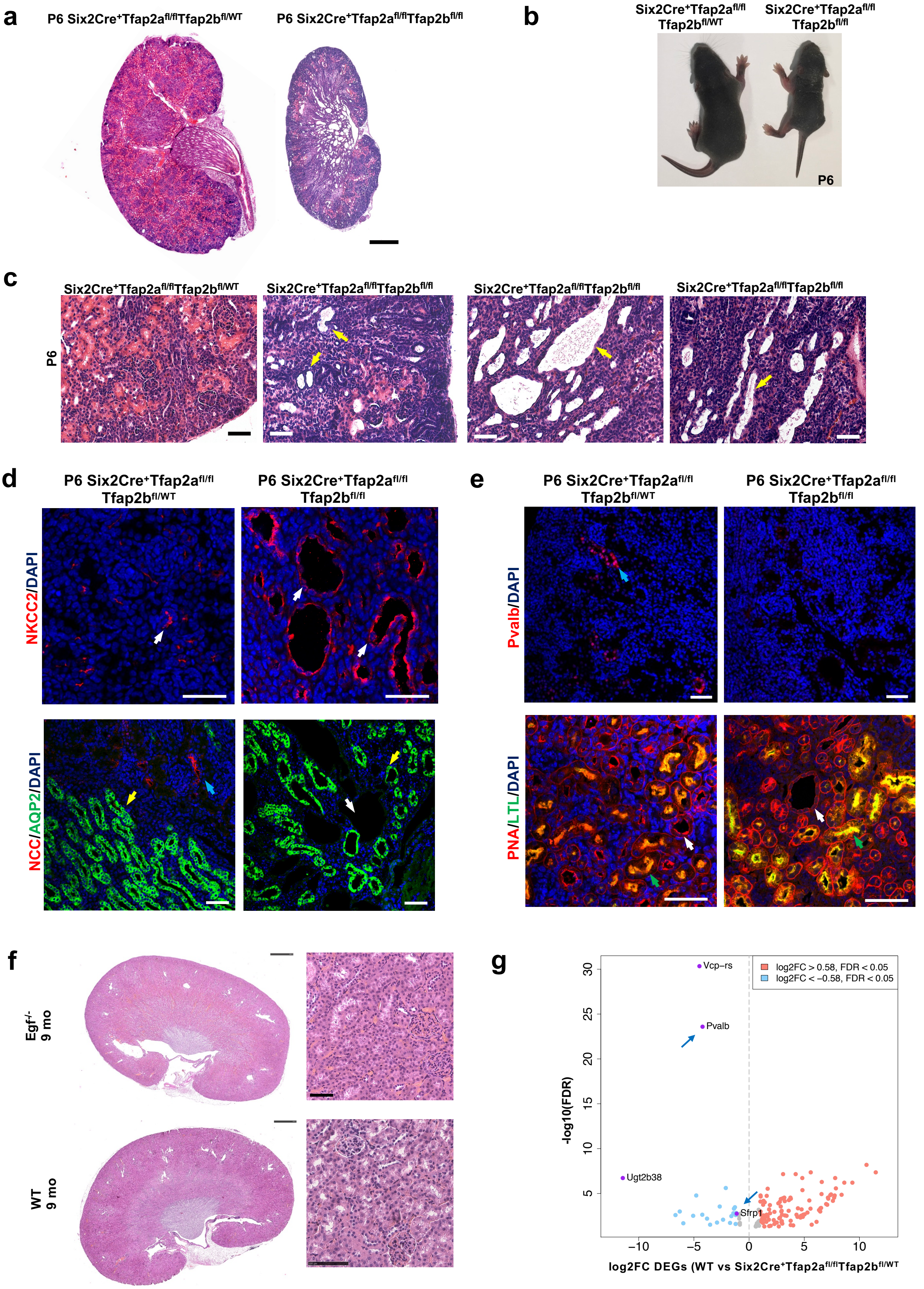
a
P6 Six2Cre+Tfap2afl/flTfap2bfl/WT
P6 Six2Cre+Tfap2afl/flTfap2bfl/fl
b
Six2Cre+Tfap2afl/fl Tfap2bfl/WT
Six2Cre+Tfap2afl/fl Tfap2bfl/fl
P6
c
P6
Six2Cre+Tfap2afl/flTfap2bfl/WT
Six2Cre+Tfap2afl/flTfap2bfl/fl
Six2Cre+Tfap2afl/flTfap2bfl/fl
Six2Cre+Tfap2afl/flTfap2bfl/fl
d
P6 Six2Cre+Tfap2afl/fl Tfap2bfl/WT
P6 Six2Cre+Tfap2afl/fl Tfap2bfl/fl
NKCC2/DAPI
NCC/AQP2/DAPI
e
P6 Six2Cre+Tfap2afl/fl Tfap2bfl/WT
P6 Six2Cre+Tfap2afl/fl Tfap2bfl/fl
Pvalb/DAPI
PNA/LTL/DAPI
f
Egf-/- 9 mo
WT 9 mo
g
Vcp−rs
Pvalb
Ugt2b38
Sfrp1
log2FC > 0.58, FDR < 0.05
log2FC < −0.58, FDR < 0.05
-log10(FDR)
log2FC DEGs (WT vs Six2Cre+Tfap2afl/flTfap2bfl/WT
−10
−5
0
5
10
5
10
15
20
25
30

Figure S13

Figure S13: Combined inactivation of AP-2α and AP-2β in Six2^{+} NPCs.

A. Reduced kidney size with distal nephron dilatation is seen in a P6 Six2Cre^{+}Tfap2a$^{fl/fl}$Tfap2b$^{fl/fl}$ mouse, whereas its Six2Cre^{+}Tfap2a$^{fl/fl}$Tfap2b$^{fl/WT}$ littermate does not show these defects. H&E staining. Scale bar, 500 μm.

B. Severe postnatal growth retardation in mice lacking AP-2β in the nephron proximal to the CDs, shown here in a direct comparison of a P6 Six2Cre^{+}Tfap2a$^{fl/fl}$Tfap2b$^{fl/WT}$ mouse with a Six2Cre^{+}Tfap2a$^{fl/fl}$Tfap2b$^{fl/fl}$ littermate. Images C.-E. are derived from these two mice.

C. Higher magnification images of these kidneys (from A.) show cystic dilatation of distal nephron epithelia (yellow arrows) in P6 Six2Cre^{+}Tfap2a$^{fl/fl}$Tfap2b$^{fl/fl}$ kidneys but not in kidneys from P6 Six2Cre^{+}Tfap2a$^{fl/fl}$Tfap2b$^{fl/WT}$ mice. Scale bars, 50 μm.

D.-E. Immunofluorescent labelings show that the dilatation of distal nephron segments in kidneys from P6 Six2Cre^{+}Tfap2a$^{fl/fl}$Tfap2b$^{fl/fl}$ mice occurs in TALs (NKCC2^{+}) (white arrows), but at that age to a lesser extent in CTs/CDs (AQP2^{+}; yellow arrows) and not in PTs (LTL^{+}; green arrows). In Six2Cre^{+}Tfap2a$^{fl/fl}$Tfap2b$^{fl/fl}$ mice DCTs (Pvalb^{+}NCC^{+}) are missing (seen in Six2Cre^{+}Tfap2a$^{fl/fl}$Tfap2b$^{fl/WT}$ mice; blue arrows). Scale bars, 50 μm.

F. H&E sections (left: whole kidney; right: high magnification images of the same kidney) show a kidney from a 9-months-old Egf$^{-/-}$ mouse with normal kidney histology. Scale bars, 1mm left and 50μm right.

G. Venn diagram of RNA-Seq data of whole kidney lysates from 2-months-old Six2Cre^{+}Tfap2a$^{fl/fl}$Tfap2b$^{fl/WT}$ mice compared to Cre-negative control littermates (n=3 mice/group) show reduced expression of genes that are normally highly expressed in DCTs, such as Pvalb and SFRP1 (arrows). DEGs: differentially expressed genes.

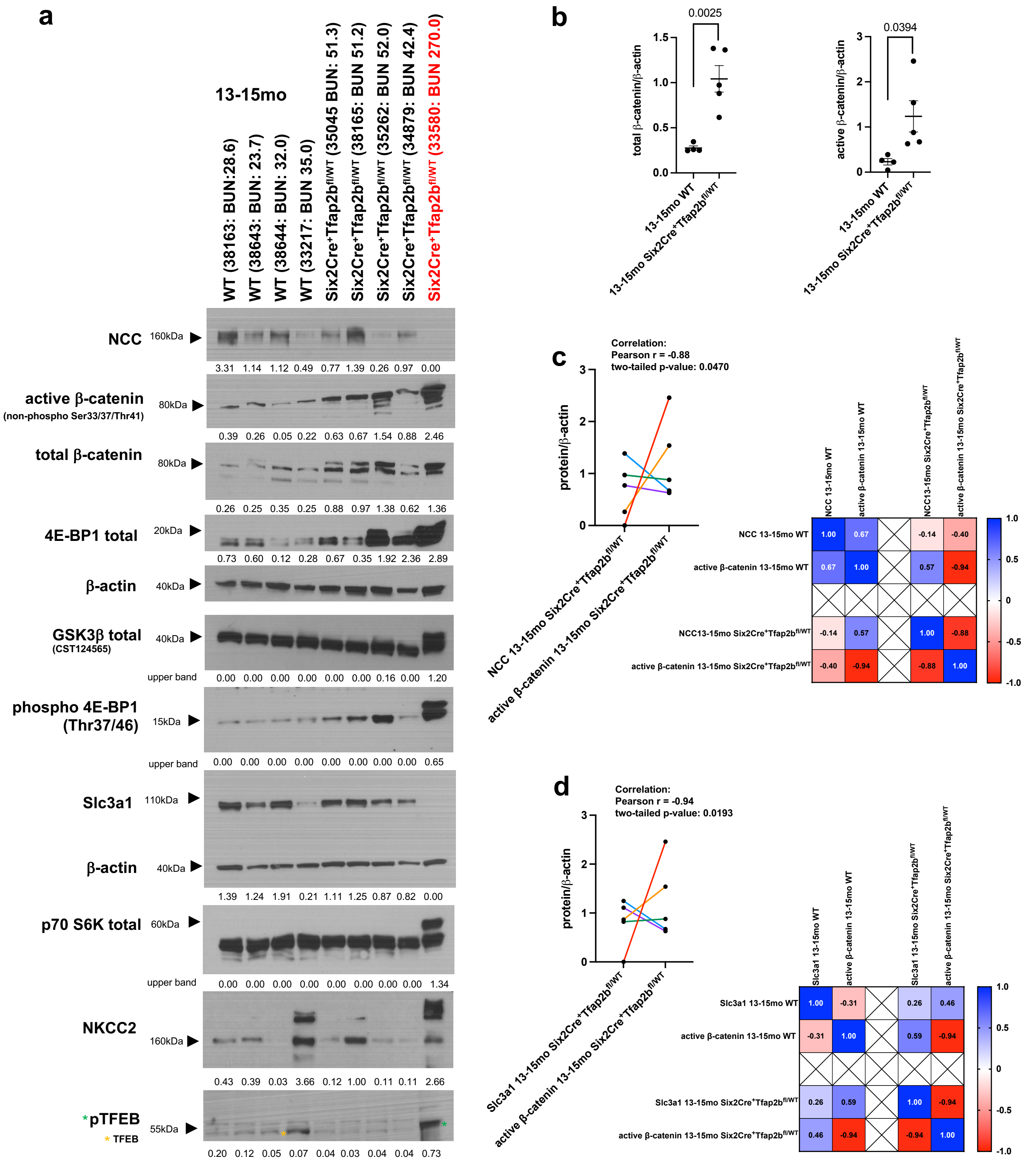
a
13-15mo
WT (38163: BUN:28.6)
WT (38643: BUN: 23.7)
WT (38644: BUN: 32.0)
WT (33217: BUN 35.0)
Six2Cre+Tfap2bfl/WT (35045 BUN: 51.3)
Six2Cre+Tfap2bfl/WT (38165: BUN 51.2)
Six2Cre+Tfap2bfl/WT (35262: BUN 52.0)
Six2Cre+Tfap2bfl/WT (34879: BUN 42.4)
Six2Cre+Tfap2bfl/WT (33580: BUN 270.0)
NCC 160kDa
3.31 1.14 1.12 0.49 0.77 1.39 0.26 0.97 0.00
active β-catenin (non-phospho Ser33/37/Thr41) 80kDa
0.39 0.26 0.05 0.22 0.63 0.67 1.54 0.88 2.46
total β-catenin 80kDa
0.26 0.25 0.35 0.25 0.88 0.97 1.38 0.62 1.36
4E-BP1 total 20kDa
0.73 0.60 0.12 0.28 0.67 0.35 1.92 2.36 2.89
β-actin 40kDa
GSK3β total (CST124565) 40kDa
upper band 0.00 0.00 0.00 0.00 0.00 0.00 0.16 0.00 1.20
phospho 4E-BP1 (Thr37/46) 15kDa
upper band 0.00 0.00 0.00 0.00 0.00 0.00 0.00 0.00 0.65
Slc3a1 110kDa
β-actin 40kDa
1.39 1.24 1.91 0.21 1.11 1.25 0.87 0.82 0.00
p70 S6K total 60kDa
upper band 0.00 0.00 0.00 0.00 0.00 0.00 0.00 0.00 1.34
NKCC2 160kDa
0.43 0.39 0.03 3.66 0.12 1.00 0.11 0.11 2.66
*pTFEB
* TFEB
55kDa
0.20 0.12 0.05 0.07 0.04 0.03 0.04 0.04 0.73
b
0.0025
total β-catenin/β-actin
0.0 0.5 1.0 1.5
13-15mo WT
13-15mo Six2Cre+Tfap2bfl/WT
0.0394
active β-catenin/β-actin
0 1 2 3
13-15mo WT
13-15mo Six2Cre+Tfap2bfl/WT
c
Correlation:
Pearson r = -0.88
two-tailed p-value: 0.0470
protein/β-actin
0 1 2 3
NCC 13-15mo Six2Cre+Tfap2bfl/WT
active β-catenin 13-15mo Six2Cre+Tfap2bfl/WT
NCC 13-15mo WT
active β-catenin 13-15mo WT
NCC13-15mo Six2Cre+Tfap2bfl/WT
active β-catenin 13-15mo Six2Cre+Tfap2bfl/WT
NCC 13-15mo WT 1.00 0.67 -0.14 -0.40
active β-catenin 13-15mo WT 0.67 1.00 0.57 -0.94
NCC13-15mo Six2Cre+Tfap2bfl/WT -0.14 0.57 1.00 -0.88
active β-catenin 13-15mo Six2Cre+Tfap2bfl/WT -0.40 -0.94 -0.88 1.00
1.0 0.5 0 -0.5 -1.0
d
Correlation:
Pearson r = -0.94
two-tailed p-value: 0.0193
protein/β-actin
0 1 2 3
Slc3a1 13-15mo Six2Cre+Tfap2bfl/WT
active β-catenin 13-15mo Six2Cre+Tfap2bfl/WT
Slc3a1 13-15mo WT
active β-catenin 13-15mo WT
Slc3a1 13-15mo Six2Cre+Tfap2bfl/WT
active β-catenin 13-15mo Six2Cre+Tfap2bfl/WT
Slc3a1 13-15mo WT 1.00 -0.31 0.26 0.46
active β-catenin 13-15mo WT -0.31 1.00 0.59 -0.94
Slc3a1 13-15mo Six2Cre+Tfap2bfl/WT 0.26 0.59 1.00 -0.94
active β-catenin 13-15mo Six2Cre+Tfap2bfl/WT 0.46 -0.94 -0.94 1.00
1.0 0.5 0 -0.5 -1.0

Figure S14

Figure S14: Western blotting experiments with kidneys of 13-15-months-old Six2Cre^{+}Tfap2b$^{fl/WT}$ mice.

A. Whole kidney lysates of 13-15-months-old Six2Cre^{+}Tfap2b$^{fl/WT}$ mice (n=5) and age-matched WT controls (n=4) were used for Western blotting experiments, analyzing proteins as shown in Figure 4d. β-actin as a loading control. Densitometric values for Western blot bands normalized to β-actin are shown. Size markers are indicated by arrowheads. BUN values are shown for each mouse. pTFEB indicated by *, TFEB by *.

B. An increase in active and total β-catenin levels is observed in Six2Cre^{+}Tfap2b$^{fl/WT}$ mice (n=5) compared to age-matched WT controls (n=4). P-values were determined by a two-tailed t-test. Graphs represent data as mean ± SEM. Source data are provided as a Source Data File.

C. and D. A Six2Cre^{+}Tfap2b$^{fl/WT}$ mouse with the highest BUN value (270.0) has also the highest increase in active β-catenin, associated with the greatest reduction in NCC and Slc3a1 (shown in A in red font). This inverse correlation between active β-catenin levels and NCC or Slc3a1 levels was consistently observed and shown with Pearson correlation analyses (Pearson r value, two-tailed p-value, and correlation matrices). Normalized to β-actin. N=5 mice/group.

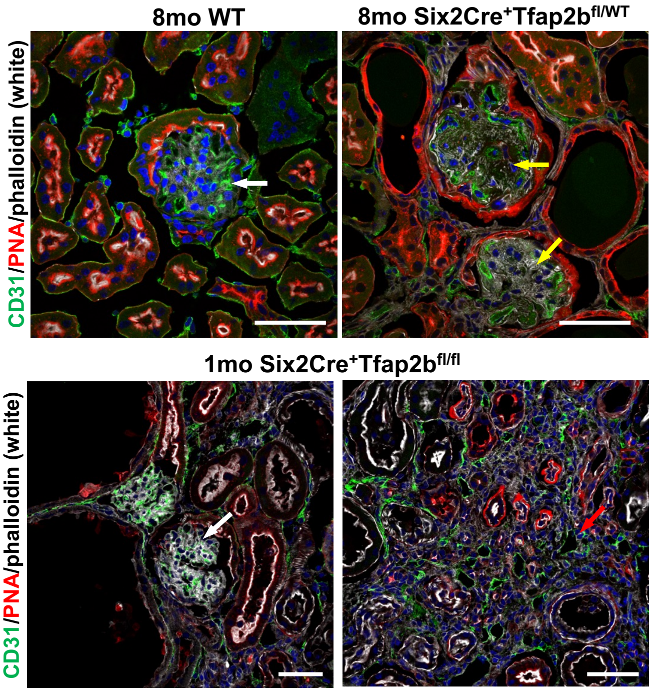

Figure S15: Glomerulosclerosis in aged Six2Cre^{+}Tfap2b$^{fl/WT}$ mouse kidneys leads to diminished glomerular tufts.

Immunolabeling of vessels (CD31^{+}) shows that the extensive glomerulosclerosis in aged Six2Cre^{+}Tfap2b$^{fl/WT}$ mouse kidneys (8-months-old) leads to diminished glomerular tufts (yellow arrows), whereas age-matched WT or 1-months-old Six2Cre^{+}Tfap2b$^{fl/fl}$ mouse kidneys show normal glomerular tufts (white arrows). Irregularly dilated vessels in tubulointerstitial areas of the renal cortex are observed in 1-months-old Six2Cre^{+}Tfap2b$^{fl/fl}$ mouse kidneys (red arrow). Scale bars, 50 μm.

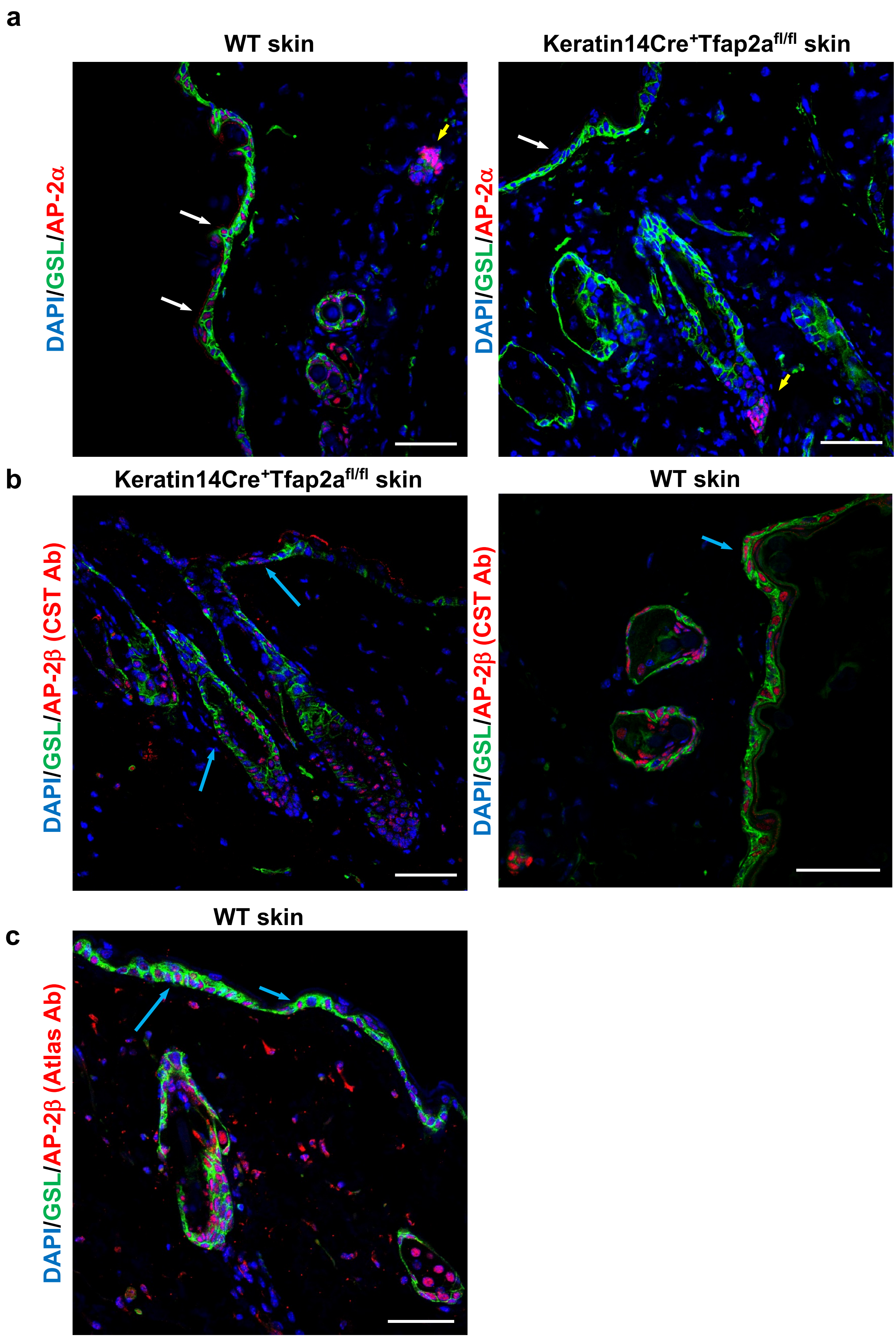
a
WT skin
Keratin14Cre+Tfap2afl/fl skin
DAPI/GSL/AP-2α
DAPI/GSL/AP-2α
b
Keratin14Cre+Tfap2afl/fl skin
WT skin
DAPI/GSL/AP-2β (CST Ab)
DAPI/GSL/AP-2β (CST Ab)
c
WT skin
DAPI/GSL/AP-2β (Atlas Ab)

Figure S16

Figure S16: Immunolabeling detects expression of AP-2α and AP-2β in the mouse epidermis.

AP-2α and AP-2β are expressed in the mouse epidermis and, thus, detection of immunolabeling in the mouse epidermis can provide validation of antibody specificity[2,3].

A. Immunolabeling with the anti-AP-2α antibody (rabbit anti-AP-2α; Abcam Cat# ab108311) detects nuclear AP-2α in keratinocytes of the mouse epidermis (white arrows) as well as in the dermal papilla (yellow arrows). Specificity of the immunolabeling is confirmed by the absence of AP-2α immunolabeling in keratinocytes from skin form mice in which AP-2α was inactivated in keratinocytes (Keratin14Cre^{+}Tfap2a$^{fl/fl}$ mice). Immunolabeling in the dermal papilla persists in Keratin14Cre^{+}Tfap2a$^{fl/fl}$ mice, which is not targeted by the Keratin14Cre, therefore, serving as a positive control for the immunolabeling within the same slide.

B. Immunolabeling for AP-2β is detected in mouse keratinocytes (blue arrows) with the rabbit anti-AP-2β antibody (Cell Signaling Technology Cat# 2509). AP-2β immunolabeling is also detected in keratinocytes of Keratin14Cre^{+}Tfap2a$^{fl/fl}$ mice, thereby ruling out potential cross-reactivity of this antibody with AP-2α.

C. Immunolabeling for AP-2β is also detected in mouse keratinocytes (blue arrows) with the anti-AP-2β antibody from Atlas (Atlas Antibodies Cat# HPA034683, RRID:AB_10670966), recapitulating the findings with the anti-AP-2β antibody from Cell Signaling Technology.

Scale bars, 50µm.

Supplementary Table 1

	age	URINE FE Na	FE K	FE Cl	FE Ca	Fe P	FE Mg	FE Urea Nitrogen	Urine Na / Urine Creatinine	Urine K / Urine Creatinine	Urine Cl / Urine Creatinine	Urine Ca / Urine Creatinine	Urine P / Urine Creatinine	Urine Mg / Urine Creatinine	Urine N / Urine Creatinine	Urine Flow Rate (µl/min)	Creatinine Clearance (µl/min)
WT	18mo (n=4)	0.31	17.64	0.58	0.21	12.09	7.91	63.61	2.08	6.56	2.79	0.11	4.25	1.09	81.63	1.15	241.14
PvalbCre+Tfap2b$^{fl/fl}$	18mo (n=6)	0.43	19.03	0.69	0.23	16.59	11.64	77.83	2.02	5.69	2.68	0.09	4.83	1.04	80.20	1.81	173.38
t-test		0.5704605	0.8076353	0.6172978	0.777689	0.4533267	0.1548941	0.4722908	0.92600206	0.4879229	0.8696205	0.543102045	0.2416315	0.8455575	0.8827759	0.5911217	0.5498655
WT	9mo (n=5)	0.41	20.32	0.70	0.47	8.02	6.93	377.30	2.31	6.57	2.97	0.21	2.72	0.66	485.88	0.47	178.70
Aqp2Cre+Tfap2a$^{fl/fl}$	9mo (n=5)	0.33	15.07	0.66	0.38	4.49	6.12	56.25	1.90	6.32	2.89	0.19	1.90	0.75	77.83	0.50	193.81
Six2Cre+Tfap2a$^{fl/fl}$	9mo (n=4)	0.38	15.68	0.76	0.45	14.19	9.18	52.06	2.33	5.63	3.43	0.20	5.37	0.92	82.19	0.77	175.80
ttest WT vs Aqp2Cre+ Tfap2a$^{fl/fl}$		0.514488	0.4662404	0.8492388	0.4057156	0.1058297	0.6963253	0.3320796	0.121703142	0.7598738	0.7646763	0.772678812	0.0942551	0.641613	0.3432485	0.8329845	0.7023739
ttest WT vs Six2Cre+ Tfap2a$^{fl/fl}$		0.8146606	0.584034	0.8065079	0.8005522	0.0584771	0.5189538	0.3869322	0.926139157	0.2886015	0.0638891	0.893683934	0.0141494	0.274335	0.4085857	0.0478368	0.9277493
WT	15mo (n=6)	0.60	27.75	1.16	0.48	19.44	11.49	110.92	2.77	7.23	4.01	0.16	5.16	1.13	88.65	1.40	181.50
Six2Cre+Tfap2b$^{fl/WT}$	15mo (n=4)	0.64	33.91	1.17	0.60	22.19	11.99	69.82	2.27	7.13	3.18	0.16	4.17	0.93	89.23	2.30	247.99
t-test		0.9011739	0.6416766	0.9938928	0.6579076	0.8387313	0.9224174	0.1496577	0.454747119	0.913273	0.3071087	0.990410363	0.4414825	0.3585893	0.9530886	0.1280416	0.5302337

	age	BLOOD Albumin (g/dl)	Calcium (mg/dl)	Chloride (mEq/l)	Creatinine (mg/dl)	Globulin (g/dl)	Magnesium (mg/dl)	Na/K Ratio	Phosphorus (mg/dl)	Potassium (mEq/l)	Sodium (mEq/l)	Total Protein (g/dl)	URINE Ca mass$_U$	P mass$_U$	Cl mass$_U$	Na mass$_U$	K mass$_U$	Mg mass$_U$	Total Urine 24hr (µl)
WT	18mo (n=4)	2.88	10.95	107.25	0.23	2.80	3.08	17.75	8.23	8.63	150.00	5.68	0.06	3.26	8.02	3.82	20.71	1.16	1702.50
PvalbCre+Tfap2b$^{fl/fl}$	18mo (n=6)	2.46	10.93	109.67	0.28	2.58	2.47	18.33	8.87	8.27	150.50	5.04	0.04	3.32	6.81	3.17	16.00	0.91	2406.67
t-test		0.2444743	0.9773992	0.2158349	0.380461	0.5207972	0.0640334	0.6969915	0.292293146	0.5816991	0.7799835	0.283038612	0.4691211	0.9110229	0.6440673	0.5947823	0.4302086	0.5416941	0.603206285
													0	0	0	0	0	0	
WT	9mo (n=5)	2.84	10.32	113.20	0.26	2.43	2.50	17.00	8.70	9.06	150.60	5.33	0.20	1.60	7.14	3.60	18.41	0.61	690.00
Aqp2Cre+Tfap2a$^{fl/fl}$	9mo (n=5)	2.96	11.78	114.40	0.26	2.85	2.86	13.60	10.68	11.16	149.60	5.80	0.18	1.46	8.01	3.40	19.55	0.63	756.00
Six2Cre+Tfap2a$^{fl/fl}$	9mo (n=4)	2.70	10.10	116.00	0.30	3.15	2.60	17.50	8.05	8.70	153.50	5.85	0.04	1.73	4.67	1.75	9.72	0.57	1157.50
ttest WT vs Aqp2Cre+ Tfap2a$^{fl/fl}$		0.4626499	0.0006097	0.801875	1	0.1374985	0.0234083	0.0835372	0.085643774	0.082745	0.7175744	0.343542624	0.5955128	0.5851382	0.5233882	0.9661499	0.5652938	0.6304098	0.831251435
ttest WT vs Six2Cre+ Tfap2a$^{fl/fl}$		0.0529985	0.7519908	0.8033375	0.8943301	0.1241799	0.0995592	0.4589607	0.539950182	0.4645972	0.9752256	0.842210351	0.063506	0.0346868	0.53889	0.9100295	0.6823433	0.3971706	0.084884912
WT	15mo (n=6)	2.48	11.18	115.33	0.32	3.18	3.03	18.83	8.50	8.47	154.00	5.55	0.06	3.74	9.51	4.40	19.47	0.98	1956.67
Six2Cre+Tfap2b$^{fl/WT}$	15mo (n=4)	2.07	11.15	113.50	0.40	2.40	3.28	17.50	8.78	8.70	152.25	4.47	0.05	4.28	10.74	5.04	27.26	1.15	3377.50
t-test		0.2480863	0.9709023	0.4018058	0.5640595	0.1448785	0.376825	0.5412462	0.892163253	0.8247001	0.471049	0.190787852	0.6591509	0.7294591	0.6469904	0.6747564	0.1418176	0.6249834	0.121177512

	age	URINE Sodium (mEq/l)	Potassium (mEq/l)	Chloride (mEq/l)	Creatinine (mg/dl)	Calcium (mg/dl)	Phosphate (mg/dl)	Magnesium (mEq/l)	Osmolality (mOsm/kgH20)
WT	18mo (n=4)	115.75	345.75	150.25	54.18	5.65	233.63	51.90	2334.75
PvalbCre+Tfap2b$^{fl/fl}$	18mo (n=6)	80.83	245.30	116.33	44.90	4.20	207.23	43.88	1853.83
t-test		0.3748643	0.2852307	0.5034284	0.5362539	0.4691211	0.6908917	0.5866326	0.424005914
WT	9mo (n=5)	226.20	660.94	291.40	100.18	21.06	255.62	64.06	3986.25
Aqp2Cre+Tfap2a$^{fl/fl}$	9mo (n=5)	191.60	630.00	288.80	98.50	17.72	191.32	75.98	4045.00
Six2Cre+Tfap2a$^{fl/fl}$	9mo (n=4)	131.50	427.50	228.00	65.55	8.85	297.40	80.55	2835.00
ttest WT vs Aqp2Cre+ Tfap2a$^{fl/fl}$		0.3823662	0.8249738	0.9571038	0.9105593	0.5955128	0.0898949	0.5998353	0.936957874
ttest WT vs Six2Cre+ Tfap2a$^{fl/fl}$		0.0091509	0.0453677	0.0400125	0.0278005	0.063506	0.2919668	0.6760725	0.079494868
WT	15mo (n=6)	107.00	290.58	158.17	39.83	5.65	202.95	45.07	1939.20
Six2Cre+Tfap2b$^{fl/WT}$	15mo (n=4)	74.25	248.13	112.50	34.00	5.15	120.58	27.60	1640.50
t-test		0.2368149	0.617886	0.3294842	0.5376969	0.6591509	0.17396	0.1240909	0.593757066

Table S1: Urinary and serum electrolyte measurements in groups of PvalbCre+Tfap2b$^{fl/fl}$ mice, Aqp2Cre+Tfap2a$^{fl/fl}$ mice, Six2Cre+Tfap2b$^{fl/WT}$ mice, Six2Cre+Tfap2a$^{fl/fl}$ mice and age-matched WT controls.
Urinary sodium, potassium, chloride and magnesium in mEq/l. Urinary creatinine, calcium, and phosphate in mg/dl. Urine osmolality in mOsm/kg$H_2$0. Creatinine clearance = urine creatinine x urine flow (ml/min) / serum creatinine. FE electrolytes = 100x (([electrolyte urine] x [creatinine serum])/([electrolyte serum] x [creatinine urine])). Serum creatinine, calcium, phosphorous and magnesium in mg/dl. Serum sodium, potassium, and chloride in mEq/l. Mass of electrolytes in 24-hour urine collections shown in µg. P-values are shown (two-tailed, unpaired *t*-test).

		Na	K	Cl	Na/K ratio	Calcium	Phosphorus	Mg	Albumin	TP	globulin
control	2-4mo(n=14)	152.86	9.26	117.86	16.93	11.67	9.61	2.63	2.65	5.21	2.56
Six2Cre^{+}Tfap2a$^{fl/fl}$	2-4mo(n=5)	153.40	8.86	114.40	17.40	12.26	9.72	2.96	2.70	5.26	2.56
ttest		0.824739	0.583649	0.229086	0.753038942	0.157862	0.87509177	0.127564	0.719579	0.83167	0.978815
		Na	K	Cl	Na/K ratio	Calcium	Phosphorus	Mg	Albumin	TP	globulin
control	5-8mo(n=3)	162.00	10.20	122.67	16.30	12.70	10.00	3.13	2.63	5.87	3.23
Six2Cre^{+}Tfap2a$^{fl/fl}$	5-8mo(n=6)	151.33	9.15	113.50	16.67	10.95	7.53	2.58	2.75	5.63	2.72
ttest		0.0828	0.332044	0.096778	0.797427697	0.000978	0.018306817	0.010635	0.744811	0.487574	0.000415
		Na	K	Cl	Na/K ratio	Calcium	Phosphorus	Mg	Albumin	TP	globulin
control	9-12mo(n=6)	153.17	9.28	116.33	16.67	11.97	10.48	3.23	2.65	6.02	3.37
Six2Cre^{+}Tfap2a$^{fl/fl}$	9-12mo(n=6)	153.83	9.13	114.17	16.83	11.07	8.50	2.88	2.67	5.47	2.80
ttest		0.881656	0.836274	0.549014	0.901928319	0.378086	0.049437917	0.286707	0.950876	0.083804	0.010983
		Na	K	Cl	Na/K ratio	Calcium	Phosphorus	Mg	Albumin	TP	globulin
control	13-16mo(n=6)	157.00	8.32	118.00	19.17	11.47	7.75	3.02	2.38	5.13	2.75
Six2Cre^{+}Tfap2a$^{fl/fl}$	13-16mo(n=4)	156.50	10.05	117.50	15.50	10.28	8.25	3.10	2.55	5.60	3.05
ttest		0.921406	0.021059	0.921406	0.02775452	0.008793	0.57331864	0.766175	0.559459	0.385078	0.338141
		Na	K	Cl	Na/K ratio	Calcium	Phosphorus	Mg	Albumin	TP	globulin
control	2-4mo(n=14)	152.86	9.26	117.86	16.93	11.67	9.61	2.63	2.65	5.21	2.56
Six2Cre^{+}Tfap2b$^{fl/WT}$	2-4mo(n=3)	155.00	8.60	113.33	18.00	11.90	9.23	2.93	2.53	4.93	2.40
ttest		0.506696	0.4699	0.188928	0.545023516	0.661565	0.645366206	0.284611	0.533494	0.336239	0.448624
		Na	K	Cl	Na/K ratio	Calcium	Phosphorus	Mg	Albumin	TP	globulin
control	5-8mo(n=3)	162.00	10.20	122.67	16.30	12.70	10.00	3.13	2.63	5.87	3.23
Six2Cre^{+}Tfap2b$^{fl/WT}$	5-8mo(n=7)	149.00	8.20	107.57	18.57	10.91	8.87	3.09	2.77	5.53	2.76
ttest		0.032841	0.146302	0.015016	0.336326931	0.038436	0.471294629	0.897986	0.727581	0.415198	0.01195
		Na	K	Cl	Na/K ratio	Calcium	Phosphorus	Mg	Albumin	TP	globulin
control	9-12mo(n=5)	154.60	9.44	117.60	16.60	12.36	10.56	3.38	2.76	6.04	3.28
Six2Cre^{+}Tfap2b$^{fl/WT}$	9-12mo(n=8)	153.38	8.33	113.50	18.38	11.24	8.33	3.24	2.54	5.34	2.80
ttest		0.763339	0.099084	0.29917	0.168492641	0.170914	0.070057154	0.733769	0.416502	0.072982	0.030578
		Na	K	Cl	Na/K ratio	Calcium	Phosphorus	Mg	Albumin	TP	globulin
control	13-16mo(n=6)	157.00	8.32	118.00	19.17	11.47	7.75	3.02	2.38	5.13	2.75
Six2Cre^{+}Tfap2b$^{fl/WT}$	13-16mo(n=8)	157.00	8.64	117.63	18.50	12.03	8.93	3.35	2.50	5.21	2.71
ttest		1	0.673499	0.938626	0.650762577	0.521335	0.28858531	0.34192	0.531404	0.780082	0.782196
		Na	K	Cl	Na/K ratio	Calcium	Phosphorus	Mg	Albumin	TP	globulin
control	2-4mo(n=14)	152.86	9.26	117.86	16.93	11.67	9.61	2.63	2.65	5.21	2.56
Six2Cre^{+}Tfap2a$^{fl/fl}$Tfap2b$^{fl/WT}$	2-4mo(n=5)	149.20	8.64	92.36	17.40	11.02	8.92	2.44	2.80	5.56	2.76
ttest		0.071389	0.406929	0.044934	0.745727246	0.099535	0.252800562	0.348951	0.338847	0.125887	0.249056
		Na	K	Cl	Na/K ratio	Calcium	Phosphorus	Mg	Albumin	TP	globulin
control	5-8mo(n=3)	162.00	10.20	122.67	16.30	12.70	10.00	3.13	2.63	5.87	3.23
Six2Cre^{+}Tfap2a$^{fl/fl}$Tfap2b$^{fl/WT}$	5-8mo(n=7)	153.89	9.51	115.44	16.33	11.64	9.17	2.92	2.96	5.86	2.90
ttest		0.268198	0.428337	0.289692	0.980818169	0.345917	0.61911439	0.527668	0.433536	0.977981	0.089393
		Na	K	Cl	Na/K ratio	Calcium	Phosphorus	Mg	Albumin	TP	globulin
control	9-12mo(n=5)	154.60	9.44	117.60	16.60	12.36	10.56	3.38	2.76	6.04	3.28
Six2Cre^{+}Tfap2a$^{fl/fl}$Tfap2b$^{fl/WT}$	9-12mo(n=9)	155.44	8.70	115.56	18.56	11.39	8.79	2.73	2.57	5.21	2.64
ttest		0.858542	0.496423	0.662164	0.291996959	0.242429	0.107250192	0.034774	0.46505	0.019385	0.001871
		Na	K	Cl	Na/K ratio	Calcium	Phosphorus	Mg	Albumin	TP	globulin
control	13-16mo(n=6)	157.00	8.32	118.00	19.17	11.47	7.75	3.02	2.38	5.13	2.75
Six2Cre^{+}Tfap2a$^{fl/fl}$Tfap2b$^{fl/WT}$	13-16mo(n=10)	158.70	9.25	118.70	17.40	11.23	7.76	3.08	2.52	5.42	2.90
ttest		0.739815	0.151523	0.878885	0.172390948	0.378441	0.98955137	0.746952	0.438849	0.315997	0.324954
		Na	K	Cl	Na/K ratio	Calcium	Phosphorus	Mg	Albumin	TP	globulin
control	2-4mo(n=2)	147.50	9.15	111.50	16.50	11.05	10.10	2.50	3.00	5.65	2.65
PvalbCre^{+}Tfap2b$^{fl/fl}$	2-4mo(n=4)	148.25	8.05	111.25	18.75	10.80	7.90	1.95	3.23	5.90	2.68
ttest		0.632813	0.275673	0.632813	0.341850962	0.798203	0.183059179	0.221318	0.625262	0.687919	0.917261
		Na	K	Cl	Na/K ratio	Calcium	Phosphorus	Mg	Albumin	TP	globulin
control	5-8mo(n=5)	151.40	7.38	109.60	20.60	10.66	7.72	2.94	2.66	5.22	2.56
PvalbCre^{+}Tfap2b$^{fl/fl}$	5-8mo(n=12)	150.75	7.89	109.83	19.33	11.50	7.54	2.34	2.79	5.73	2.94
ttest		0.275337	0.358377	0.866706	0.330189592	0.126038	0.849476537	0.000794	0.257073	0.001951	0.006175
		Na	K	Cl	Na/K ratio	Calcium	Phosphorus	Mg	Albumin	TP	globulin
control	9-12mo(n=3)	150.67	10.77	114.33	14.33	12.93	11.33	3.37	2.67	5.77	3.10
PvalbCre^{+}Tfap2b$^{fl/fl}$	9-12mo(n=6)	151.17	8.03	111.50	19.17	11.90	8.00	2.67	2.93	5.67	2.73
ttest		0.51649	0.013168	0.076936	0.018973168	0.123767	0.017930497	0.024416	0.278077	0.795955	0.133444
		Na	K	Cl	Na/K ratio	Calcium	Phosphorus	Mg	Albumin	TP	globulin
control	2-4mo(n=4)	153.75	8.08	115.25	19.00	10.85	7.73	3.05	2.83	5.43	2.60
Aqp2Cre^{+}Tfap2b$^{fl/fl}$	2-4mo(n=4)	155.50	7.70	113.75	20.50	11.28	8.75	3.30	2.70	5.40	2.70
ttest		0.743053	0.429041	0.74541	0.25437354	0.30162	0.255425099	0.214843	0.532399	0.92135	0.675384
		Na	K	Cl	Na/K ratio	Calcium	Phosphorus	Mg	Albumin	TP	globulin
control	5-8mo(n=4)	149.50	7.33	110.00	21.00	11.55	7.65	2.70	2.55	5.25	2.70
Aqp2Cre^{+}Tfap2b$^{fl/fl}$	5-8mo(n=4)	151.50	8.55	114.00	18.00	11.68	8.60	3.03	2.73	5.65	2.93
Aqp2Cre^{+}Tfap2a$^{fl/fl}$	5-8mo(n=2)	151.00	8.50	111.00	18.00	12.35	8.75	2.45	2.55	5.30	2.75
ttest (control vs Aqp2Cre^{+}Tfap2b$^{fl/fl}$)		0.0134	0.274193	0.0134	0.266569703	0.561814	0.009506134	0.104731	0.127385	0.167714	0.355918
ttest (control vs Aqp2Cre^{+}Tfap2a$^{fl/fl}$)		0.355232	0.428978	0.764806	0.414430083	0.12255	0.044758834	0.250617	1	0.835026	0.844599

Table S2: Serum chemistries in experimental mouse groups of different ages.
Chloride in mEq/l, potassium in mEq/l, sodium in mEq/l, magnesium in mg/dl, phosphorus in mg/dl, total protein in g/dl, albumin in g/dl, calcium in mg/dl, and globulin in g/dl. P-values are shown (two-tailed, unpaired *t*-test).

Supplementary References:

1 Ransick, A. *et al.* Single-Cell Profiling Reveals Sex, Lineage, and Regional Diversity in the Mouse Kidney. *Dev Cell* **51**, 399-413 e397, doi:10.1016/j.devcel.2019.10.005 (2019).

2 Wang, X. *et al.* AP-2alpha: a regulator of EGF receptor signaling and proliferation in skin epidermis. *The Journal of cell biology* **172**, 409-421, doi:10.1083/jcb.200510002 (2006).

3 Wang, X., Pasolli, H. A., Williams, T. & Fuchs, E. AP-2 factors act in concert with Notch to orchestrate terminal differentiation in skin epidermis. *The Journal of cell biology* **183**, 37-48, doi:10.1083/jcb.200804030 (2008).
