# Supplementary material for: Transcription factors AP-2α and AP-2β regulate distinct segments of the distal nephron in the mammalian kidney
Source: Nat Commun. 2022 Apr 25;13:2226. doi: 10.1038/s41467-022-29644-3 (PMC9038906; doi:10.1038/s41467-022-29644-3)
Supplement: Supplementary file 2 — Reporting Summary [file 41467_2022_29644_MOESM2_ESM.pdf]

nature portfolio

Corresponding author(s): Alexander G. Marneros
Last updated by author(s): March 18th, 2022

Reporting Summary

Nature Portfolio wishes to improve the reproducibility of the work that we publish. This form provides structure for consistency and transparency in reporting. For further information on Nature Portfolio policies, see our Editorial Policies and the Editorial Policy Checklist.

Statistics

For all statistical analyses, confirm that the following items are present in the figure legend, table legend, main text, or Methods section.

n/a	Confirmed	
☐	☒	The exact sample size (*n*) for each experimental group/condition, given as a discrete number and unit of measurement
☐	☒	A statement on whether measurements were taken from distinct samples or whether the same sample was measured repeatedly
☐	☒	The statistical test(s) used AND whether they are one- or two-sided<br>*Only common tests should be described solely by name; describe more complex techniques in the Methods section.*
☒	☐	A description of all covariates tested
☐	☒	A description of any assumptions or corrections, such as tests of normality and adjustment for multiple comparisons
☐	☒	A full description of the statistical parameters including central tendency (e.g. means) or other basic estimates (e.g. regression coefficient) AND variation (e.g. standard deviation) or associated estimates of uncertainty (e.g. confidence intervals)
☐	☒	For null hypothesis testing, the test statistic (e.g. *F*, *t*, *r*) with confidence intervals, effect sizes, degrees of freedom and *P* value noted<br>*Give P values as exact values whenever suitable.*
☒	☐	For Bayesian analysis, information on the choice of priors and Markov chain Monte Carlo settings
☒	☐	For hierarchical and complex designs, identification of the appropriate level for tests and full reporting of outcomes
☐	☒	Estimates of effect sizes (e.g. Cohen's *d*, Pearson's *r*), indicating how they were calculated

Our web collection on statistics for biologists contains articles on many of the points above.

Software and code

Policy information about availability of computer code

Data collection	Only published software/code were used. For RNA-Seq analysis: Sequencing reads were mapped in a splice-aware fashion to the Ensembl annotation of the mouse GRCm37/mm9 transcriptome using STAR. Read counts over transcripts were calculated using HTSeq, followed by the differential expression analysis using EdgeR.
Data analysis	Only published software/code were used. Statistical analyses were perform with Prism 9.3.1 (Graphpad).

For manuscripts utilizing custom algorithms or software that are central to the research but not yet described in published literature, software must be made available to editors and reviewers. We strongly encourage code deposition in a community repository (e.g. GitHub). See the Nature Portfolio guidelines for submitting code & software for further information.

Data

Policy information about availability of data

All manuscripts must include a data availability statement. This statement should provide the following information, where applicable:

- Accession codes, unique identifiers, or web links for publicly available datasets
- A description of any restrictions on data availability
- For clinical datasets or third party data, please ensure that the statement adheres to our policy

For RNA-Seq data analysis, sequencing reads were mapped in a splice-aware fashion to the Ensembl annotation of the mouse GRCm37/mm9 transcriptome.

Data and Code Availability: RNA-Seq data has been deposited to the GEO database: GSE126326 [https://www.ncbi.nlm.nih.gov/geo/query/acc.cgi?acc=GSE126326]

and GSE130864 [https://www.ncbi.nlm.nih.gov/geo/query/acc.cgi?acc=GSE130864].

Field-specific reporting

Please select the one below that is the best fit for your research. If you are not sure, read the appropriate sections before making your selection.

- [x] Life sciences
- [ ] Behavioural & social sciences
- [ ] Ecological, evolutionary & environmental sciences

For a reference copy of the document with all sections, see nature.com/documents/nr-reporting-summary-flat.pdf

Life sciences study design

All studies must disclose on these points even when the disclosure is negative.

Sample size	Sample size descriptions are made in the METHODS part and in the figure legends or exact mouse numbers used are listed in brackets in graphs. Sample sizes were based on our previously published data on kidney phenotypes in AP-2beta/KCTD1 mouse mutants (Marneros, Dev Cell, 2020).
Data exclusions	no data exclusions were made
Replication	replication of data is specified for each experiment in the METHODS or figure legends.
Randomization	mouse studies: mice were randomly assigned to specific groups
Blinding	mouse studies: investigators were blinded and did not know the genotype of the mice when performing experiments

Reporting for specific materials, systems and methods

We require information from authors about some types of materials, experimental systems and methods used in many studies. Here, indicate whether each material, system or method listed is relevant to your study. If you are not sure if a list item applies to your research, read the appropriate section before selecting a response.

Materials & experimental systems

n/a	Involved in the study	
[ ]	[x]	Antibodies
[x]	[ ]	Eukaryotic cell lines
[x]	[ ]	Palaeontology and archaeology
[ ]	[x]	Animals and other organisms
[x]	[ ]	Human research participants
[x]	[ ]	Clinical data
[x]	[ ]	Dual use research of concern

Methods

n/a	Involved in the study	
[x]	[ ]	ChIP-seq
[x]	[ ]	Flow cytometry
[x]	[ ]	MRI-based neuroimaging

Antibodies

Antibodies used

For immunolabelings the following antibodies were used:

The following primary antibodies were used at a 1:100 dilution except were indicated: rabbit anti-AP-2a (Abcam Cat# ab108311, RRID:AB_10861200; 1:200 dilution; validated by absence of nuclear staining in inner medullary CDs of Aqp2Cre+Tfap2afl/fl mice or absence of nuclear staining in keratinocytes of Keratin14Cre+Tfap2afl/fl mice [Supplementary Fig. S16]), rabbit anti-AP-2b (Cell Signaling Technology Cat# 2509, RRID:AB_2058198; 1:200 dilution), rabbit anti-AP-2b (Atlas Antibodies Cat# HPA034683, RRID:AB_10670966), rat anti-F4/80 (conjugated with Alexa647, BioLegend Cat# 123121, RRID:AB_893492), rabbit anti-Slc3a1 (Proteintech Cat# 16343-1-AP, RRID:AB_2239419), mouse anti-Calb1 (Sigma Aldrich Cat# C9848, RRID:AB_476894; 1:300 dilution), goat anti-Aqp2 (Santa Cruz Biotechnology Cat# sc-9882, RRID:AB_2289903; 1:500 dilution), rabbit anti-NCC (Millipore Cat# AB3553, RRID:AB_571116), rabbit anti-NKCC2 (Cell Signaling Technologies Cat# 38436, RRID:AB_2799134), rabbit anti-parvalbumin (Abcam Cat# ab11427, RRID:AB_298032; 1:200 dilution), rat anti-PDGFRβ (Thermo Fisher Scientific Cat# 14-1402-82, RRID:AB_467493), rat anti-CD45 (BD Pharmingen (550539), rat anti-CD133 (14-1331-80, ebioscience; 1:200 dilution), rabbit anti-TFEB (Bethyl Cat# A303-672A, RRID:AB_11204598; 1:200 dilution), rabbit anti-Aqp3 (Sigma-Aldrich Cat# A0303, RRID:AB_257878; 1:200 dilution), goat anti-EGF (R&D Systems Cat# AF2028, RRID:AB_355111), rabbit anti-phospho 4E-BP1 (Thr37/46) (Cell Signaling Technology Cat# 2855, RRID:AB_560835; 1:200 dilution), rabbit anti-cleaved caspase-3 (Asp175) (Cell Signaling Technology Cat# 9664, RRID:AB_2070042), chicken anti-GFP (Rockland Cat# 600-901-215, RRID:AB_1537402; 1:200 dilution), rabbit anti-GFP (Thermo Fisher Scientific Cat# A-11122, RRID:AB_221569), rabbit anti-Ki67 (Abcam Cat# ab16667, RRID:AB_302459) antibodies, Cy3-conjugated mouse monoclonal SMA (clone 1A4) (Sigma-Aldrich Cat# A2547, RRID:AB_476701: 1:400 dilution) antibodies, rat anti-CD31 (BD Biosciences Cat# 550274, RRID:AB_393571), and Alexa647-conjugated mouse anti-V-ATPase B1/B2 antibodies (Santa Cruz Biotechnology Cat# sc-55544 AF647, RRID:AB_831844). Phalloidin conjugated with Alexa488 (Molecular Probes Cat# A-12379, RRID:AB_2315147) or Alexa647 (Thermo Fisher Scientific Cat# A22287, RRID:AB_2620155) were used for cytoskeletal staining at a dilution of 1:100. DAPI was used to stain

nuclei (Thermo Fisher Scientific Cat# D3571, RRID:AB_2307445). Secondary Alexa-488/555/647 antibodies were used at a dilution of 1:200 (Thermo Fisher). Controls included stainings with no primary antibody or with IgG control primary antibodies. Lectins at a dilution of 1:200 were from Vector Laboratories: rhodamine-conjugated peanut agglutinin [PNA] (Vector Laboratories Cat# RL-1072, RRID:AB_2336642) [epithelial staining of distal nephron epithelial cells; contiguous staining of PTs], fluorescein-conjugated Lotus Tetragonolobus Lectin [LTL] (Vector Laboratories Cat# FL-1321, RRID:AB_2336559) [labels PTs], rhodamine-conjugated wheat germ agglutinin [WGA] (Vector Laboratories Cat# RL-1022, RRID:AB_2336871).

For Western blotting the following antibodies were used: The following primary antibodies were used at a 1:1000 except were indicated: goat anti-Aqp2 (Santa Cruz Biotechnology Cat# sc-9882, RRID:AB_2289903; 1:200 dilution), rabbit anti-NKCC2 (38436, Cell Signaling Technology), rabbit anti-NCC (Millipore Cat# AB3553, RRID:AB_571116; 1:2000), rabbit anti-active b-catenin (non-phospho Ser33/37/Thr41) (Cell Signaling Technology Cat# 8814S, RRID:AB_11127203), rabbit anti-total GSK-3b (Cell Signaling Technology Cat# 9315, RRID:AB_490890), rabbit anti-total GSK-3b (Cell Signaling Technology Cat# 12456, RRID:AB_2636978), rabbit anti-total 4E-BP1 (Cell Signaling Technology Cat# 9644, RRID:AB_2097841), rabbit anti-phospho 4E-BP1 (Thr37/46) (Cell Signaling Technology Cat# 2855, RRID:AB_560835), rabbit anti-p70 S6 kinase (49D7) (Cell Signaling Technology Cat# 2708, RRID:AB_390722), rabbit anti-TFEB (Bethyl Cat# A303-672A, RRID:AB_11204598), rabbit anti-Pvalb (Abcam Cat# ab11427, RRID:AB_298032; 1:2000 dilution), and rabbit anti-Slc3a1 (Proteintech Cat# 16343-1-AP, RRID:AB_2239419; 1:800 dilution). HRP-conjugated secondary antibodies were used at a 1:2000 dilution (HRP-linked anti-rabbit IgG from Cell Signaling Technology Cat#7074S, RRID:AB_2099233; HRP-linked anti-goat IgG from Santa Cruz Biotechnology Cat#sc-2020, RRID_AB_631728) and chemiluminescence signal was determined with the SuperSignal WestPico chemiluminescent substrate (Pierce).
Equal protein loading was assessed using a rabbit polyclonal anti-b-actin antibody (Cell Signaling Technology Cat# 4970, RRID:AB_2223172).

Validation

All used antibodies are commercially available. We validated the specificity of AP-2alpha antibodies in this manuscript in mouse skin of mice lacking AP-2alpha in the epidermis or in kidneys lacking AP-2alpha in medullary ducts.

Animals and other organisms

Policy information about studies involving animals; ARRIVE guidelines recommended for reporting animal research

Laboratory animals

Tfap2afl/fl mice have previously been reported: Cre activity results in removal of exons 5 and 6 that are required for DNA binding activity. Tfap2bfl/fl mice have previously been described as well: Cre-mediated recombination results in removal of exon 6 that is critical for DNA binding, resulting in AP-2b without transcription factor activity. We showed by RNA-Seq of kidney lysates from TAM-treated b-actinCreERT2+Tfap2bfl/fl mice efficient Cre-mediated removal of exon 6 of Tfap2b. Similarly, we confirmed this by RNA-Seq of whole kidneys of Six2Cre+Tfap2afl/flTfap2bfl/WT mice that shows the presence of AP-2b transcripts in these kidneys with a reduction of exon 6 due to conditional removal of the heterozygous floxed exon 6 despite inactivation of AP-2a. The following Cre lines were used: Six2Cre+ mice [IMSR Cat# JAX:009606, RRID:IMSR_JAX:00960627], PvalbCre+ mice (IMSR Cat# JAX:017320, RRID:IMSR_JAX:017320), and Aqp2Cre+ mice [IMSR Cat# JAX:006881, RRID:IMSR_JAX:006881; using only female Aqp2Cre+ mice for matings]). Cre activity and specificity were confirmed by crossing these strains with B6.Cg-Gt(ROSA)26Sortm3(CAG-EYFP)Hze/J (Ai3) reporter mice (IMSR Cat# JAX:007903, RRID:IMSR_JAX:007903). Co-immunolabeling for GFP and Aqp2 showed strict co-localization in Aqp2Cre+Ai3 reporter mice, confirming that Cre activity in these mice is limited to CTs/CDs. Co-immunolabeling for GFP and Pvalb showed strict co-localization in PvalbCre+Ai3 reporter mice, confirming that Cre activity in these mice is limited to DCT1s. We and others also validated that Six2Cre mice target the nephron proximal to the CDs, including the CTs. EGF-/- mice have previously been reported. TCF/Lef:H2B-GFP reporter mice (IMSR Cat# JAX:013752, RRID:IMSR_JAX:013752) were used to assess b-catenin signaling activity in vivo. We also inactivated AP-2a selectively in the epidermis with Keratin14Cre mice (IMSR Cat# JAX:018964, RRID:IMSR_JAX:018964), in order to validate the anti-AP-2a antibody in a tissue with well-established expression AP-2a. For all animal studies, institutional approval (MGH IACUC committee) was granted and international guidelines for the care and use of laboratory research animals were followed. ARRIVE guidelines for reporting of animal studies were followed.
Age and gender of mice used is indicated in the Figures and Figure Legends. Mice are housed in a vivarium with regular day/night cycles.

Wild animals

No wild animals were used in this study.

Field-collected samples

No field-collected samples were used in this study.

Ethics oversight

Massachusetts General Hospital IACUC approval was obtained for these studies

Note that full information on the approval of the study protocol must also be provided in the manuscript.
